# Influence of surgical intervention on pre- and post-surgery patient specific muscle synergies in children with cerebral palsy

**DOI:** 10.1007/s00221-026-07308-8

**Published:** 2026-05-04

**Authors:** Tiana Breust, Jiayin Lin, Vincent C. K. Cheung, Firooz Salami, Sebastian I. Wolf, Gursel Alici, Manish Sreenivasa

**Affiliations:** 1 School of Engineering, Faculty of Engineering and Information Sciences, University of Wollongong, Northfields Avenue, Wollongong, NSW 2522 Australia; 2 School of Biomedical Sciences, and The Gerald Choa Neuroscience Institute, The Chinese University of Hong Kong, Hong Kong, China; 3 Clinic for Orthopaedics, Heidelberg University Hospital, Heidelberg, Germany

**Keywords:** Cerebral palsy, Muscle synergies, Motor control, Non-negative matrix factorisation, Gait

## Abstract

Muscle synergy analysis has been explored as means to better understand changes in motor control, with the aim to improve treatment outcomes after Single Event Multilevel Surgery (SEMLS) for children with cerebral palsy (CP). In this single-group study, we assess changes between pre- and post-surgery muscle synergies at both patient specific, and cohort levels after surgical intervention for children with CP. We analysed the walking data of 11 patients between the ages of 7–18 years old, including surface electromyography, joint angles and ground reaction forces, recorded before and after surgery. Muscle synergies were extracted and assessed for changes in dimensionality and sparseness between pre- and post-surgery. Our results show that at a patient specific level, pre- and post-surgery muscle synergies were not highly similar, with only 41% of paired synergies showing high correlation ($r$ $>$ 0.8) after surgery, and an overall mean correlation of 0.53 $\pm$ 0.25. On the other hand, synergies obtained at the cohort level were highly similar post-surgery ($r$ $>$ 0.87). These findings suggest that at an individual level, pre-surgery motor control cannot be assumed to be preserved post-surgery. CP synergies obtained at the cohort level from pre-surgery data can demonstrate more reliable preservation in post-surgery motor control. We also observed sparseness increasing with the number of synergies, which provides an interesting future research direction exploring the link with more complex motor control and a more typical gait pattern.

**Supplementary Information:**

The online version contains supplementary material available at 10.1007/s00221-026-07308-8.

## Introduction

Current clinical management of children with cerebral palsy (CP) involves a combination of physical therapy, orthotic and/or surgical interventions to improve motor performance and movement function (Damiano et al. 2009). In addressing orthopaedic concerns, single event multilevel surgery (SEMLS) is the standard of care (Gorton et al. 2009; Sung et al. 2013). However, due to the complex nature of SEMLS, the desired results may not always be achieved, and in some cases, patients’ conditions may worsen (Dreher et al. 2007; Filho et al. 2008). This has led research efforts to focus on the analysis of pre-treatment data as a means to understand and predict post-treatment results and subsequently improve patient outcomes (Pitto et al. 2019; Rajagopal et al. 2018). As patient motor control is associated with treatment outcomes (Schwartz et al. 2016), investigating the impact of SEMLS on motor control is an important step. Current research supports the theory that the central nervous system activates groups of muscles as an efficient way to resolve the redundancy of multiple degrees of freedom (d’Avella et al. 2003, 2006; Steele et al. 2015). The co-activated groups of muscles are known as muscle synergies, and the clustered descending commands are the temporal activation profiles acting upon them. Children with CP are known to have fewer and simpler muscle synergies than their age-matched typically developing peers (Tang et al. 2015).

The analysis of muscle synergies allows for a more comprehensive understanding of patient motor control after surgical intervention. High similarity between pre- and post-surgery synergy estimates would align with the current suggestion that motor control remains relatively unchanged (Pitto et al. 2020; Shuman et al. 2019), while lower similarity would suggest that motor control is indeed impacted by surgical intervention. Interpreting changes in the number of synergies is more complex as, while motor control complexity is thought to increase with the number of synergies (Goudriaan et al. 2022), a reduction could also be indicative of improved gait efficiency (Cheung et al. 2020). Quantifying sparseness (Hoyer 2004) of motor control might be useful in this context, with increases in sparseness reflecting more isolated muscle weightings within the synergy structure. Changes in sparseness can then be indicative of complexity changes and prove to be a useful metric (Cheung et al. 2020). It should be noted though that, changes in the synergy dimensionality should be interpreted in line with kinematic changes to ensure that appropriate conclusions are drawn.

Several studies have focused on assessing the changes between pre- and post-surgery in CP patients*. *Shuman et al. (2019) reported minimal changes in CP patients’ motor control after comparing pre- and post-surgery CP muscle synergies to those of typically developing children. Pitto et al. (2020) found that pre-treatment muscle synergies and generic CP synergies could model the post-treatment motor control in CP reasonably well when either the synergy weights or activations were constrained, and the other was allowed to vary to best match the post-treatment data. While muscle synergies of CP children have been compared to those from their typically developing peers (Shuman et al. 2019; Tang et al. 2015), and healthy adults (Tang et al. 2015), it remains an open question as to how much the muscle synergies are changing after surgical intervention at a patient specific level.

While muscle synergies are only one aspect of the complex nature of CP gait, increasing our understanding of possible changes increases their use in future studies. In a study by Pitto et al. (2019) pre-surgery muscle synergies are used to represent the patient motor control in predictive gait simulations designed to aid surgical planning and treatment outcomes. It is subsequently important to ensure that pre-surgery muscle synergies are preserved for all patients after surgical interventions for continued usage and optimised treatment outcomes. Therefore, the novel contribution of this study was to investigate the changes in the muscle synergies of children with CP after SEMLS, at a patient specific level. First, we obtained muscle synergies from pre- and post-surgery data to calculate how similar the muscle synergies were after treatment at the individual level. Second, we investigated how similar the muscle synergies were between patients. We hypothesize that synergy weights and activations will be highly similar after treatment for each patient, and that both pre- and post-surgery synergies are similar between patients.

## Methods

### Participants

We retrospectively analysed the data from 11 patients diagnosed with CP who had undergone SEMLS with pre-surgery and post-surgery clinical gait analysis (Table 1). Common surgeries performed on these patients included distal femur osteotomies and soft tissue procedures (for further details see Online Resource 3a). Patients were reported with a Gross Motor Function Classification System (GMFCS) Level of I-III. The recordings were conducted according to the guidelines of the Declaration of Helsinki 2024 and approved by the ethics committee of the Medical Faculty of Heidelberg University, Germany. Informed consent was obtained from legal guardians of the patients.

**Table 1:** Patient information

	Pre-surgery assessment	Post-surgery assessment
Age (Years)	12.9 ± 2.4	14.3 ± 2.6
Height (cm)	147.6 ± 12.7	156.8 ± 10.0
Mass (kg)	37.8 ± 8.2	45.0 ± 9.5
Time from Assessment to Surgery (Years)	− 0.11 ± 0.19	1.31 ± 0.83
Use of Walking Aid	2 out of 11	
Gender	6 Male, 5 Female	
Diagnosis	9 Diplegic, 2 Hemiplegic	

### Clinical assessment

Each patient underwent clinical gait assessment before and after surgery as part of the routine clinical management protocols. These assessments were performed by highly skilled staff and involved the patient walking barefoot at a self-selected speed. 2 patients used a walking aid (P2 in pre-surgery, and P5 in post-surgery). Patient movement was captured using a 12-camera motion analysis system (Vicon Motion Systems, Oxford, UK) recording at 120 Hz, and 3 ground reaction force plates (Kistler Instruments), recording data at 1080 Hz. Surface electromyography was simultaneously recorded at 1080 Hz bilaterally from 8 major lower limb muscles, using Trigno wireless EMGs (Delsys, Natick, MA, USA), following the Surface Electromyography for the Non-Invasive Assessment of Muscles (SENIAM) guidelines. The muscles recorded were: rectus femoris (RF), vastus lateralis (VL), gluteus medius (GM), semimembranosus (SM), biceps femoris (BF), tibialis anterior (TA), lateral gastrocnemius (LG) and soleus (S). Foot strikes as observed from force plate signals and marker positions were used to segment the EMG data into strides.

### Pre-processing of sEMG

**Fig. 1:**
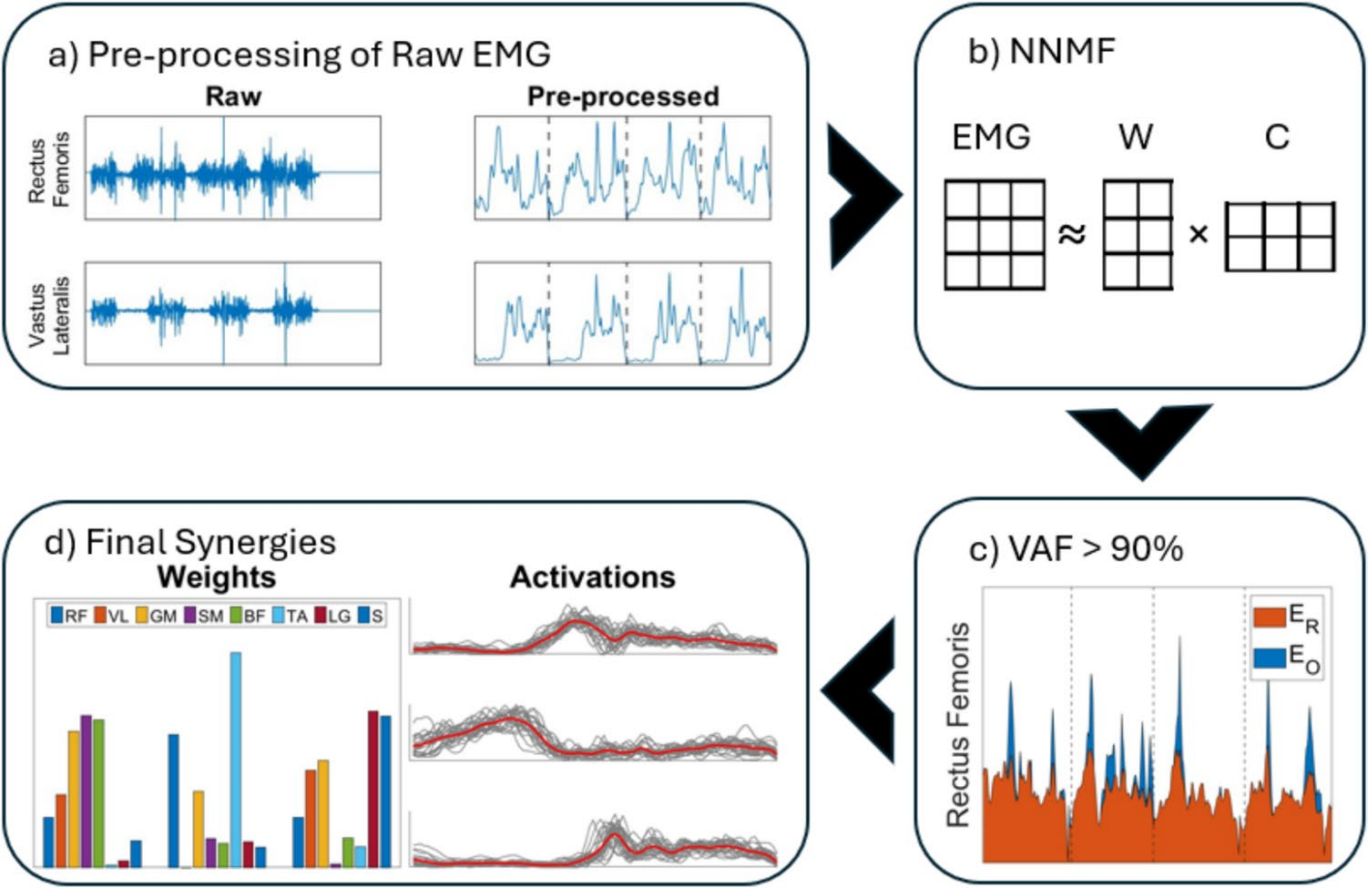
Flowchart of synergy extraction steps. **a** For each patient, raw EMG of each muscle from all gait trials was collated and pre-processed. **b** The pre-processed EMG was decomposed into muscle synergies (***W***) and their activation coefficients (**C**) using NNMF. **c** In the *VAF Method*, ${VAF}_{{n}_{syn}}$ was then calculated for 1–8 synergies to find the lowest number of synergies, ${n}_{syn}$, where ${VAF}_{{n}_{syn}}>$ 90%. Plot showing example of original muscle activity (${E}_{O}$) and reconstructed muscle activity (${E}_{R}$) that were used to calculate ${VAF}_{{n}_{syn}}$. **d** Final muscle synergies and their corresponding activations were then obtained

All sEMG data were analysed independently for each leg using custom-built functions in MATLAB (R2022b, The MathWorks Inc. Natick, MA, USA). In this pre-processing step, we prepared the raw sEMG signals following the methodology described in Cheung et al. (2005, 2020)*.* Raw sEMG signals were first high-pass filtered (Finite Impulse Response [FIR] filter, cutoff frequency of 50 Hz), rectified, and low-pass filtered (FIR filter, cutoff frequency of 20 Hz). The signals were then integrated at 10 ms intervals to approximately match the sampling frequency of the kinematic data while preserving the low-frequency temporal structure relevant to gait-related synergy analysis. Occasional high-amplitude spikes due to motion artifact or noise were removed using the de-spiking method outlined in Lin et al. (2025)*.* Additional synergy analysis without the de-spiking step is provided in Online Resource 3b, to demonstrate that the de-spiking methodology did not impact the main conclusions of this study. The signals were then normalized to unit variance (Cheung et al. 2009) (Fig. 1a). The processed, normalized signals, henceforth referred to as sEMG envelopes, were resampled such that they consisted of 100 data points for each stride.

### Synergy extraction

We used non-negative matrix factorisation algorithm (Lee and Seung 1999) to extract muscle synergies from the sEMG envelopes of each patient (Fig. 1b). This algorithm decomposes the sEMG into a linear combination of time-invariant muscle synergy weights W (m × ${n}_{syn}$ matrix: m = number of muscles, ${n}_{syn}$= number of synergies), and their corresponding time-variant activation coefficients *C(t)* (${n}_{syn}$ × t matrix: ${n}_{syn}$ = number of synergies, t = number of gait cycles × 100 data points), such that:

1 $$E_{R} \approx WC$$

where, ${{\boldsymbol{E}}}_{R}$ is the reconstructed muscle activity. Each column of the **W** matrix is a synergy vector ${w}_{j}$ (j = columns 1 to ${n}_{syn}$) that denotes the weighting (0–1) attributed to the ${i}^{th}$ ($i$ = rows, 1 to m muscles) muscle. For each patient, we analysed the muscle activations of each leg separately. For hemiplegic patients (P6 and P10), only the affected side was analysed. Following the methodology used in Shuman et al. (2019)*,* EMG envelopes from multiple gait cycles were concatenated to improve synergy estimates (Oliveira et al. 2014). Previous literature has identified changes in muscle synergies due to variations in the number of gait cycles used for extraction between assessments (Chujo et al. 2022; Sorek et al. 2022). To ensure robustness in synergy extraction methods, two additional synergy extractions with the same number of gait cycles pre- and post-surgery were performed where the included gait cycles were varied as much as possible. Similarity between these additional extractions and the original synergy weights was then assessed using Pearson’s correlation coefficient.

To determine the number of muscle synergies ${n}_{syn}$, variance accounted for (VAF) by ${n}_{syn}$ synergies was calculated as:

2 $$\begin{array}{*{20}c} {VAF_{{n_{syn} }} = \left( {1 - \frac{{\mathop \sum \nolimits_{ij} \left( {E_{O} - E_{R} } \right)^{2} }}{{\mathop \sum \nolimits_{ij} \left( {E_{O} } \right)^{2} }}} \right) \times 100\% } \\ \end{array}$$

where ${E}_{O}$ was the recorded muscle activity and the symbol ${\Sigma }_{ij}$ denotes the sum of all elements. The number of synergies was determined by selecting the lowest ${n}_{syn}$ that had a ${VAF}_{{n}_{syn}}$ $>$ 90% (Shuman et al. 2019). The synergy extraction with the highest VAF within ${n}_{syn}$ was chosen for further analysis (Fig. 1c,d). This VAF-based method of extracting synergies is henceforth referred to as the *VAF Method*.

We note that in literature, some studies also use the coefficient of determination (${R}^{2}$) value as a metric to determine the number of synergies (Cheung et al. 2020; d’Avella et al. 2006; Santuz et al. 2018). To compare, we calculated the ${R}^{2}$ value as,

3 $$\begin{array}{*{20}c} {R^{2} = \left( {1 - \frac{{\mathop \sum \nolimits_{ij} \left( {E_{O} - E_{R} } \right)^{2} }}{{\mathop \sum \nolimits_{ij} \left( {E_{O} - mE} \right)^{2} }}} \right)} \\ \end{array}$$

where $mE$ is the average muscle activity of the ${i}^{th}$ muscle (Cheung et al. 2020). ${R}^{2}$ was calculated for each extraction determined by the ${VAF}_{{n}_{syn}}$ $>$ 90% criteria, and a separate synergy extraction was completed with the criteria ${R}^{2}$ $>$ 0.8 for comparison (Cheung et al. 2012). The synergy extraction with the highest ${R}^{2}$ was chosen for further analysis. This ${R}^{2}$-based method of extracting synergies is henceforth referred to as the ${R}^{2}$ *Method*. To reduce the risk of the solution reflecting a local minima, each extraction was repeated 200 times, with randomised initial seed values for both **W** and **C** (Cheung et al. 2020).

### Synergy analysis

#### Patient specific synergy pre-post correlation

Similarity of the muscle synergy weights and activations between pre- and post-surgery was assessed by computing the Pearson’s correlation coefficient ($r$) between each pre-surgery synergy and its matching post-surgery synergy. In patients where there was a change in the number of synergies pre-to-post-surgery, the unmatched synergies correlation was calculated for all opposing synergies, and the highest correlation was selected. Synergy activations were first aligned based on the muscle synergy pair, then the Pearson’s correlation coefficient was calculated in the same manner.

#### Clustered synergy pre-post correlation

After inspection of the extracted synergies, we observed that some synergies were highly similar across patients. To assess how comparable the extracted synergies were, we implemented $k$-means clustering to group similar synergies (Cheung et al. 2020). $K$-means clustering identifies $k$ groups of synergies that are similar between patients by minimising the point-to-centre sum between each synergy and its closest cluster (Arthur and Vassilvitskii 2007). The number of clusters was determined by calculating the gap statistic (Tibshirani et al. 2001). Following methodology described in Cheung et al. (2020) and Tibshirani et al. (2001)*,* reference data sets (N = 500) were created by uniformly sampling over the range of all the patients synergies and were clustered using $k$-means (100 replicates) at 1–51 clusters pre-surgery, and 1–50 clusters post-surgery where 51 and 50 were the total number of extracted synergies for the whole cohort pre-surgery and post-surgery, respectively. The optimal number of clusters ($k$) was determined when,

4 $$\begin{array}{*{20}c} {Gap\left( k \right) \ge Gap\left( {k + 1} \right) - sd\left( {k + 1} \right)} \\ \end{array}$$

where $Gap$ is the gap statistical operator that calculated the gap between the within-cluster dispersion of the data and the reference data sets, and $sd$ is the standard deviation of this value. We refer the reader to the detailed procedures outlined in Tibshirani et al. (2001) for the computation of this metric.

$K$-means was computed using the MATLAB Statistics Toolbox function, implemented with the squared-Euclidean metric, with clustering repeated 1000 times with random initialisations to avoid a local minima (Arthur and Vassilvitskii 2007). The replicate with the smallest point-to-centre sum was chosen as the final solution. The scalar product between each synergy and their corresponding cluster’s mean synergy vector was calculated to isolate unmatched synergies (where the summed scalar product $<$ 0.8) (Cappellini et al. 2016). Similarity between the pre- and post-surgery clusters was assessed in the same manner as the patient specific synergy weights using Pearson’s correlation coefficient.

#### Muscle synergy sparseness

On observation of the extracted muscle synergy vectors, it appeared that some patients’ muscle synergies had fewer muscle components that were higher weighted (suggesting more isolated muscle recruitment) than others. To quantify this, we calculated the sparseness ($S$) of each muscle synergy as:

5 $$\begin{array}{*{20}c} {S\left( w \right) = \frac{{\sqrt m - \frac{{\mathop \sum \nolimits_{i = 1}^{m} \left| {w_{i} } \right|}}{{\sqrt {\mathop \sum \nolimits_{i = 1}^{m} w_{i}^{2} } }}}}{\sqrt m - 1}} \\ \end{array}$$

where $m$ is the number of muscles within the muscle synergy weights ${\boldsymbol{w}}$ (Hoyer 2004). A non-sparse muscle synergy vector where all elements are equal should have a sparseness of 0, while an extremely sparse muscle synergy vector should have a sparseness of 1. For each participant, we calculated the average sparseness of all muscle synergies for each leg (excluding the nonaffected side of the hemiplegic patients).

## Results

### Number of synergies, and influence of synergy extraction method

Variance in the number of gait cycles available for each assessment had minimal impact on the resulting synergy structures (Online Resource 3c). While 4 of the 20 analyses showed sensitivity to the included gait cycles, they still demonstrated high similarity when all gait cycles were concatenated. As such, to ensure results were not impacted by this sensitivity and instability, all available gait cycles were used for all patients. On average, ~ 27 gait cycles were utilised for the synergy extraction, where 1–4 muscle synergies were extracted for all patients using the *VAF method* (Table 2). Out of the 20 individual leg analyses, 11 (55%) preserved the same number of synergies pre-to-post, 5 analyses (25%) decreased by one, and 4 analyses (20%) increased by one. For the *VAF Method*, the mean ${R}^{2}$ metric for synergy extractions was 0.68 $\pm$ 0.09.

**Table 2:** Synergy extraction results based on the *VAF Method*

Patient		No. of gait cycles		VAF (%)		${R}^{2}$(*VAF method*)		No. of muscle synergies (*VAF method*)	
		Pre	Post	Pre	Post	Pre	Post	Pre	Post
P1	Right	26	17	93.29	92.96	0.73	0.64	3	2
	Left	35	30	92.87	92.20	0.71	0.60	3	2
P2	Right	27	25	93.58	92.83	0.73	0.66	2	2
	Left	21	30	90.09	93.44	0.59	0.67	1	2
P3	Right	8	23	91.16	93.68	0.63	0.80	2	3
	Left	7	30	93.35	93.24	0.77	0.80	2	3
P4	Right	16	17	93.46	91.54	0.78	0.78	3	4
	Left	33	22	91.61	92.68	0.75	0.76	4	4
P5	Right	18	13	92.66	92.21	0.73	0.74	3	3
	Left	18	17	92.01	90.51	0.80	0.60	3	3
P6	Right	22	35	92.75	94.50	0.80	0.75	4	3
P7	Right	9	48	91.67	93.13	0.64	0.45	2	1
	Left	19	44	90.20	93.58	0.69	0.56	2	2
P8	Right	15	33	93.24	92.03	0.67	0.58	3	2
	Left	17	32	93.23	94.01	0.71	0.75	3	3
P9	Right	49	25	92.63	91.97	0.75	0.71	3	3
	Left	64	36	90.01	91.61	0.71	0.67	2	2
P10	Right	31	53	92.63	92.10	0.68	0.69	2	2
P11	Right	38	24	91.09	91.72	0.60	0.54	2	2
	Left	33	26	92.42	91.75	0.58	0.54	2	2

P6 and P10 were diagnosed with hemiplegia and only had their affected limb analysed

For comparison, using the ${R}^{2}$ *Method*, we extracted between 3 and 5 muscle synergies for all patients, pre- and post-surgery (results presented in Online Resource 1). Similar to the *VAF method*, 12 analyses (65%) preserved the same number of synergies pre-to-post, 4 analyses (20%) decreased by one, and 4 analyses (20%) increased by one. For the ${R}^{2}$ *Method*, the mean VAF for synergy extractions was 95.11 $\pm$ 1.19%.

Three patients were randomly selected for a trial comparison of the *VAF* and ${R}^{2}$ *Methods* to assess whether the correlation of pre- and post-surgery synergies differed between methods. From this comparison, we found that there was a slight increase in correlation when determining the synergies by the ${R}^{2}$ *Method* (0.15 $\pm$ 0.11). As this change was minimal, we concluded that it was sufficient to proceed using only the *VAF Method* as this was the more common method applied in other CP studies (Goudriaan et al. 2018; Kim et al. 2018; Shuman et al. 2016; Steele et al. 2015).

### Patient specific synergy pre-post correlation

**Fig. 2:**
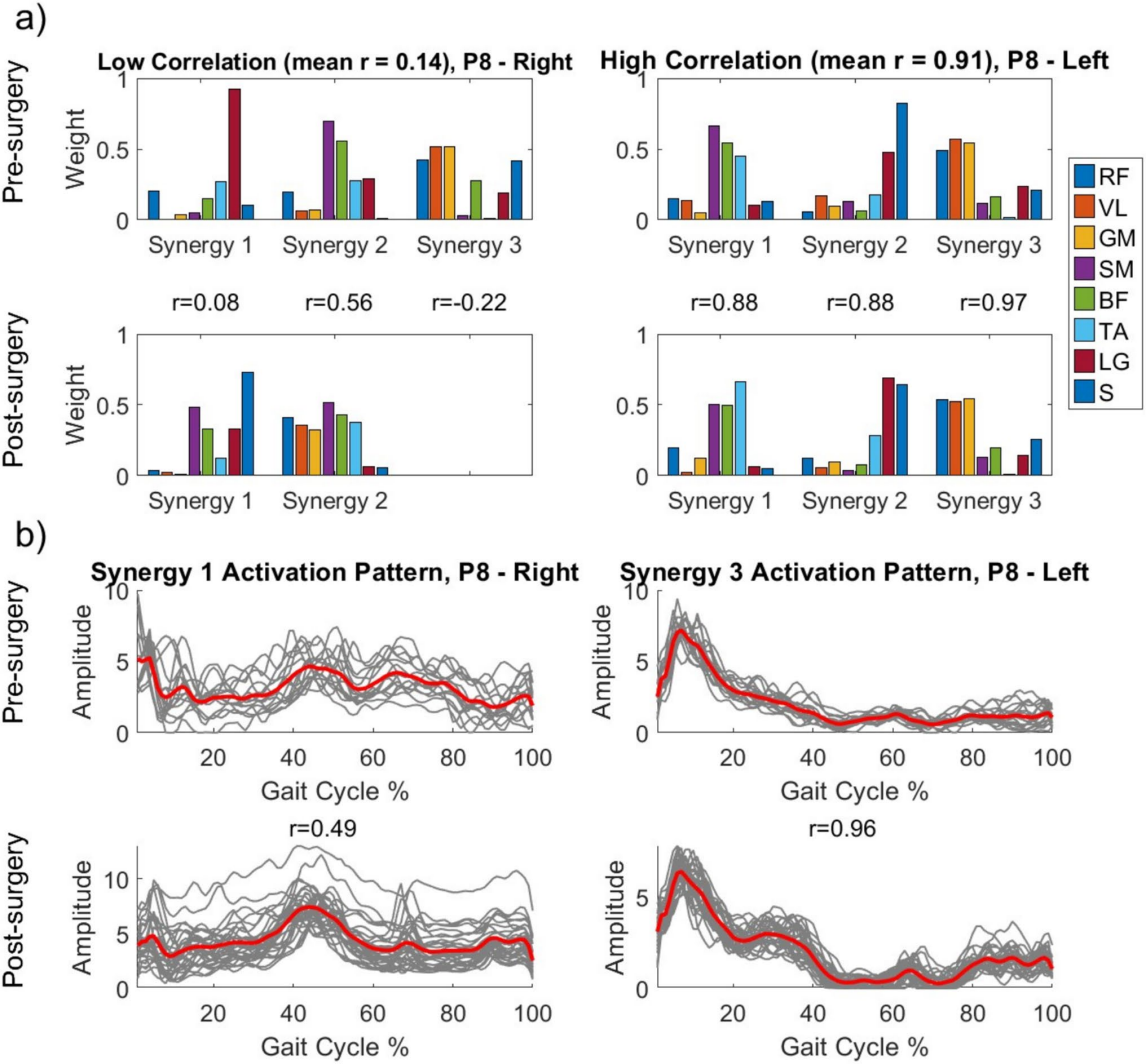
Patient specific correlation demonstrating examples of low and high correlation between pre- and post-surgery muscle synergies in P8. **a** Preservation of synergy weights can be seen in the high correlation plots. Pearson’s correlation coefficient ($r$) is displayed for each synergy pair. For the unmatched synergy, $r$ was calculated for both post-surgery synergies, and the highest value is displayed. **b** Representative activation patterns for P8 with Synergy 1 (from low correlation plot in Panel (a)) and Synergy 3 (from high correlation plot in Panel (a)). Thin shaded lines indicate trial data over the gait cycle, and the solid line the mean over trials

**Fig. 3:**
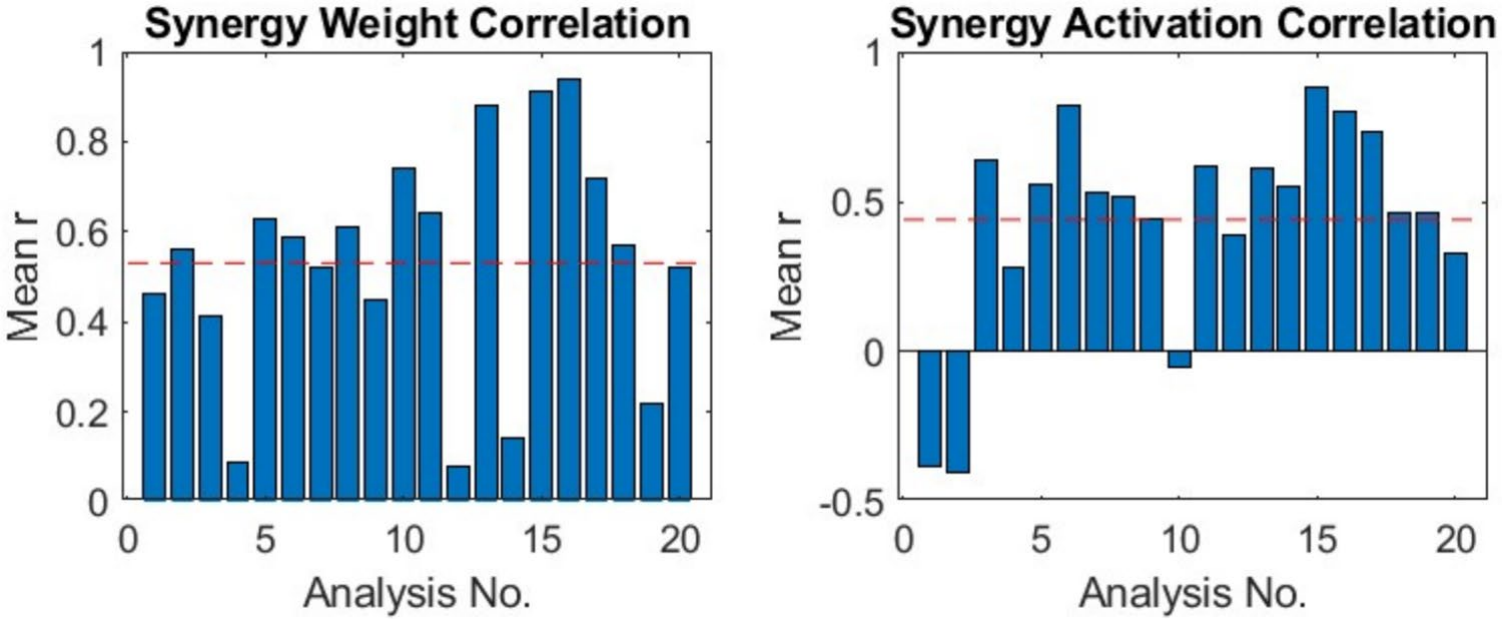
Mean correlation between pre- and post-surgery of synergy weights and activations for all analyses with VAF $>$ 90%

While there were some similarities between pre- and post-surgery synergies at a patient specific level, they were not highly similar across the cohort (Table 3). To illustrate these changes, we contrast the analyses results from P8-Right (as an example of low correlation) and P8-Left (as an example of high correlation) (Fig. 2). For P8-Right, we observed that we had one unmatched synergy (synergy 3) between pre- and post-surgery, with a resulting synergy weight correlation of 0.14. The corresponding behaviour of the synergy activation profile over the gait cycle for P8-Right’s synergy 1 (left panels on Fig. 2b) shows poor preservation of the temporal pattern. In contrast, synergy weights for P8-Left were highly correlated and the synergy activations shows a clear observable preservation of the temporal pattern. Out of the 46 paired synergies, 19 (41%) were highly similar ($r$ $>$ 0.8) pre-to-post. However, Pearson’s correlation coefficient of the synergy weights revealed an overall mean correlation of 0.53 $\pm$ 0.25, whilst mean correlation of the activations was found to be 0.44 $\pm$ 0.35 (Fig. 3).

**Table 3:** Synergy analysis results

Patient		No. of similar synergies	Synergy weight correlation	Synergy activation correlation	No. of unmatched synergies		VAF of cohort synergies (%)		Mean sparseness	
					Pre	Post	Pre	Post	Pre	Post
P1	Right	0	0.46	− 0.38	0	1	87.02	84.87	0.37	0.24
	Left	1	0.56	− 0.40	1	1	89.99	88.84	0.40	0.29
P2	Right	0	0.41	0.64	0	1	83.66	83.63	0.23	0.25
	Left	0	0.09	0.28	1	2	–	–	0.02	0.28
P3	Right	1	0.63	0.56	0	0	84.62	87.39	0.26	0.38
	Left	1	0.59	0.82	0	1	88.85	84.16	0.28	0.46
P4	Right	1	0.52	0.53	1	4	87.76	–	0.47	0.55
	Left	2	0.61	0.52	2	3	88.62	90.27	0.60	0.49
P5	Right	0	0.45	0.44	1	2	88.37	89.83	0.48	0.41
	Left	1	0.74	− 0.05	0	0	89.12	85.38	0.42	0.38
P6	Right	2	0.64	0.62	2	2	87.76	92.86	0.44	0.41
P7	Right	0	0.08	0.39	0	0	87.88	76.39	0.21	0.05
	Left	2	0.88	0.61	0	1	85.23	86.37	0.29	0.21
P8	Right	0	0.14	0.55	2	1	90.83	89.50	0.40	0.29
	Left	3	0.91	0.88	2	2	90.72	90.24	0.35	0.37
P9	Right	3	0.94	0.80	0	0	88.42	84.58	0.37	0.37
	Left	1	0.72	0.73	1	0	88.53	85.06	0.24	0.28
P10	Right	1	0.57	0.46	1	0	88.79	86.70	0.26	0.26
P11	Right	0	0.22	0.46	0	0	86.28	83.85	0.29	0.27
	Left	0	0.52	0.33	1	1	84.80	87.02	0.24	0.21

P6 and P10 were diagnosed with hemiplegia and only had their affected limb analysed. VAF of Cohort Synergies was not calculated for patients without any matched synergies in the cohort analysis

### Clustered synergy pre-post correlation

**Fig. 4:**
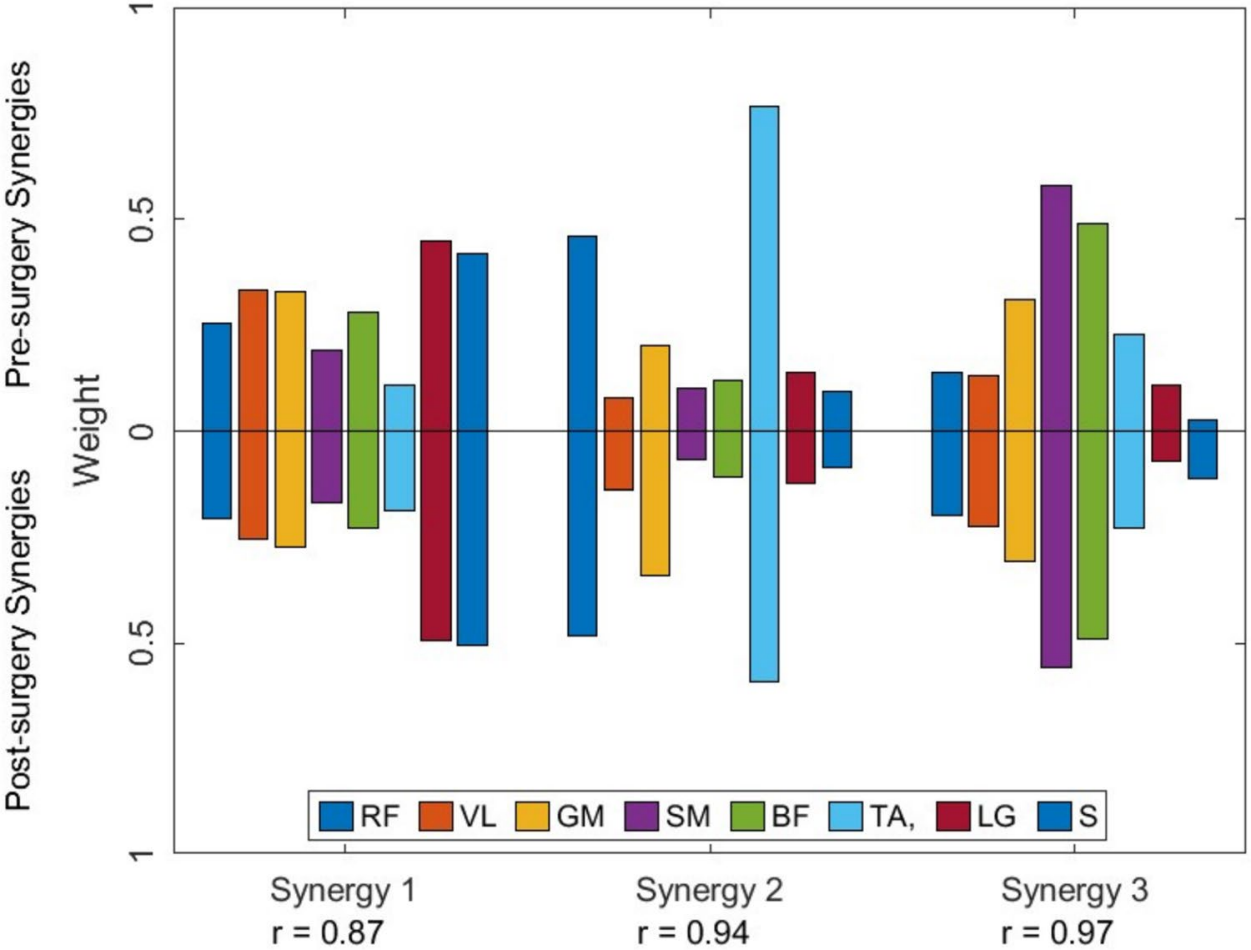
Pre- and post-surgery cohort synergy clusters and corresponding Pearson’s correlation coefficient

Application of the clustered synergy correlation method revealed that 3 clusters (as determined via the gap statistic) explained 70% and 56% of the pre-surgery and post-surgery synergies, respectively. There were 15 synergies pre-surgery and 22 synergies post-surgery that were unmatched. Replacing matched synergies with their corresponding mean cluster vector (henceforth referred to as cohort synergies), resulted in a slightly reduced VAF (87.14 $\pm$ 2.96%). Correlation between the mean synergy vectors obtained through clustering for pre- and post-surgery demonstrated all 3 synergies were highly similar ($r$ $>$ 0.87) (Fig. 4). The presence or absence of any cohort synergy did not explain changes at a patient specific level. While post-surgery cohort synergy 2 only occurred in patients with a mean synergy weight above the cohort average ($r$ $>$ 0.53), at a patient specific level it was not always highly similar. Additionally, whilst post-surgery cohort synergy 2 never occurred without pre-surgery cohort synergy 2, the pre-surgery synergy was not always preserved, and some patients had the pre-surgery synergy and not the post-surgery synergy. All other cohort synergies and unmatched synergies showed both high and low correlation when assessed at a patient specific level and were shared among patients both above and below the mean values for synergy weights and activations.

### Muscle synergy sparseness

**Fig. 5:**
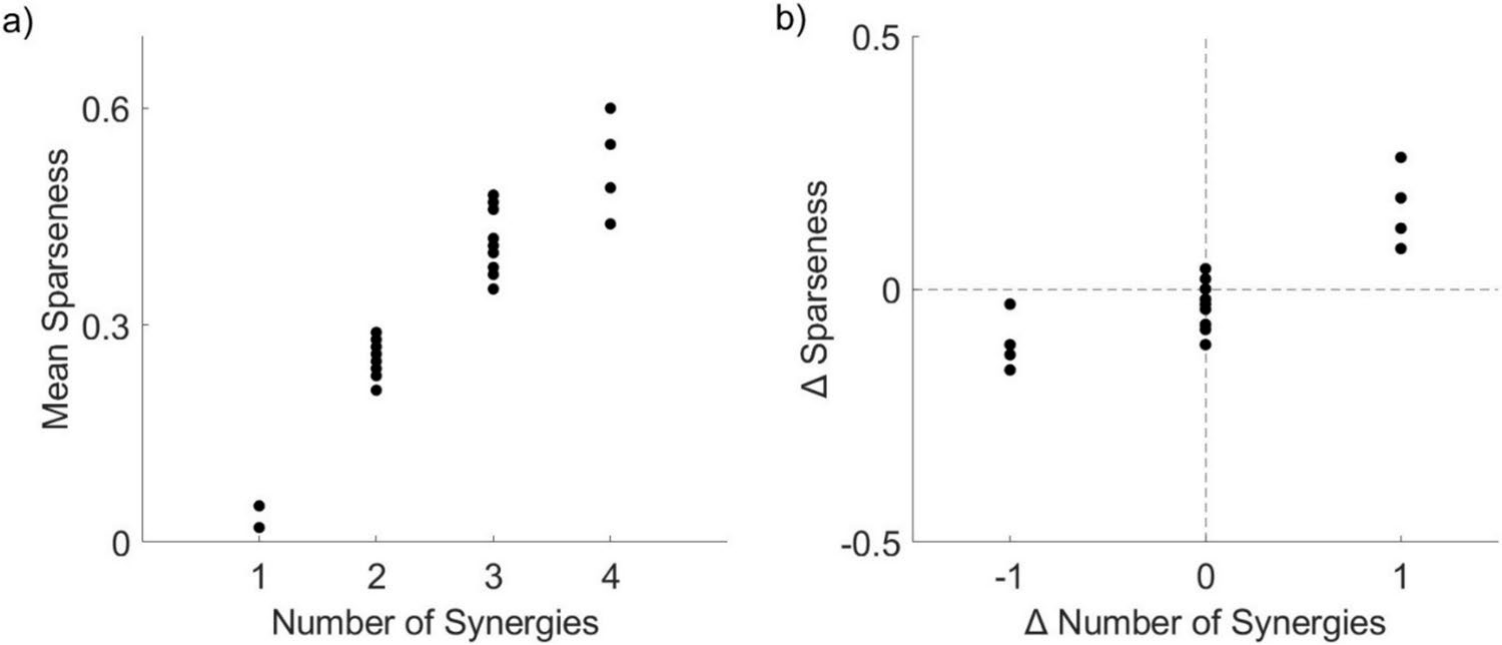
Muscle synergy sparseness analysis. **a** Average sparseness increased with the number of synergies. **b** Change in sparseness corresponding to change in number of synergies, between pre- and post-surgery

We identified that at a patient specific level sparseness could change between pre- and post-surgery, with patients exhibiting either an increase or a decrease in sparseness (Table 3). Additionally, we observed that sparseness increased with the increase in the number of synergies (Fig. 5a). For patients that exhibited a change in the number of synergies from pre- to post-surgery, we observed the corresponding change in the sparseness of their synergies (i.e. patients that had an increase in the number of synergies also had an increase in sparseness) (Fig. 5b). Patients that preserved the same number of synergies between pre- and post-surgery analyses could demonstrate either an increase or decrease in sparseness.

### Sparseness and changes in gait

**Fig. 6:**
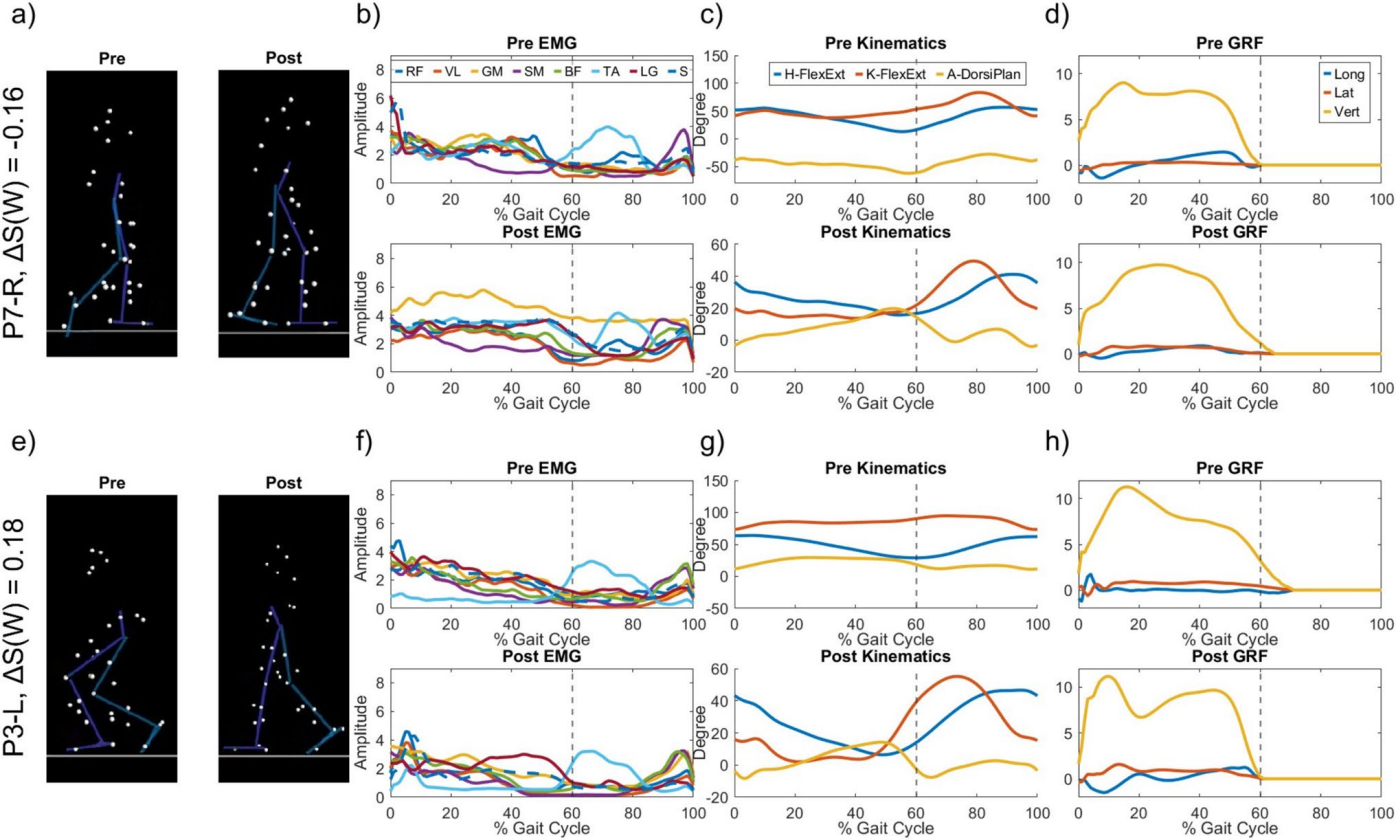
Comparison of two patients with greatest changes in sparseness. **a** Snapshot of pre- and post-surgery gait pattern of P7-R at 60% of the gait cycle. **b** Mean EMG of P7-R pre- and post-surgery. **c** Sagittal plane kinematics of P7-R pre- and post-surgery where H-FlexExt and K-FlexExt are the flexion and extension angles of the hip and knee respectively, while A-DorsiPlan is the ankle dorsiflexion and plantar flexion angle **d** Plot of ground reaction forces of P7-R pre- and post-surgery including Longitudinal (Long), Lateral (Lat), and Vertical (Vert). **e** Model of pre- and post-surgery gait pattern of P3-L at 60% of the gait cycle. **f** Mean EMG of P3-L pre- and post-surgery. **g** Sagittal plane kinematics of P3-L pre- and post-surgery.** h** Plot of ground reaction forces of P3-L pre- and post-surgery including Longitudinal (Long), Lateral (Lat), and Vertical (Vert)

To illustrate the effects of sparseness change in motor control on the patient’s gait, we showcase the sEMG envelopes, sagittal plane joint kinematics, and ground reaction forces (GRF) of two patients from our cohort (Fig. 6). These two cases were at the extremes of sparseness change pre- to post-surgery, with P3-L exhibiting a sparseness increase of $\Delta S(W)=0.18$, and, P7-R a sparseness decrease of $\Delta S(W)=-0.16$. Note that while P2-L had a greater increase in sparseness than P3-L (Table 3), this patient used a walking aid pre-surgery and subsequently GRF data were not available. The two cases also presented with different clinical problems. P3 had undergone previous SEMLS and presented with a predominantly orthopaedic problem with some minor spasticity (good motor control), whilst P7 had no previous surgeries, no strong contractures, but strong spasticity at all levels (poor motor control). We observed that even though P7-R shows improvements in the sagittal plane angles of the knee and ankle, these changes were not very pronounced. Additionally, their post-surgery vertical GRF profile shows an absence of the mid-foot transition that was observed in the pre-surgical assessment. On the other hand, P3-L showed improvement in all post-surgery gait kinematics with their joint angles more closely approaching the nominal range found in typically developing children (Tsitlakidis et al. 2022). Their vertical GRF profile also shows a clear mid-foot transition and a stereotypical shape. Similar observations are available for all patients in Online Resource 3d.

## Discussion

In this study, we investigated and quantified the changes in muscle synergies after SEMLS in children with CP. Due to the complex and highly individualistic nature of CP, clinical presentation, treatment strategies and sequence of interventions can differ between participants. While this heterogeneity limits generalisation, the cohort in our study reflects a realistically diverse clinical population. Contrary to our hypothesis, at the patient specific level, muscle synergy weights and activations were not highly similar between pre-surgery and post-surgery assessments. Although synergies obtained at a cohort level were highly similar between assessments ($r$ $>$ 0.87), neither the presence nor absence of these at the pre-surgery assessment were an indication for highly similar motor control post-surgery at a patient specific level. These findings suggest that patient specific pre-surgery motor control is not always preserved post-surgery for all CP patients.

### Comparison of VAF and R^2^ methods

Through the comparison of two methods commonly used to determine the minimum number of synergies, *VAF* (Oudenhoven et al. 2019; Shuman et al. 2019; Torricelli et al. 2014) and ${R}^{2}$ (Cheung et al. 2020; d’Avella et al. 2006; Santuz et al. 2018), we found there was minimal impact on the analysis of results, regardless of method choice. Previous research has reported children with CP commonly presenting with 1–4 synergies during gait (Bekius et al. 2020), aligning with our results from the *VAF Method*. While the ${R}^{2}$ *Method* required more synergies to fulfil the minimum criteria, this increase is likely due to this method resulting in a higher VAF of 95%, when the commonly used cutoff is 90% in other CP studies (Oudenhoven et al. 2019; Shuman et al. 2019; Torricelli et al. 2014). Despite this, when calculating the correlation between the pre- and post-surgery synergies, minimal differences were observed between the two methods.

### Synergy similarity at a patient specific level

While previous research has analysed muscle synergies in CP patients pre- and post-surgery (Pitto et al. 2020; Shuman et al. 2019), to our knowledge the change in composition of the synergies due to the surgical intervention has not been previously reported. In this study, we found 55% of analyses preserved the same number of synergies between pre- and post-surgery assessments. These results match those from Shuman et al. (2019) that reported 49% of individuals maintaining the same number of synergies after SEMLS. Studies have shown that SEMLS can result in an improved gait efficiency and a reduction of compensatory mechanisms (Borghi et al. 2025). A decrease in the number of synergies could be explained by improved gait efficiency (Cheung et al. 2020), whereas an increase is likely due to greater selective motor control (Goudriaan et al. 2022) after orthopaedic limitations have been corrected. While studies have demonstrated that the number of synergies increases with age under normal motor development (Cheung et al. 2020; Sylos-Labini et al. 2020), as the time between assessments in our cohort was relatively short (1 ~ 1.5 years, Table 1), it’s likely that the observed changes reflects the effect of the surgical intervention on patient motor control.

Our analysis also revealed that the Pearson’s correlation coefficient between the pre- and post-surgery muscle synergy weights and activations (means of 0.53 and 0.44, respectively), suggest that patient specific muscle synergies were not highly similar after surgery. This finding is in direct contrast of previous work by Shuman et al. (2019) and Pitto et al. (2020) that suggest that motor control is fixed in CP patients (GMFCS levels: I-III (Shuman et al. 2019) and I-II (Pitto et al. 2020)) with little to no changes between pre- and post-surgery synergies. The differences in our results can be explained by different comparison methods. In our study, we compared the estimated pre-surgery muscle synergies with the same patient’s post-surgery synergies, whereas Shuman et al. (2019) compared pre- and post-surgery synergies to typically developing children’s synergies, and Pitto et al. (2020) used optimisation methods to increase VAF. Neither study directly compared the estimated synergies from pre- and post-surgery to assess the composition change after surgery as has been conducted here.

Analysis of the synergy activation profiles over the gait cycle (Figs. 2b, 3), also suggest that the activation profiles showed greater changes after surgery when compared to the synergy weights. We note that the data presented in Fig. 2b are representative of low and high correlation in activation profiles, with similar observations for all analyses available in the supplementary data (Online Resource 3e). These findings are in line with results from Patikas et al. (2007), where changes in EMG patterns were reported, on a patient-specific basis, after surgical intervention in children with CP. Similar results have also been found in other cohorts, for example the study by Routson et al. (2013) investigated muscle synergies and activation profiles in stroke patients before and after gait training. They report changes in activation profiles after gait training being associated with improvements in kinematics, speed, and symmetrical propulsion.

### Synergy similarity at a cohort level

Our results indicate that at the cohort level CP patients share similar muscle synergy patterns, as assessed via cluster analysis which revealed that 70% of the pre-surgery, and 56% post-surgery muscle synergies could be explained by 3 synergies. Using similar methodology conceptions to those applied in Berger et al. (2020)*,* we calculated EMG reconstruction error to assess synergy similarity across patients. Specifically, we replaced the patient specific muscle synergies with the cohort synergies obtained via cluster analysis, and recalculated VAF. Similar to previous findings (Pitto et al. 2020), we observed only a slight decrease in VAF. High correlation was found when calculating the Pearson’s correlation coefficient of the pre- and post-surgery cohort synergies, suggesting minimal changes after SEMLS at a cohort level, and supporting the notion of using cohort synergies in predictive simulation as proposed by Pitto et al. (2020)*.* However, the occurrence of more unmatched synergies in the post-surgery clustering is potentially an indication of more individualised motor control patterns after surgical intervention. The impact of this on the accuracy of simulations would be an interesting future research direction.

### Changes in synergy sparseness

Muscle synergy sparseness increased with the number of synergies, indicating that patients with more muscle synergies were activating muscles with greater isolation than those with fewer muscle synergies. This is comparable to results from Cheung et al. (2020) that reported similar findings comparing running synergies of preschoolers to those of sedentary adults, where the adults had more synergies and sparser synergies than the preschoolers. Comparing sparseness in pre- and post-surgery assessments revealed a concomitant increase in sparseness and number of synergies. Furthermore, our observations of P7 and P3 suggest that while the surgical intervention improved the gait of both patients, an increase in the number of synergies and sparseness after surgery could be a reasonable indication of more complex motor control and a more typical gait pattern. This observation supports the theory that patients presenting with good motor control had better treatment outcomes (Schwartz et al. 2016).

### Study limitations

The conclusions of this study are based on the observations from a relatively small cohort of patients and was completed retrospectively to analyse patient motor control after intervention. Thus, neither the individual gait pattern nor the individual intervention has been addressed in this work. Additionally, as this study lacks a control group, potential changes caused by participant growth and development could not be isolated from the effects of surgery. We also acknowledge that the cohort were identified as having varied GMFCS levels and underwent different surgeries, with sometimes the same patient having undergone different surgical interventions to the left and right legs. Although this variability and range of surgical interventions are not uncommon in CP and reflects a true representation of clinical data, it does limit our ability to assess the impact of types of surgery on motor control. While this fits within our study intention to provide an overview of a CP population, it also offers an avenue for future work with a larger cohort filtered by similar surgical interventions, clinical presentation, or other sub-groups. The assessment of more focused and homogeneous sub-groups has the potential to provide additional insight and alternative observations. These could reveal key differences between the patients that have similar muscle synergies post-surgery, and those who do not. One possible way to achieve this with a larger set of patients and surgeries is the application of multiple linear regression (similar to the approach by Oudenhoven et al. (2019)) to better understand the contributing variables. Extending this analysis to evaluate the ability to predict other changes, including the number of synergies and amount of sparseness change, can also provide valuable insight into understanding how the muscle synergy composition changes after surgical intervention. Furthermore, by separating phasic and tonic components from EMG as has been completed in Brambilla et al. (2023)*,* future studies can assess whether both movement and postural synergies are impacted by SEMLS. A known limitation of gait analysis is the sensitivity to marker placement and soft-tissue artifact. To investigate the change of SEMLS, a substantial time interval between assessments is necessary but increases the risk for error. To minimise such risk, rigorous protocol for marker and EMG placement was followed for each assessment. In this study, we have opted to include a de-spiking step to remove occasional high-amplitude spikes caused by motion artifact or noise, during sEMG pre-processing. While our additional analysis revealed that the conclusions of our study were not impacted by this step, we acknowledge that the utilised method was not necessarily developed for EMG-based muscle synergy analysis. As such, we would advise the use of similar verification steps, as applied in this study, to ensure synergy results are not impacted. Additionally, to improve muscle synergy estimates, all available gait cycles were concatenated for each patient and each side. However, this resulted in a different number of gait cycles being used in each analysis as each patient had varying amounts of data available. While the impact on the synergy structures was minimal in this study, caution should still be taken when comparing synergies with such data discrepancies. Finally, unlike previous studies that have pre-determined the number of synergies (Booth et al. 2019; Shuman et al. 2019), this study included unmatched synergies in similarity calculations to thoroughly demonstrate the changes in motor control after intervention. While the overall mean was typically reduced by the unmatched synergy in this study, we do note that in some cases, our methodology has the potential to increase similarity scores due to the highest correlation being selected for the unmatched synergy. In this study we observe consistent decreases in the mean weight similarities, and minimal increases in the mean activation similarities.

## Conclusion and future directions

The findings from our work revealed that motor control is affected by SEMLS in children with CP, with some children more impacted than others. Cohort synergies can explain sEMG reasonably well and are highly similar pre- and post-surgery. Sparseness increased with the number of synergies, however the cause of the change in number of synergies observed was unclear. Future work in this area should focus on determining the causes of the changes this study has identified in muscle synergies after SEMLS. Through a better understanding of these changes, post-treatment results and motor control can be better predicted and reflected in methods to improve patient outcomes.

## Supplementary Information

Below is the link to the electronic supplementary material.

Supplementary file1 (XLSX 11 kb). Results of the synergy extraction using the ${R}^{2}$ Method. Data provided in Microsoft Excel format (ESM_1.xlsx).

Supplementary file2 (ZIP 5167 kb). Extracted synergy weights and time activation profiles for all study participants. Data is provided in Octave/MATLAB format (.mat files)

Supplementary file3 (PDF 21535 kb) (a) GMFCS and Surgical Details for all patients (b) Analysis results on effect of de-spiking step on sEMG processing (c) Analysis results on effect of included gait cycles on synergy extraction (d) Observations of EMG, Kinematics and GRF pre- and post-surgery for all patients (e) Patient specific correlation of synergy activations profiles for all patients

## Data Availability

Extracted synergies for all participants in this study is available as supplementary data. Raw clinical recordings are not available due to patient confidentiality limitations.
