# Supplementary material for: Influence of surgical intervention on pre- and post-surgery patient specific muscle synergies in children with cerebral palsy
Source: Exp Brain Res. 2026 May 4;244(6):107. doi: 10.1007/s00221-026-07308-8 (PMC13139245; doi:10.1007/s00221-026-07308-8)
Supplement: Supplementary file 3 — Supplementary file3 (PDF 21535 kb) (a) GMFCS and Surgical Details for all patients (b) Analysis results on effect of de-spiking step on sEMG processing (c) Analysis results on effect of included gait cycles on synergy extraction (d) Observations of EMG, Kinematics and GRF pre- and post-surgery for all patients (e) Patient specific correlation of synergy activations profiles for all patients [file 221_2026_7308_MOESM3_ESM.pdf]

Supplementary Data to the article "Influence of surgical intervention on pre- and post-surgery patient specific muscle synergies in children with cerebral palsy", by Tiana Breust, Jiayin Lin, Vincent C. K. Cheung, Firooz Salami, Sebastian I. Wolf, Gursel Alici and Manish Sreenivasa

Online Resource 3a:

Supplementary Table: GMFCS and Surgical Details for all Patients.

Patient	GMFCS		Surgeries	
	Pre	Post	Right Leg	Left Leg
P1	3	2	Complex (multidimensional) femoral osteotomy with axis correction, supramalleolar rotational osteotomy, achilles tendon lengthening<br>Patella advancement	Complex (multidimensional) femoral osteotomy with axis correction, supramalleolar rotational osteotomy, achilles tendon lengthening<br>Patella advancement
P2	3	3	Supracondylar extension osteotomy,<br>Chopartarthrodesis,<br>Wire osteosynthesis foot<br>Tibialis anterior tendon lengthening<br>Achilles tendon augmentation (Nicoladoni)<br>Patella advancement<br>Single-stage multiple interventions on four metatarsal and/or toe rays	Supracondylar extension osteotomy,<br>Chopartarthrodesis,<br>Wire osteosynthesis foot<br>Tibialis anterior tendon lengthening<br>Achilles tendon augmentation (Nicoladoni)<br>Patella advancement<br>Single-stage multiple interventions on four metatarsal and/or toe rays
P3	2	2	Supracondylar extension osteotomy<br>Intramuscular hamstrings lengthening<br>Patella advancement	Supracondylar extension osteotomy<br>Intramuscular hamstrings lengthening Patella advancement
P4	2	2	Supracondylar extension derotation osteotomy	Supracondylar extension derotation osteotomy
P5	3	3	Supracondylar derotation osteotomy<br>Intramuscular recession adductor longus<br>Patella advancement<br>split tendon transfer tibialis anterior	Intramuscular recession adductor longus
P6	1	1	Supracondylar derotation osteotomy<br>Calcaneocuboid distraction arthrodesis<br>Plantarflexion naviculo-cuneiform arthrodesis<br>Intramuscular lengthening calf muscles (Baumann)	None
P7	2	2	Supracondylar (De-)rotation osteotomy<br>Z-plasty of the Achilles tendon	Supracondylar (De-)rotation osteotomy<br>Intramuscular lengthening calf muscles (Baumann)
P8	1	1	Femoral extension and shortening osteotomy	Femoral extension and shortening osteotomy
P9	1	1	Temporary epiphyseodesis distal femur	Supracondylar derotation-extension osteotomy<br>Z-plasty Achilles tendon<br>Temporary epiphysiodesis tibia
P10	2	2	(De-)rotational osteotomy distal femur<br>(De-)rotation osteotomy, distal tibia<br>Achilles tendon lengthening	Temporary epiphysiodesis proximal tibia
P11	2	2	Complex multidimensional supracondylar osteotomy<br>Patella advancement<br>Supramalleolar derotation osteotomy<br>Intramuscular recession adductor longus muscle	Complex multidimensional supracondylar osteotomy<br>Patella advancement<br>Supramalleolar derotation osteotomy<br>Intramuscular recession adductor longus muscle

Supplementary Data to the article "Influence of surgical intervention on pre- and post-surgery patient specific muscle synergies in children with cerebral palsy", by Tiana Breust, Jiayin Lin, Vincent C. K. Cheung, Firooz Salami, Sebastian I. Wolf, Gursel Alici and Manish Sreenivasa

Online Resource 3b:

Supplementary Table: Pearson's correlation coefficient of muscle synergy weights and activations pre- and post-surgery to evaluate synergy similarity after SEMLS when a de-spiking step is not applied during sEMG pre-processing.

Patient		Weights	Activations
P1	Right	0.51	-0.36
	Left	0.53	-0.44
P2	Right	-0.01	0.15
	Left	0.12	0.27
P3	Right	0.65	0.53
	Left	0.64	0.77
P4	Right	0.50	0.59
	Left	0.61	0.54
P5	Right	0.63	0.45
	Left	0.68	0.04
P6	Right	0.49	0.59
P7	Right	0.08	0.37
	Left	0.90	0.69
P8	Right	0.14	0.15
	Left	0.91	0.88
P9	Right	0.94	0.80
	Left	0.70	0.67
P10	Right	0.55	0.47
P11	Right	0.21	0.48
	Left	0.53	0.29
Mean		0.52 ± 0.28	0.40 ± 0.35

Supplementary Data to the article "Influence of surgical intervention on pre- and post-surgery patient specific muscle synergies in children with cerebral palsy", by Tiana Breust, Jiayin Lin, Vincent C. K. Cheung, Firooz Salami, Sebastian I. Wolf, Gursel Alici and Manish Sreenivasa

Online Resource 3c:

Supplementary Table: Similarity between the additional synergy extractions where the gait cycles were matched between assessments, and the original synergies that were extracted using all available gait data.

Patient		Similarity of Extraction 1	Similarity of Extraction 2
P1	Right	1.00	1.00
	Left	0.97	0.99
P2	Right	1.00	1.00
	Left	0.99	0.98
P3	Right	0.97	1.00
	Left	0.96	0.97
P4	Right	1.00	1.00
	Left	0.99	0.99
P5	Right	1.00	0.99
	Left	1.00	1.00
P6	Right	0.51 (-1syn)	1.00
P7	Right	0.97	0.98
	Left	1.00	1.00
P8	Right	0.45 (-1syn)	0.97
	Left	0.59 (-1syn)	0.98
P9	Right	1.00	0.99
	Left	1.00	0.68 (+1syn)
P10	Right	1.00	1.00
P11	Right	0.95	0.99
	Left	0.99	0.98

Supplementary Data to the article "Influence of surgical intervention on pre- and post-surgery patient specific muscle synergies in children with cerebral palsy", by Tiana Breust, Jiayin Lin, Vincent C. K. Cheung, Firooz Salami, Sebastian I. Wolf, Gursel Alici and Manish Sreenivasa

Online Resource 3d: Changes in Gait

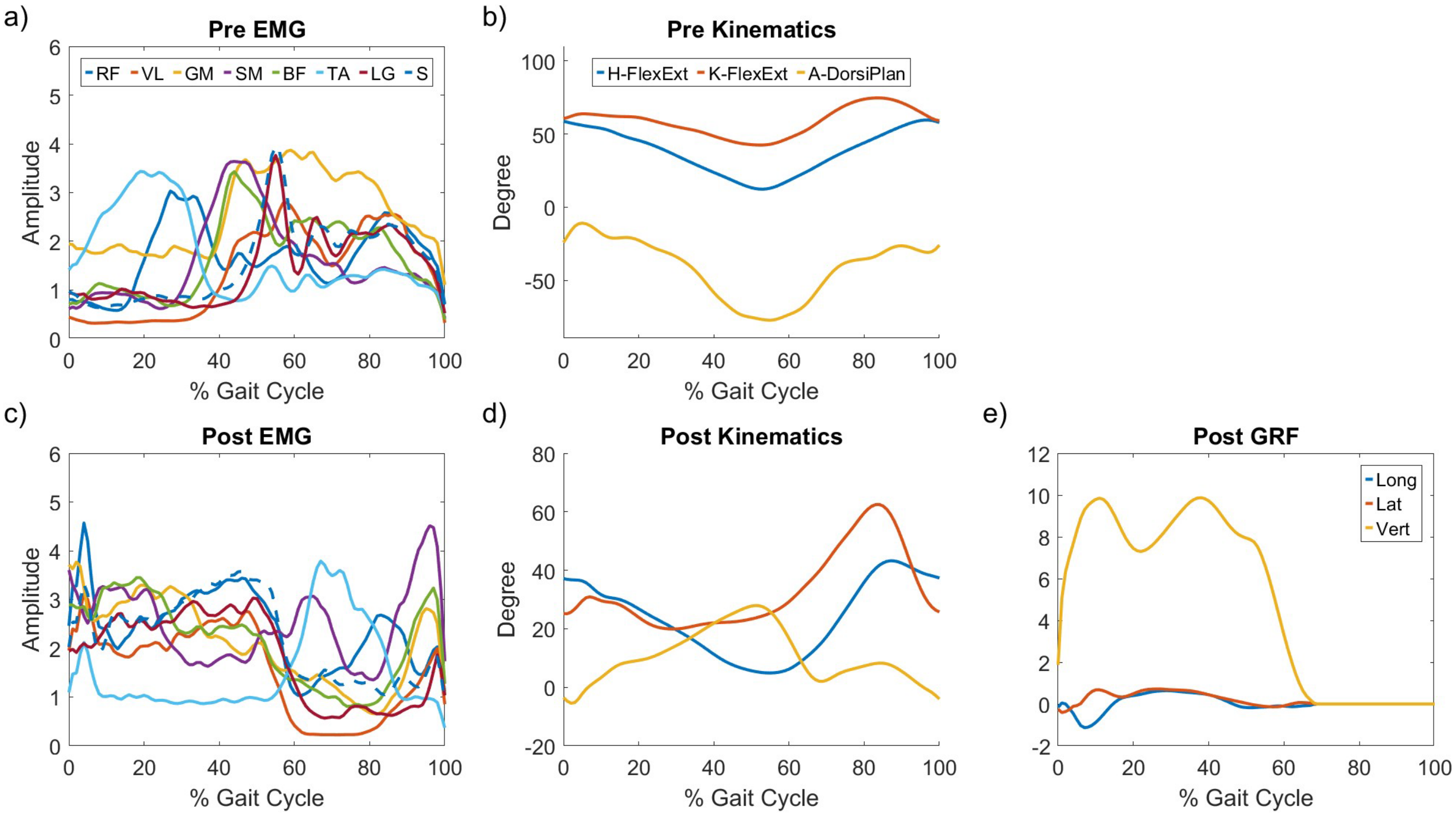

Figure 1: Observational changes between pre-surgery and post-surgery assessments for P1-Right. a) Mean pre-surgery EMG. b) Pre-surgery sagittal plane kinematics where H-FlexExt and K-FlexExt are the flexion and extension angles of the hip and knee respectively, while A-DorsiPlan is the ankle dorsiflexion and plantar flexion angle c) Mean post-surgery EMG. d) Post-surgery sagittal plane kinematics. e) Plot of post-surgery ground reaction forces including Longitudinal (Long), Lateral (Lat), and Vertical (Vert).

Supplementary Data to the article "Influence of surgical intervention on pre- and post-surgery patient specific muscle synergies in children with cerebral palsy", by Tiana Breust, Jiayin Lin, Vincent C. K. Cheung, Firooz Salami, Sebastian I. Wolf, Gursel Alici and Manish Sreenivasa

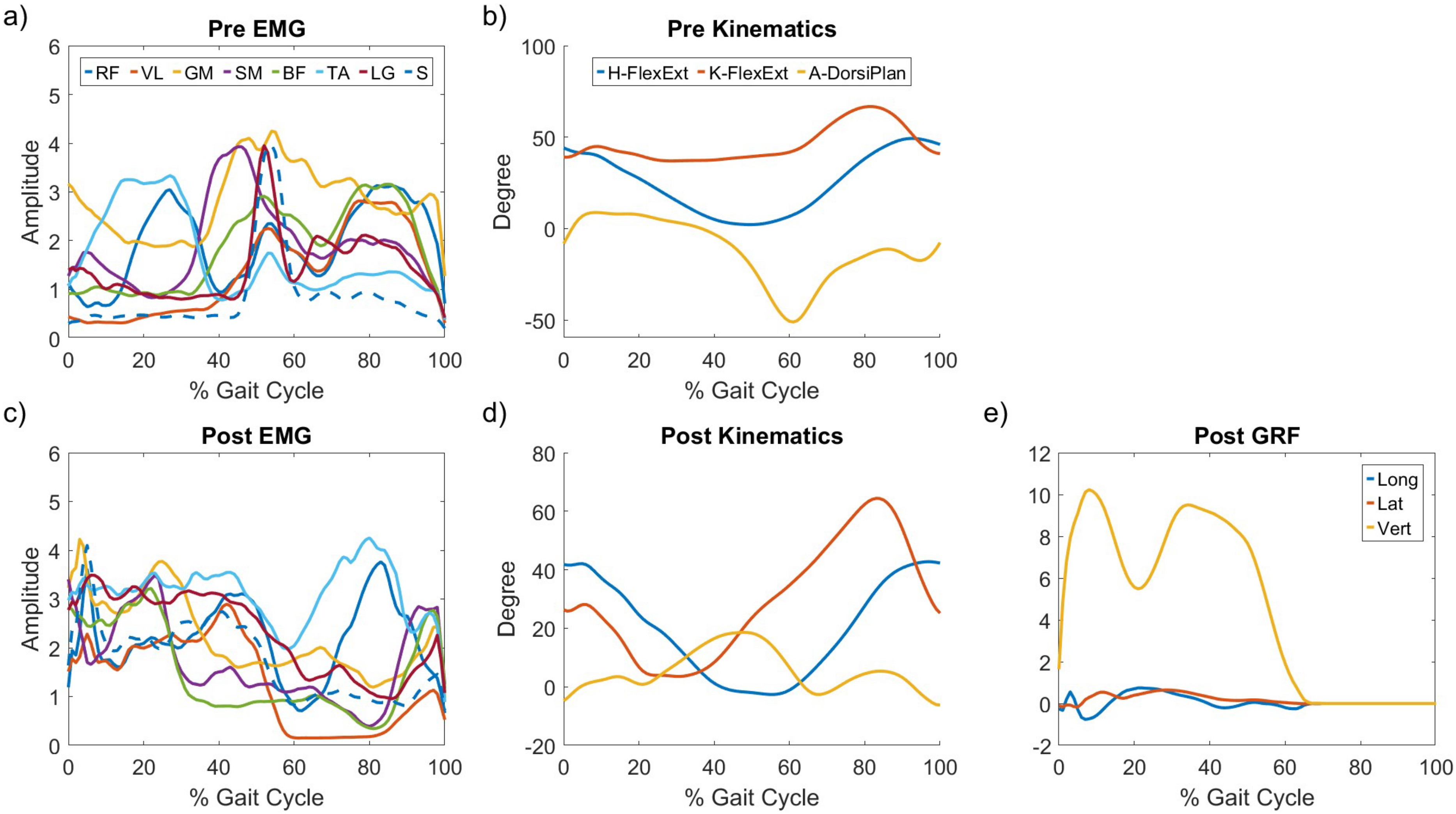

Figure 2: Observational changes between pre-surgery and post-surgery assessments for P1-Left. a) Mean pre-surgery EMG. b) Pre-surgery sagittal plane kinematics where H-FlexExt and K-FlexExt are the flexion and extension angles of the hip and knee respectively, while A-DorsiPlan is the ankle dorsiflexion and plantar flexion angle c) Mean post-surgery EMG. d) Post-surgery sagittal plane kinematics. e) Plot of post-surgery ground reaction forces including Longitudinal (Long), Lateral (Lat), and Vertical (Vert).

Supplementary Data to the article "Influence of surgical intervention on pre- and post-surgery patient specific muscle synergies in children with cerebral palsy", by Tiana Breust, Jiayin Lin, Vincent C. K. Cheung, Firooz Salami, Sebastian I. Wolf, Gursel Alici and Manish Sreenivasa

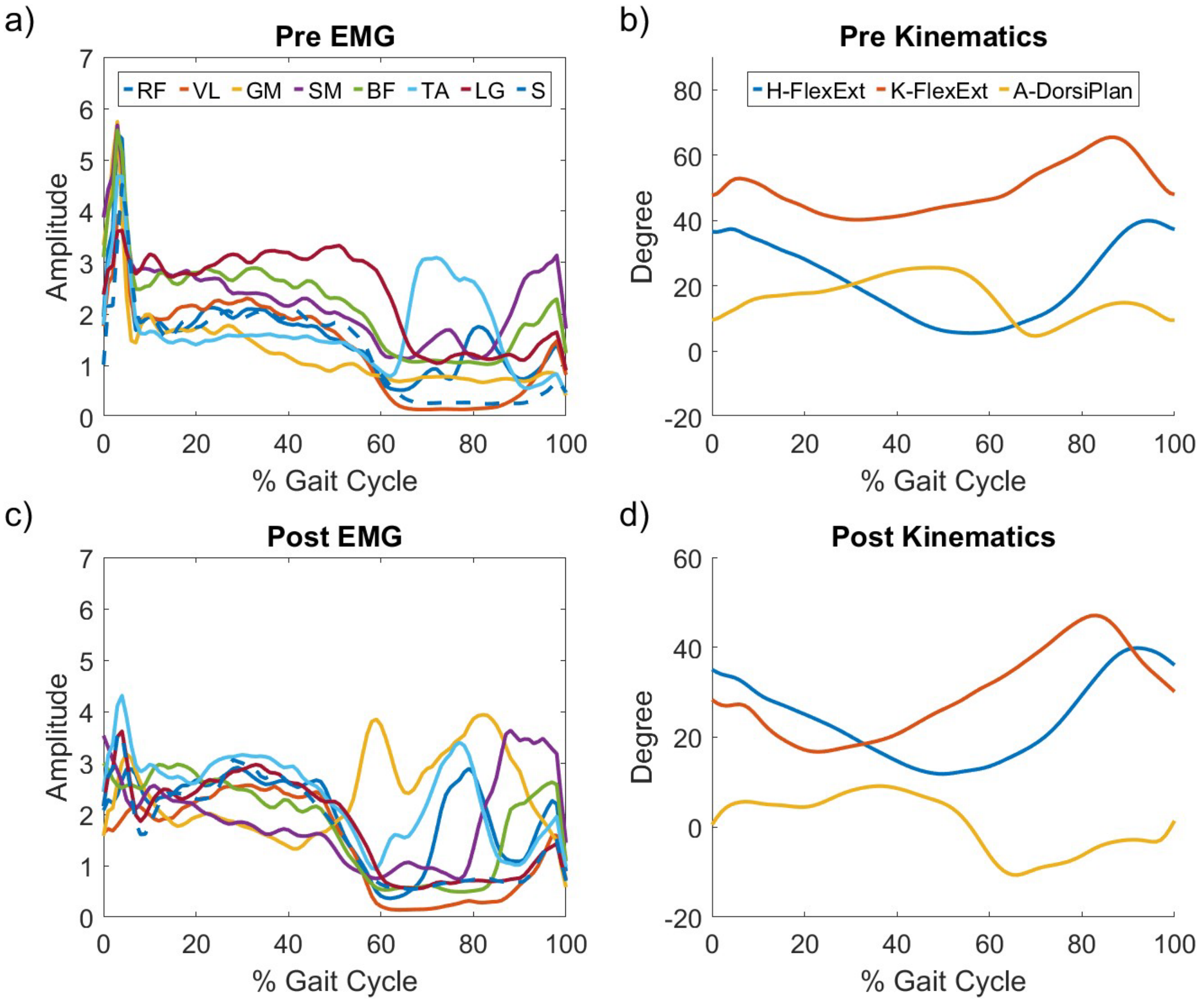

Figure 3: Observational changes between pre-surgery and post-surgery assessments for P2-Right. a) Mean pre-surgery EMG. b) Pre-surgery sagittal plane kinematics where H-FlexExt and K-FlexExt are the flexion and extension angles of the hip and knee respectively, while A-DorsiPlan is the ankle dorsiflexion and plantar flexion angle c) Mean post-surgery EMG. d) Post-surgery sagittal plane kinematics.

Supplementary Data to the article "Influence of surgical intervention on pre- and post-surgery patient specific muscle synergies in children with cerebral palsy", by Tiana Breust, Jiayin Lin, Vincent C. K. Cheung, Firooz Salami, Sebastian I. Wolf, Gursel Alici and Manish Sreenivasa

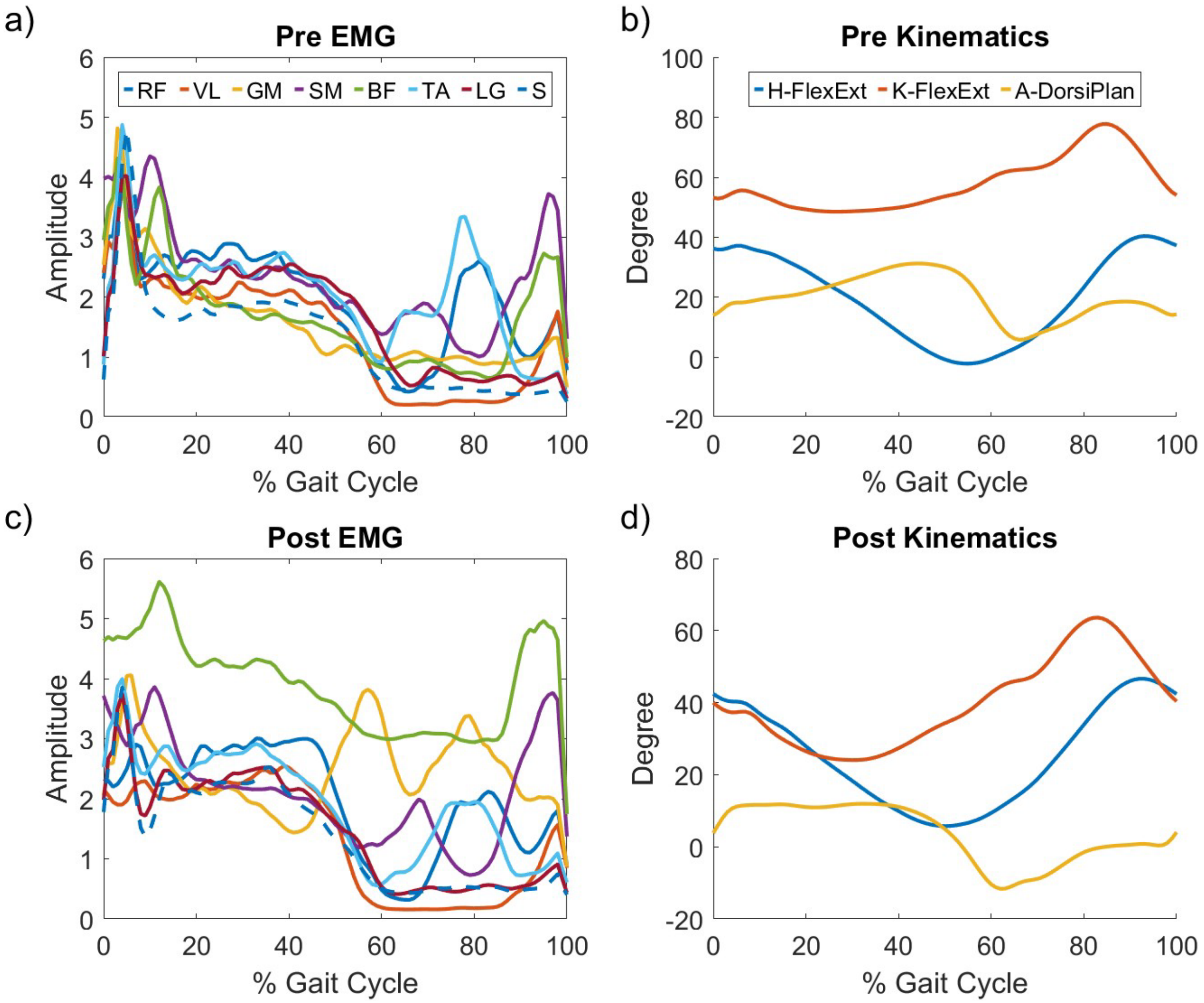

Figure 4: Observational changes between pre-surgery and post-surgery assessments for P2-Left. a) Mean pre-surgery EMG. b) Pre-surgery sagittal plane kinematics where H-FlexExt and K-FlexExt are the flexion and extension angles of the hip and knee respectively, while A-DorsiPlan is the ankle dorsiflexion and plantar flexion angle c) Mean post-surgery EMG. d) Post-surgery sagittal plane kinematics.

Supplementary Data to the article "Influence of surgical intervention on pre- and post-surgery patient specific muscle synergies in children with cerebral palsy", by Tiana Breust, Jiayin Lin, Vincent C. K. Cheung, Firooz Salami, Sebastian I. Wolf, Gursel Alici and Manish Sreenivasa

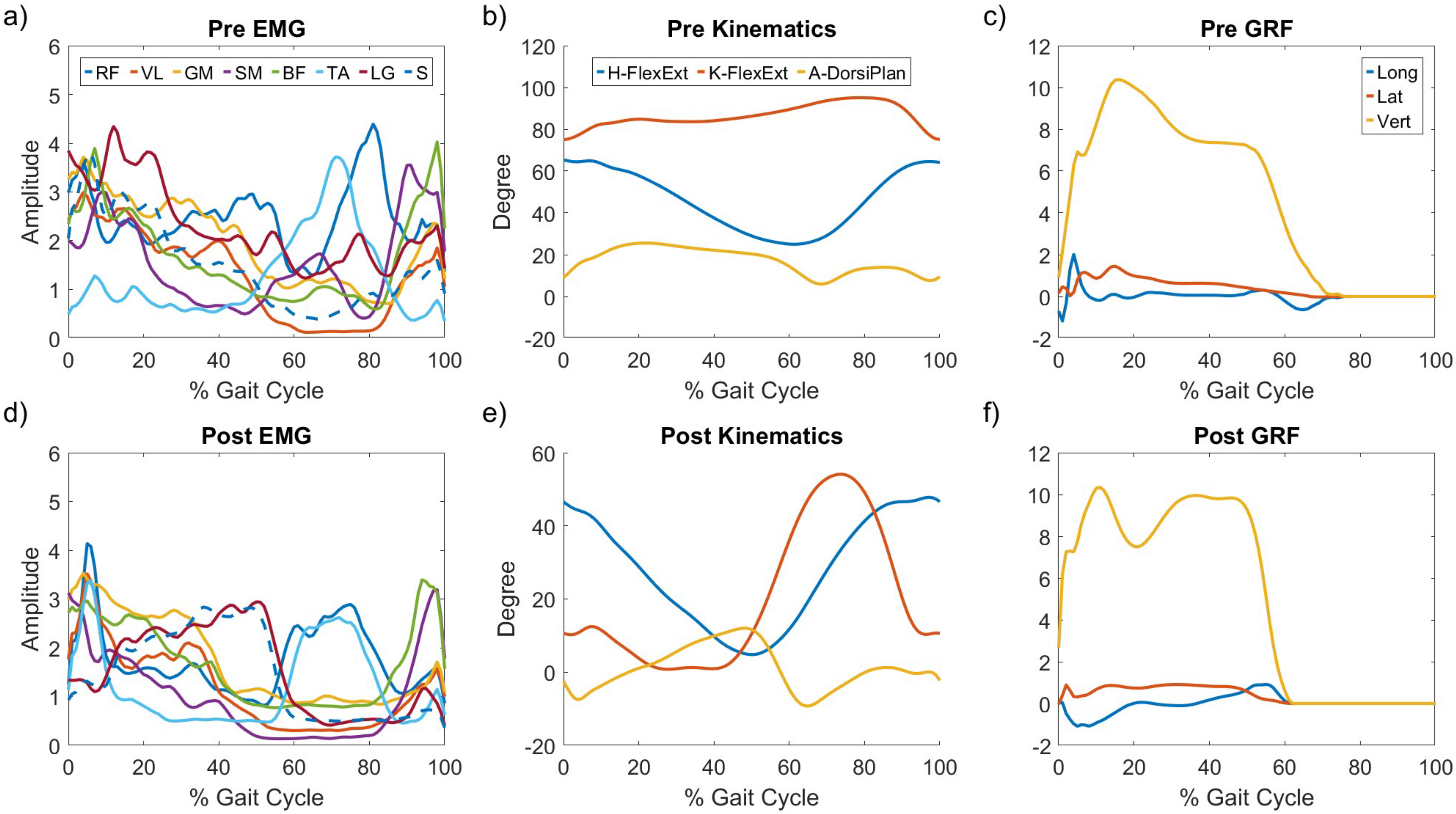

Figure 5: Observational changes between pre-surgery and post-surgery assessments for P3-Right. a) Mean pre-surgery EMG. b) Pre-surgery sagittal plane kinematics where H-FlexExt and K-FlexExt are the flexion and extension angles of the hip and knee respectively, while A-DorsiPlan is the ankle dorsiflexion and plantar flexion angle c) Plot of pre-surgery ground reaction forces including Longitudinal (Long), Lateral (Lat), and Vertical (Vert). d) Mean post-surgery EMG. e) Post-surgery sagittal plane kinematics. f) Plot of post-surgery ground reaction forces.

Supplementary Data to the article "Influence of surgical intervention on pre- and post-surgery patient specific muscle synergies in children with cerebral palsy", by Tiana Breust, Jiayin Lin, Vincent C. K. Cheung, Firooz Salami, Sebastian I. Wolf, Gursel Alici and Manish Sreenivasa

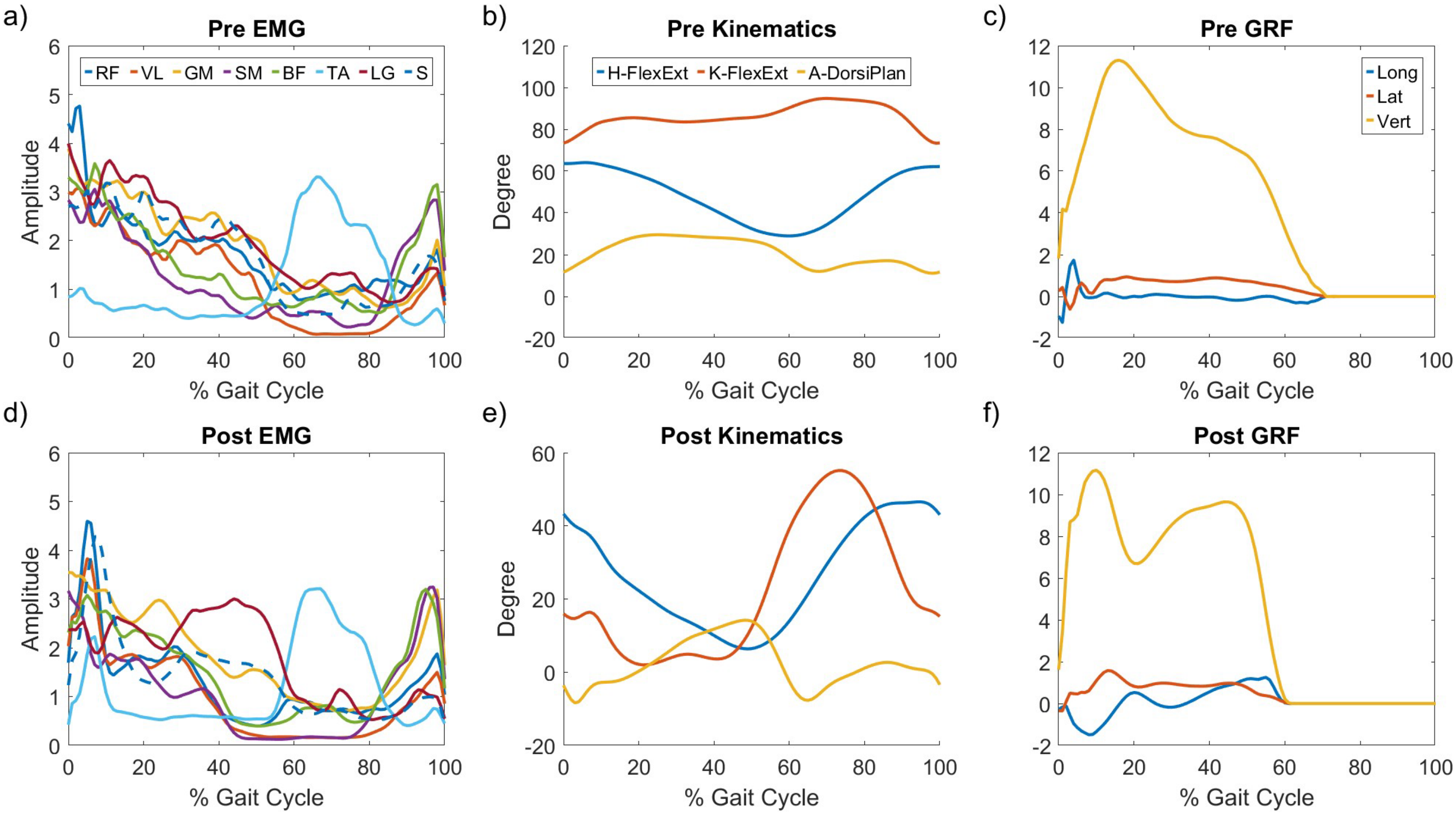

Figure 6: Observational changes between pre-surgery and post-surgery assessments for P3-Left. a) Mean pre-surgery EMG. b) Pre-surgery sagittal plane kinematics where H-FlexExt and K-FlexExt are the flexion and extension angles of the hip and knee respectively, while A-DorsiPlan is the ankle dorsiflexion and plantar flexion angle c) Plot of pre-surgery ground reaction forces including Longitudinal (Long), Lateral (Lat), and Vertical (Vert). d) Mean post-surgery EMG. e) Post-surgery sagittal plane kinematics. f) Plot of post-surgery ground reaction forces.

Supplementary Data to the article "Influence of surgical intervention on pre- and post-surgery patient specific muscle synergies in children with cerebral palsy", by Tiana Breust, Jiayin Lin, Vincent C. K. Cheung, Firooz Salami, Sebastian I. Wolf, Gursel Alici and Manish Sreenivasa

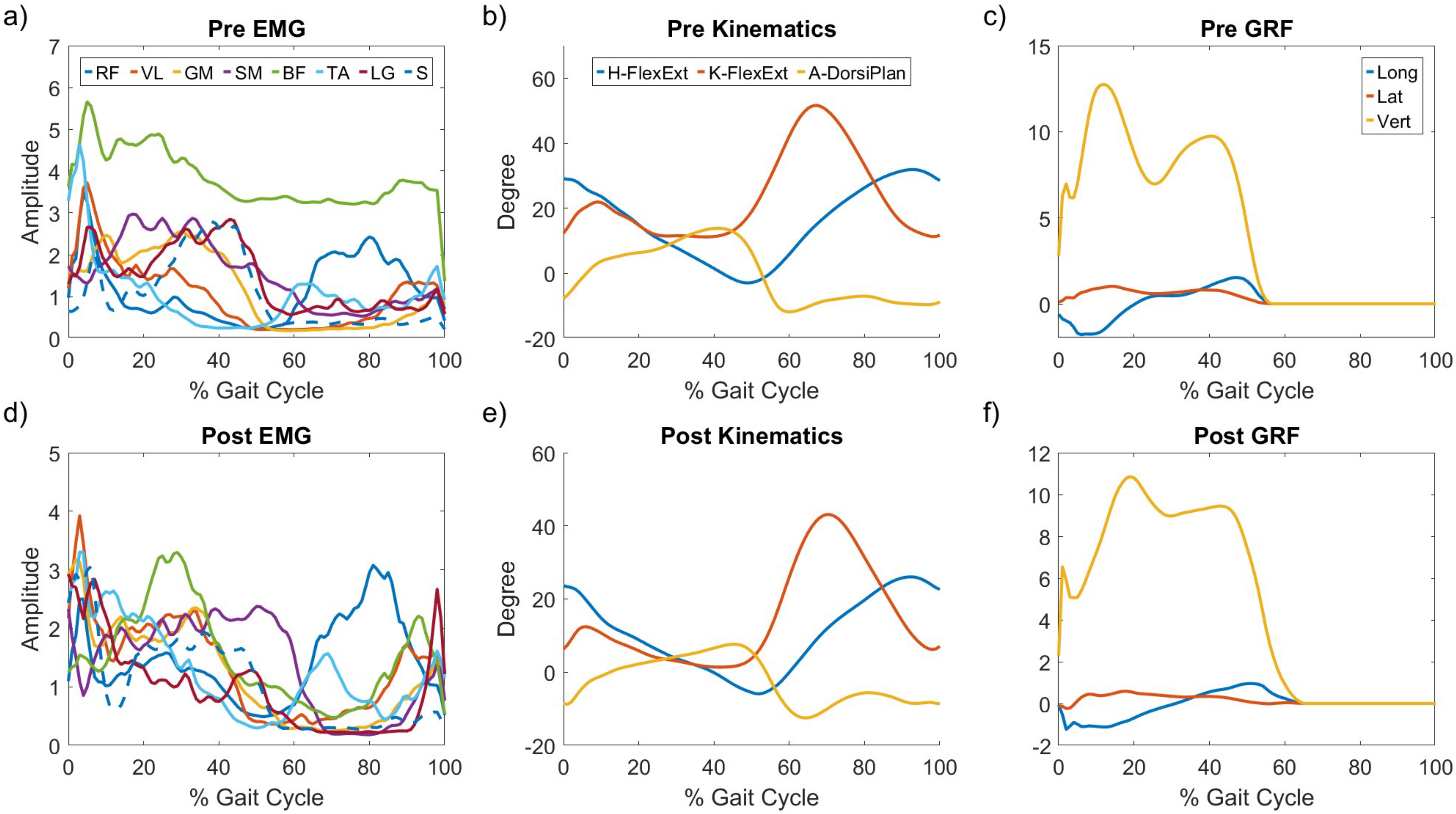

Figure 7: Observational changes between pre-surgery and post-surgery assessments for P4-Right. a) Mean pre-surgery EMG. b) Pre-surgery sagittal plane kinematics where H-FlexExt and K-FlexExt are the flexion and extension angles of the hip and knee respectively, while A-DorsiPlan is the ankle dorsiflexion and plantar flexion angle c) Plot of pre-surgery ground reaction forces including Longitudinal (Long), Lateral (Lat), and Vertical (Vert). d) Mean post-surgery EMG. e) Post-surgery sagittal plane kinematics. f) Plot of post-surgery ground reaction forces.

Supplementary Data to the article "Influence of surgical intervention on pre- and post-surgery patient specific muscle synergies in children with cerebral palsy", by Tiana Breust, Jiayin Lin, Vincent C. K. Cheung, Firooz Salami, Sebastian I. Wolf, Gursel Alici and Manish Sreenivasa

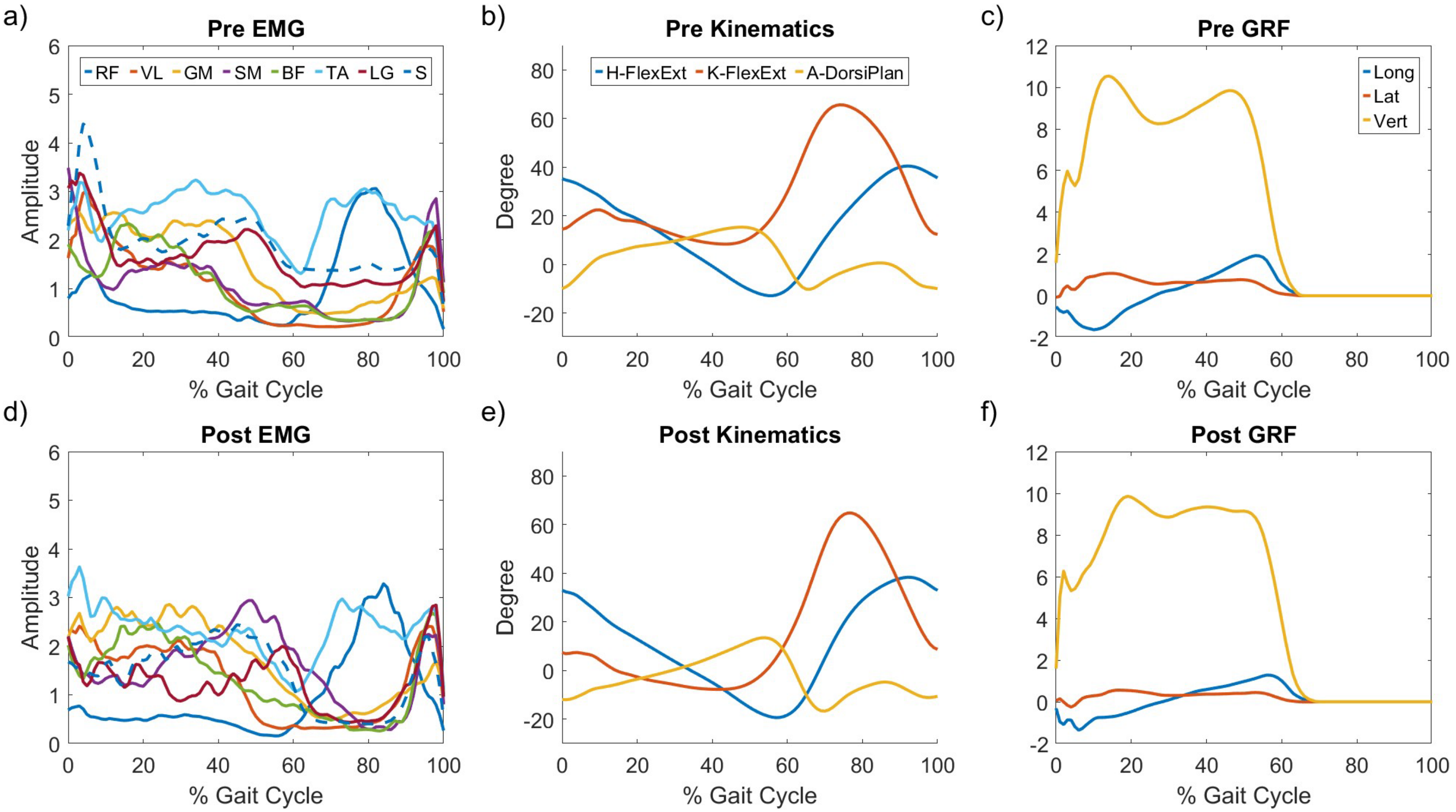

Figure 8: Observational changes between pre-surgery and post-surgery assessments for P4-Left. a) Mean pre-surgery EMG. b) Pre-surgery sagittal plane kinematics where H-FlexExt and K-FlexExt are the flexion and extension angles of the hip and knee respectively, while A-DorsiPlan is the ankle dorsiflexion and plantar flexion angle c) Plot of pre-surgery ground reaction forces including Longitudinal (Long), Lateral (Lat), and Vertical (Vert). d) Mean post-surgery EMG. e) Post-surgery sagittal plane kinematics. f) Plot of post-surgery ground reaction forces.

Supplementary Data to the article "Influence of surgical intervention on pre- and post-surgery patient specific muscle synergies in children with cerebral palsy", by Tiana Breust, Jiayin Lin, Vincent C. K. Cheung, Firooz Salami, Sebastian I. Wolf, Gursel Alici and Manish Sreenivasa

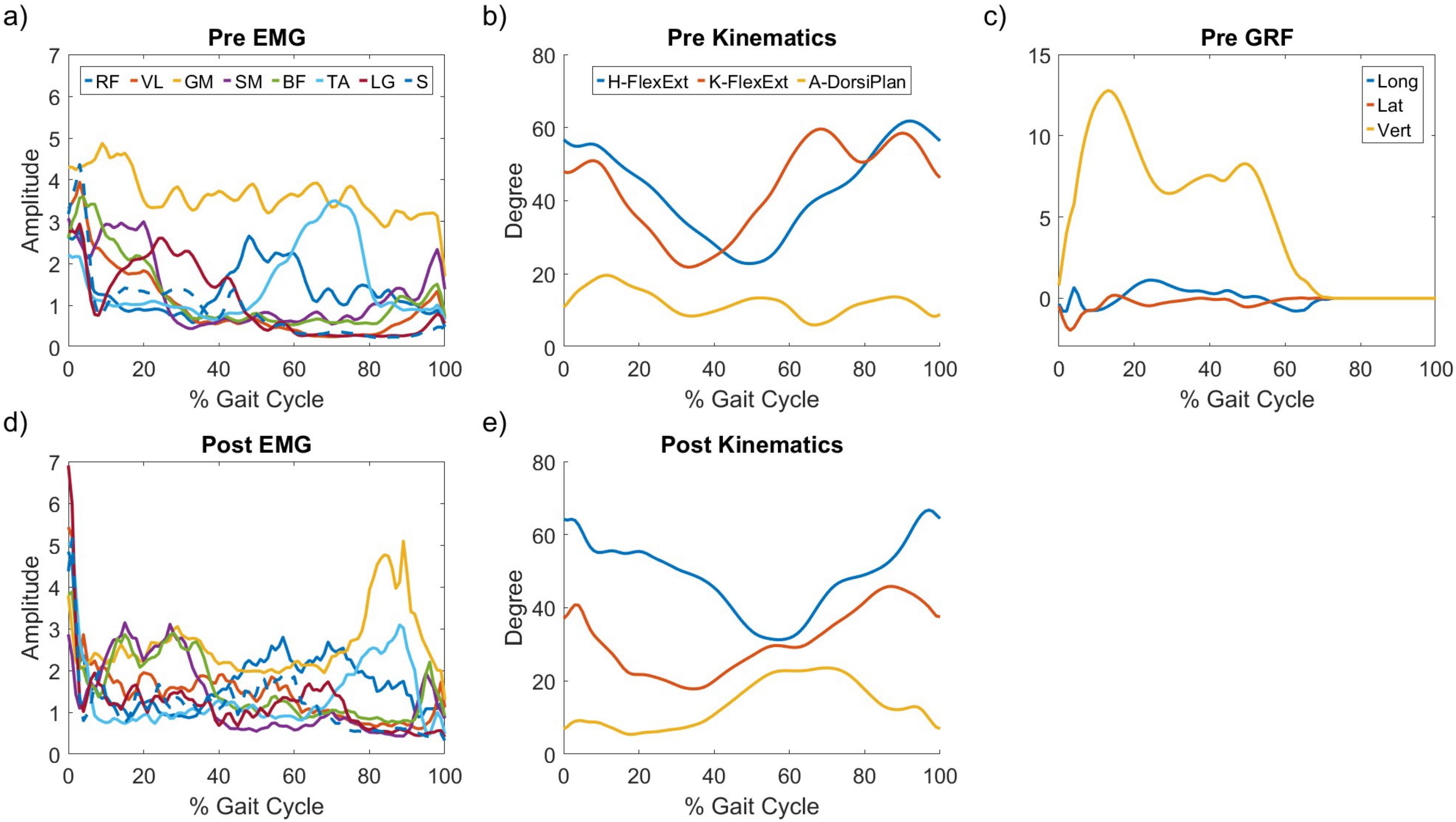

Figure 9: Observational changes between pre-surgery and post-surgery assessments for P5-Right. a) Mean pre-surgery EMG. b) Pre-surgery sagittal plane kinematics where H-FlexExt and K-FlexExt are the flexion and extension angles of the hip and knee respectively, while A-DorsiPlan is the ankle dorsiflexion and plantar flexion angle c) Plot of pre-surgery ground reaction forces including Longitudinal (Long), Lateral (Lat), and Vertical (Vert). d) Mean post-surgery EMG. e) Post-surgery sagittal plane kinematics.

Supplementary Data to the article "Influence of surgical intervention on pre- and post-surgery patient specific muscle synergies in children with cerebral palsy", by Tiana Breust, Jiayin Lin, Vincent C. K. Cheung, Firooz Salami, Sebastian I. Wolf, Gursel Alici and Manish Sreenivasa

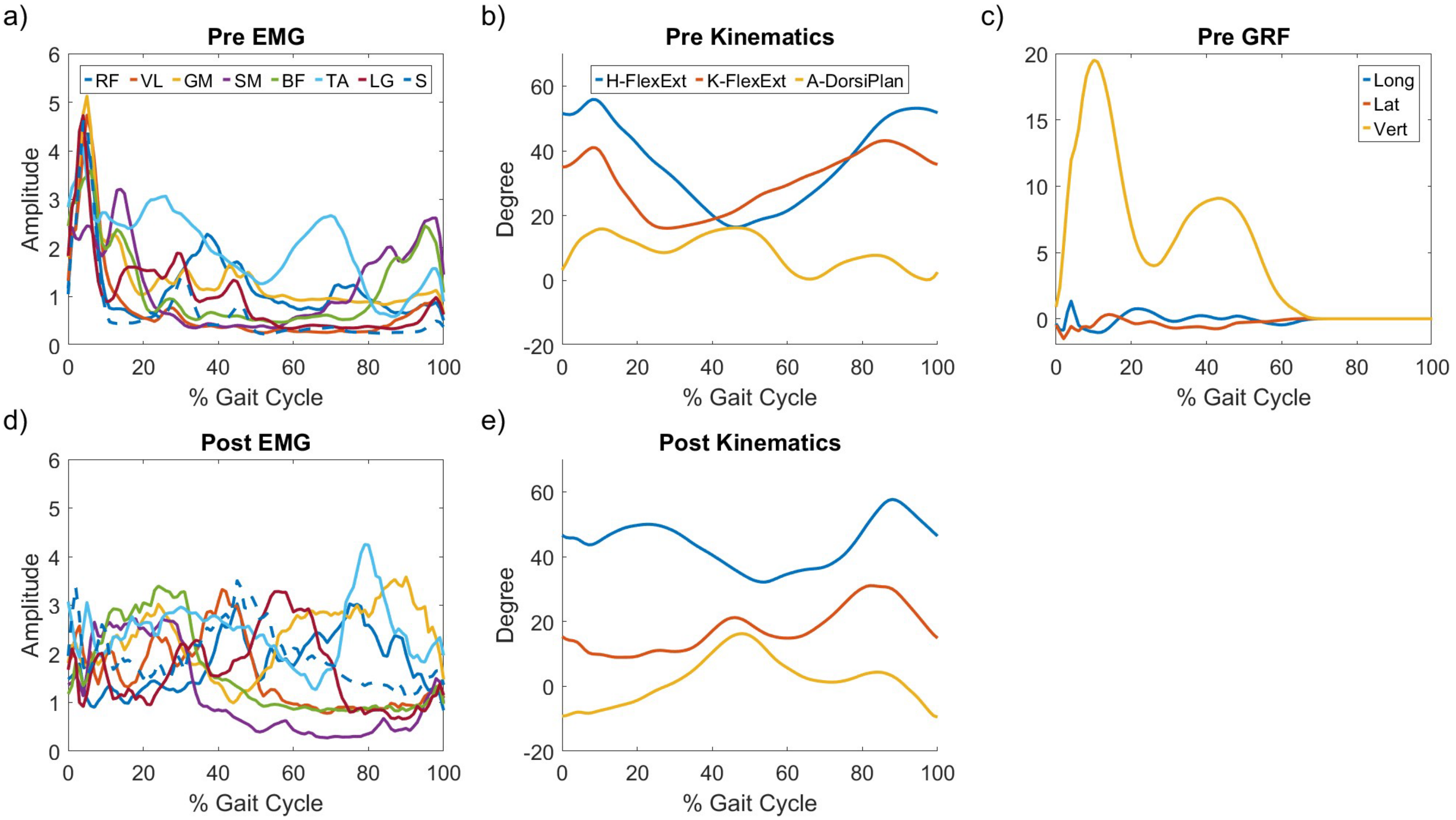

Figure 10: Observational changes between pre-surgery and post-surgery assessments for P5-Left. a) Mean pre-surgery EMG. b) Pre-surgery sagittal plane kinematics where H-FlexExt and K-FlexExt are the flexion and extension angles of the hip and knee respectively, while A-DorsiPlan is the ankle dorsiflexion and plantar flexion angle c) Plot of pre-surgery ground reaction forces including Longitudinal (Long), Lateral (Lat), and Vertical (Vert). d) Mean post-surgery EMG. e) Post-surgery sagittal plane kinematics.

Supplementary Data to the article "Influence of surgical intervention on pre- and post-surgery patient specific muscle synergies in children with cerebral palsy", by Tiana Breust, Jiayin Lin, Vincent C. K. Cheung, Firooz Salami, Sebastian I. Wolf, Gursel Alici and Manish Sreenivasa

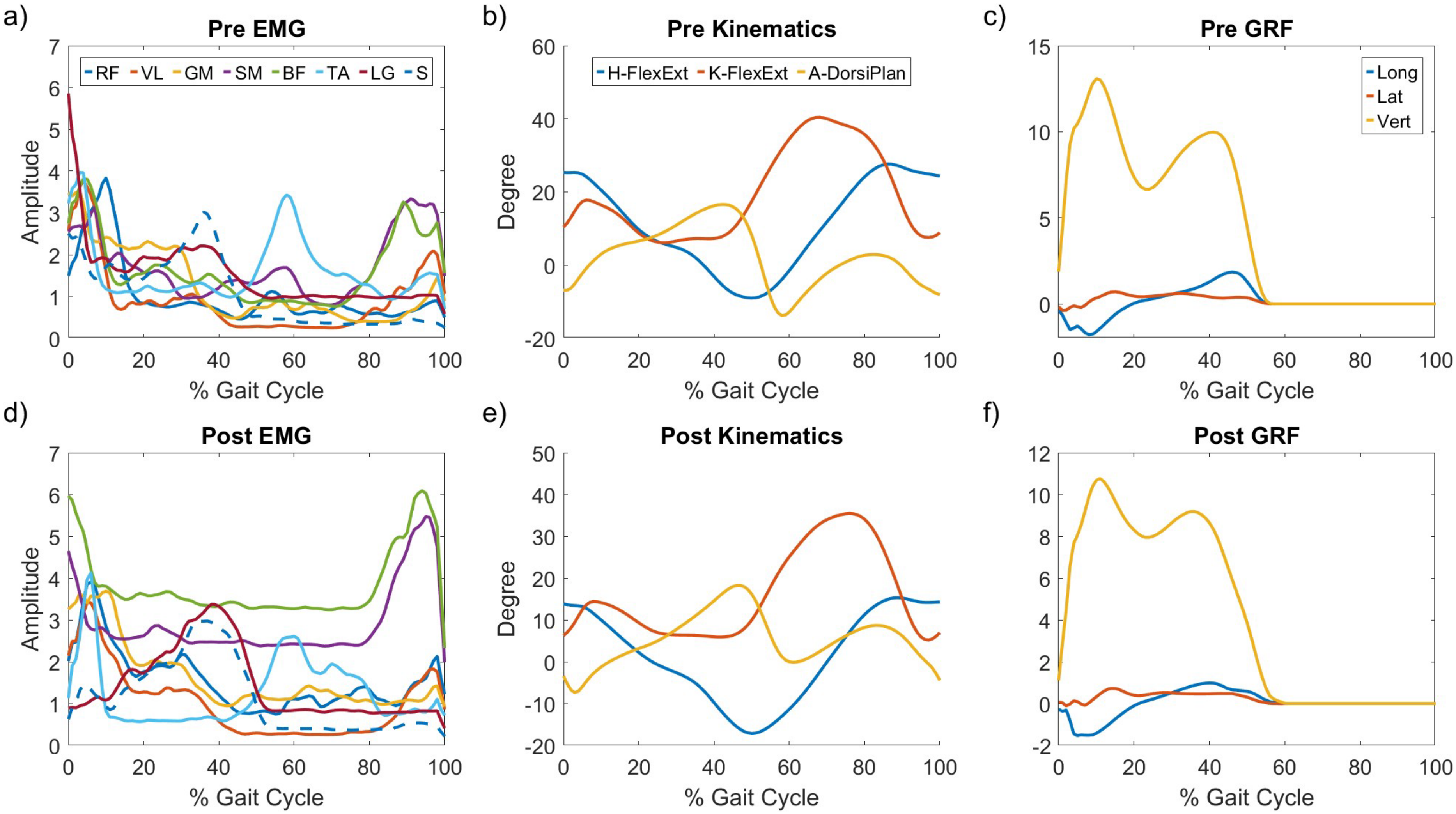

Figure 11: Observational changes between pre-surgery and post-surgery assessments for P6-Right. a) Mean pre-surgery EMG. b) Pre-surgery sagittal plane kinematics where H-FlexExt and K-FlexExt are the flexion and extension angles of the hip and knee respectively, while A-DorsiPlan is the ankle dorsiflexion and plantar flexion angle c) Plot of pre-surgery ground reaction forces including Longitudinal (Long), Lateral (Lat), and Vertical (Vert). d) Mean post-surgery EMG. e) Post-surgery sagittal plane kinematics. f) Plot of post-surgery ground reaction forces.

Supplementary Data to the article "Influence of surgical intervention on pre- and post-surgery patient specific muscle synergies in children with cerebral palsy", by Tiana Breust, Jiayin Lin, Vincent C. K. Cheung, Firooz Salami, Sebastian I. Wolf, Gursel Alici and Manish Sreenivasa

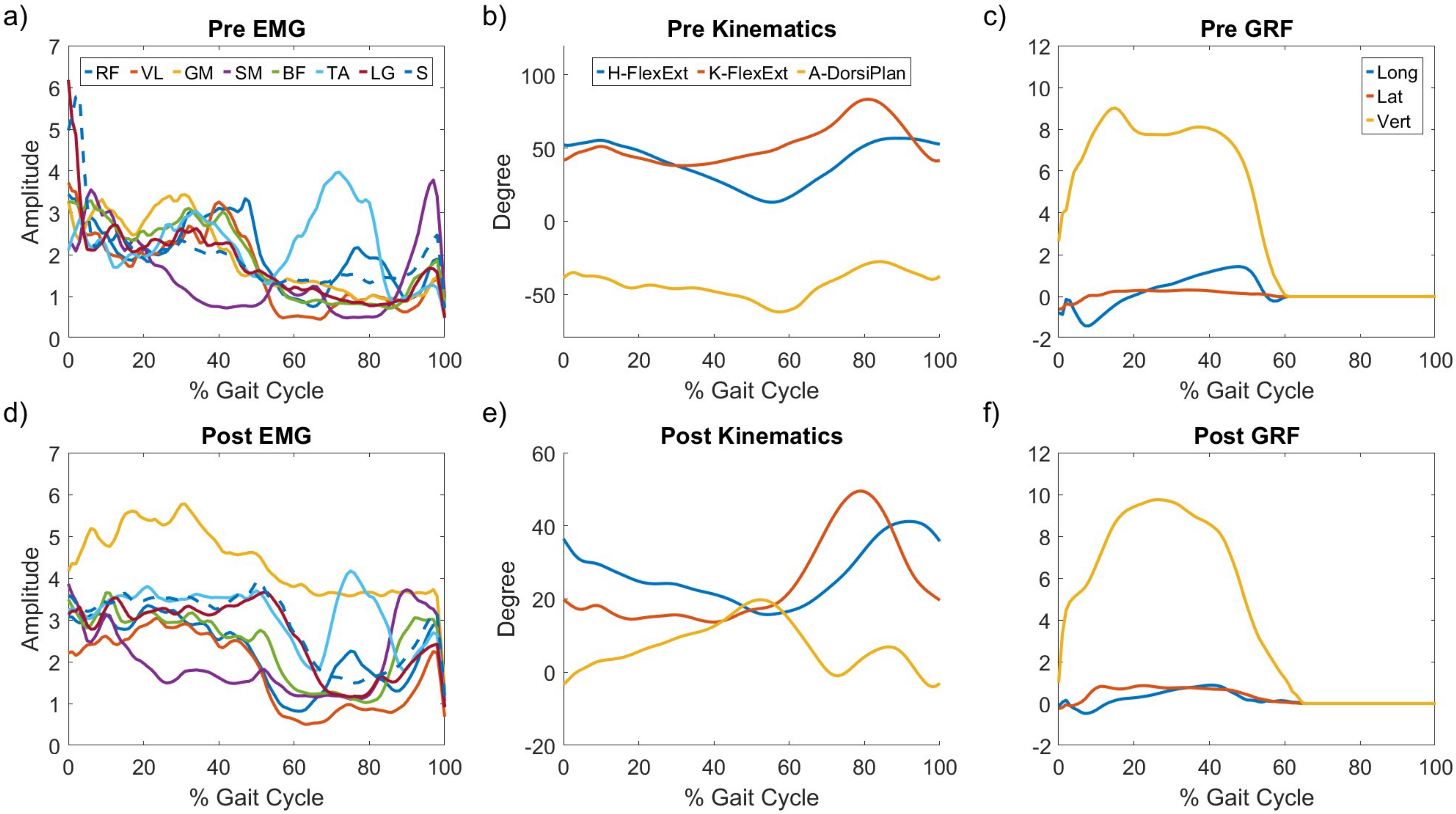

Figure 12: Observational changes between pre-surgery and post-surgery assessments for P7-Right. a) Mean pre-surgery EMG. b) Pre-surgery sagittal plane kinematics where H-FlexExt and K-FlexExt are the flexion and extension angles of the hip and knee respectively, while A-DorsiPlan is the ankle dorsiflexion and plantar flexion angle c) Plot of pre-surgery ground reaction forces including Longitudinal (Long), Lateral (Lat), and Vertical (Vert). d) Mean post-surgery EMG. e) Post-surgery sagittal plane kinematics. f) Plot of post-surgery ground reaction forces.

Supplementary Data to the article "Influence of surgical intervention on pre- and post-surgery patient specific muscle synergies in children with cerebral palsy", by Tiana Breust, Jiayin Lin, Vincent C. K. Cheung, Firooz Salami, Sebastian I. Wolf, Gursel Alici and Manish Sreenivasa

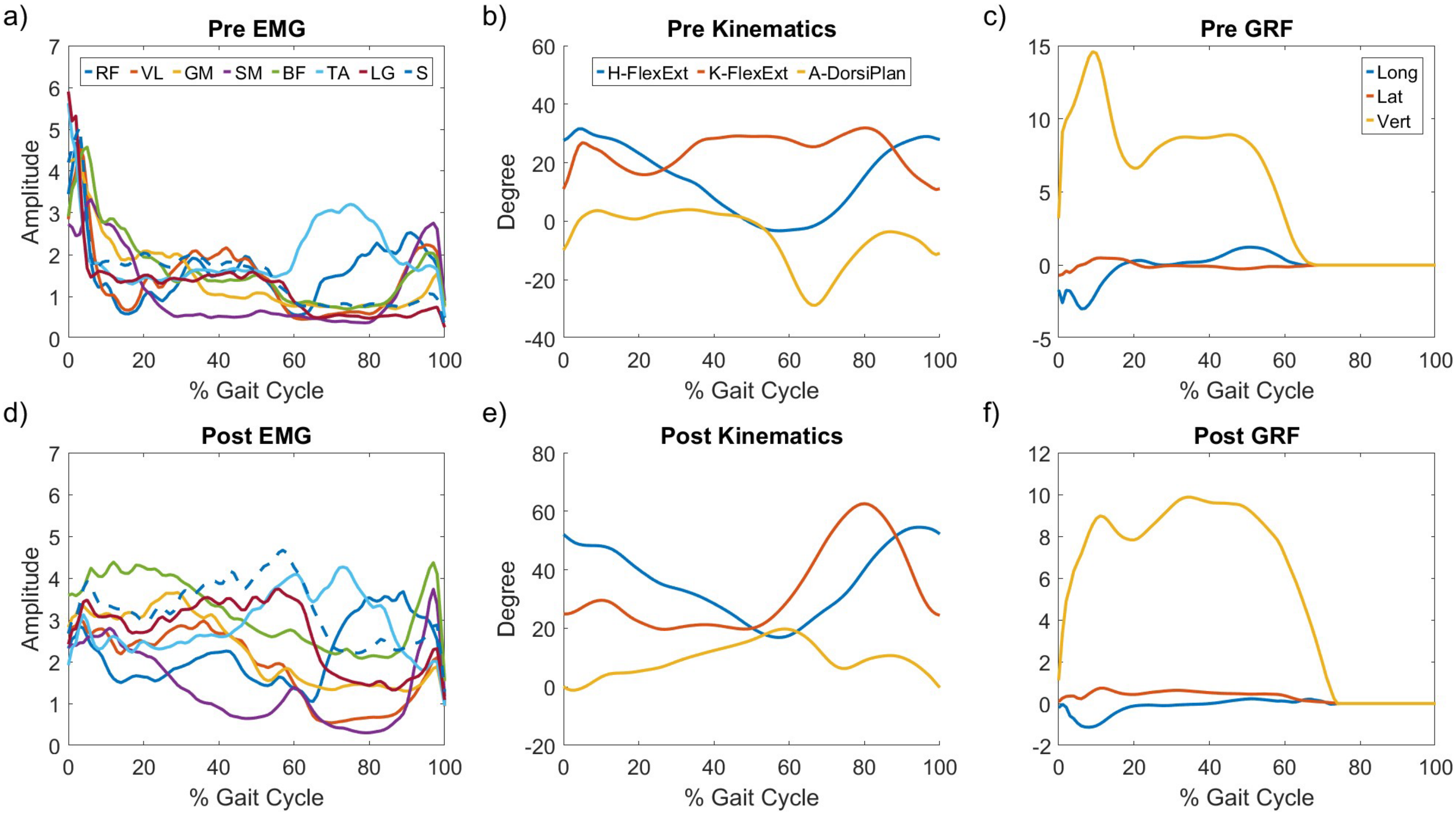

Figure 13: Observational changes between pre-surgery and post-surgery assessments for P7-Left. a) Mean pre-surgery EMG. b) Pre-surgery sagittal plane kinematics where H-FlexExt and K-FlexExt are the flexion and extension angles of the hip and knee respectively, while A-DorsiPlan is the ankle dorsiflexion and plantar flexion angle c) Plot of pre-surgery ground reaction forces including Longitudinal (Long), Lateral (Lat), and Vertical (Vert). d) Mean post-surgery EMG. e) Post-surgery sagittal plane kinematics. f) Plot of post-surgery ground reaction forces.

Supplementary Data to the article "Influence of surgical intervention on pre- and post-surgery patient specific muscle synergies in children with cerebral palsy", by Tiana Breust, Jiayin Lin, Vincent C. K. Cheung, Firooz Salami, Sebastian I. Wolf, Gursel Alici and Manish Sreenivasa

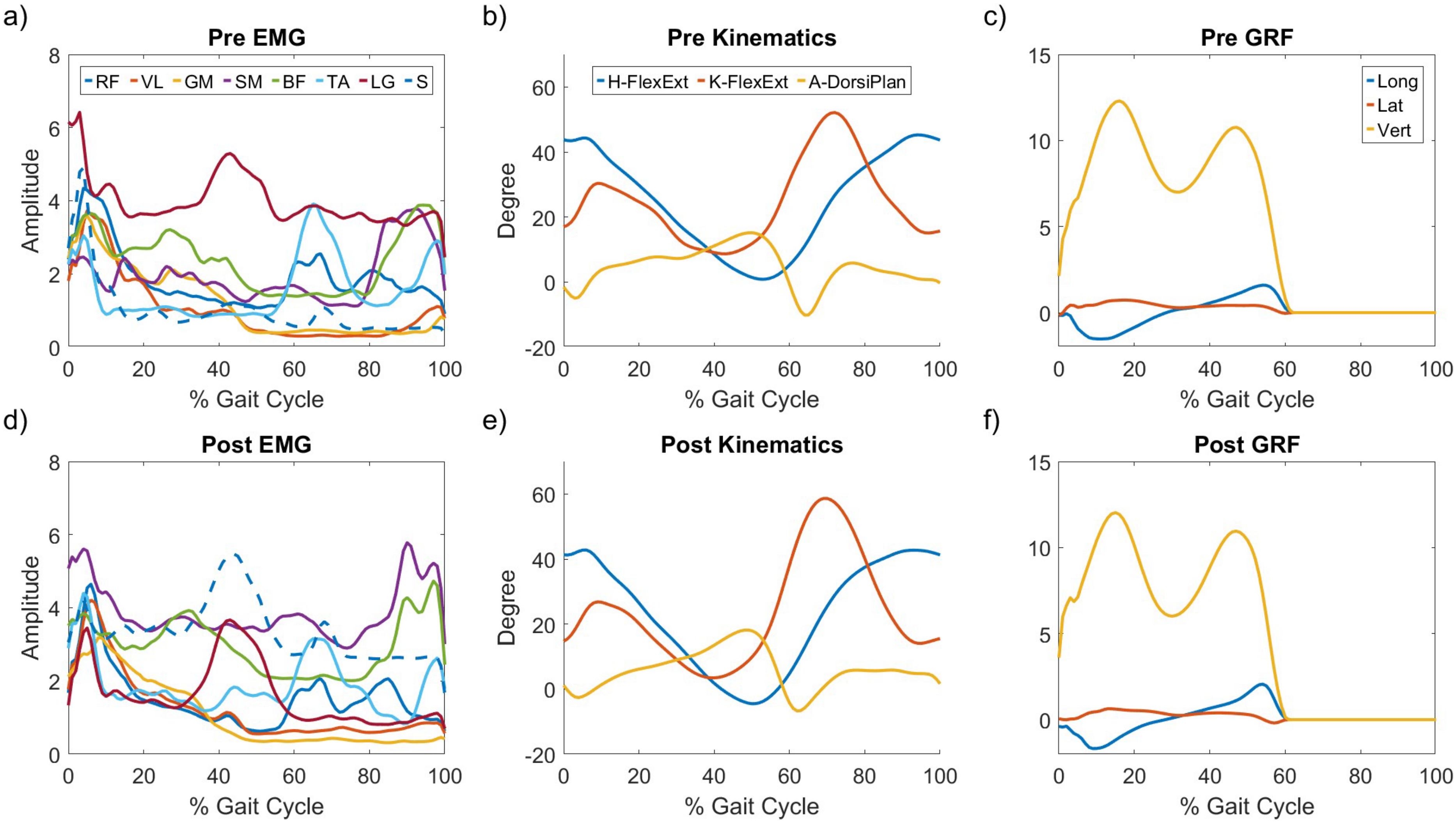

Figure 14: Observational changes between pre-surgery and post-surgery assessments for P8-Right. a) Mean pre-surgery EMG. b) Pre-surgery sagittal plane kinematics where H-FlexExt and K-FlexExt are the flexion and extension angles of the hip and knee respectively, while A-DorsiPlan is the ankle dorsiflexion and plantar flexion angle c) Plot of pre-surgery ground reaction forces including Longitudinal (Long), Lateral (Lat), and Vertical (Vert). d) Mean post-surgery EMG. e) Post-surgery sagittal plane kinematics. f) Plot of post-surgery ground reaction forces.

Supplementary Data to the article "Influence of surgical intervention on pre- and post-surgery patient specific muscle synergies in children with cerebral palsy", by Tiana Breust, Jiayin Lin, Vincent C. K. Cheung, Firooz Salami, Sebastian I. Wolf, Gursel Alici and Manish Sreenivasa

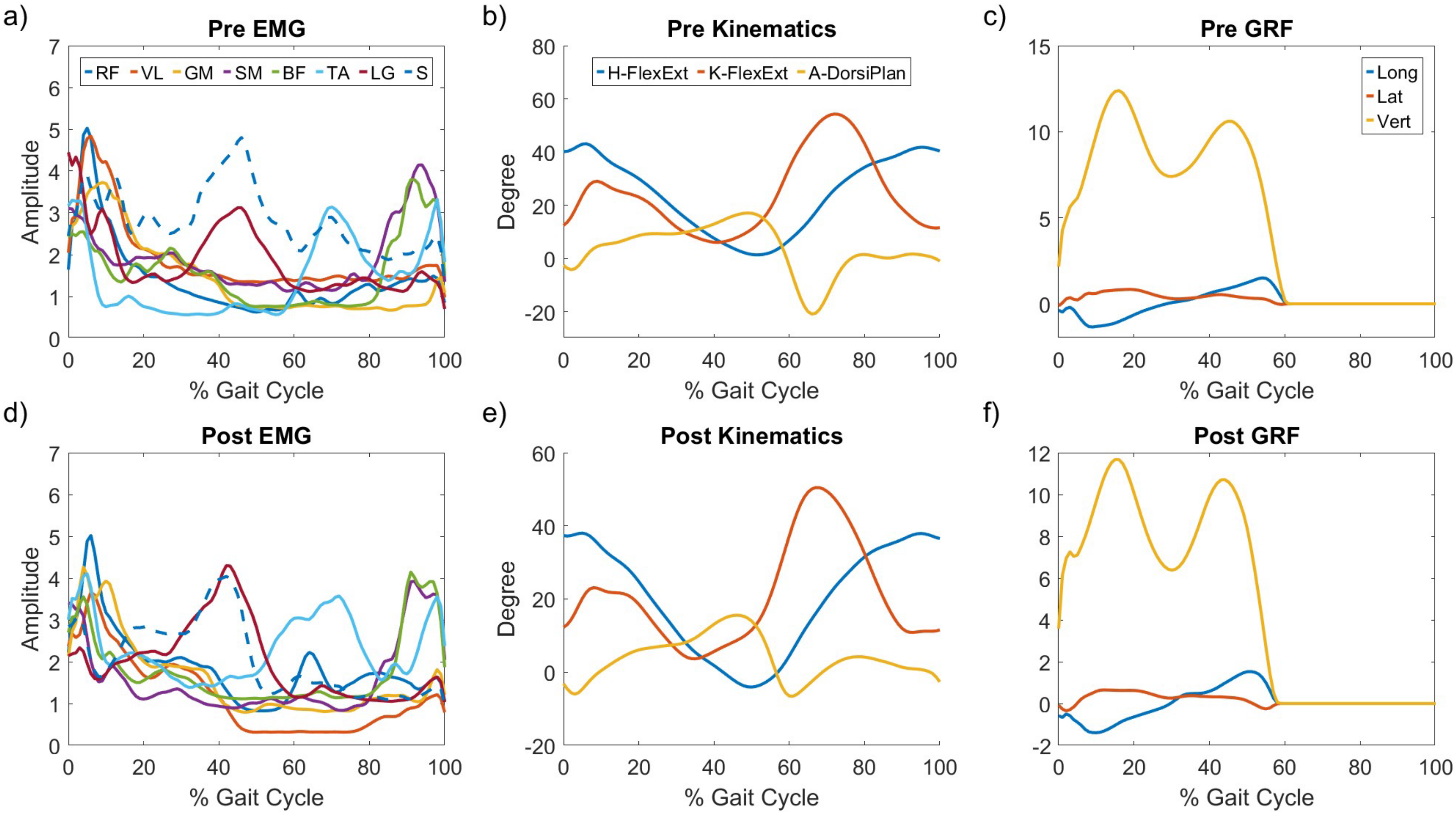

Figure 15: Observational changes between pre-surgery and post-surgery assessments for P8-Left. a) Mean pre-surgery EMG. b) Pre-surgery sagittal plane kinematics where H-FlexExt and K-FlexExt are the flexion and extension angles of the hip and knee respectively, while A-DorsiPlan is the ankle dorsiflexion and plantar flexion angle c) Plot of pre-surgery ground reaction forces including Longitudinal (Long), Lateral (Lat), and Vertical (Vert). d) Mean post-surgery EMG. e) Post-surgery sagittal plane kinematics. f) Plot of post-surgery ground reaction forces.

Supplementary Data to the article "Influence of surgical intervention on pre- and post-surgery patient specific muscle synergies in children with cerebral palsy", by Tiana Breust, Jiayin Lin, Vincent C. K. Cheung, Firooz Salami, Sebastian I. Wolf, Gursel Alici and Manish Sreenivasa

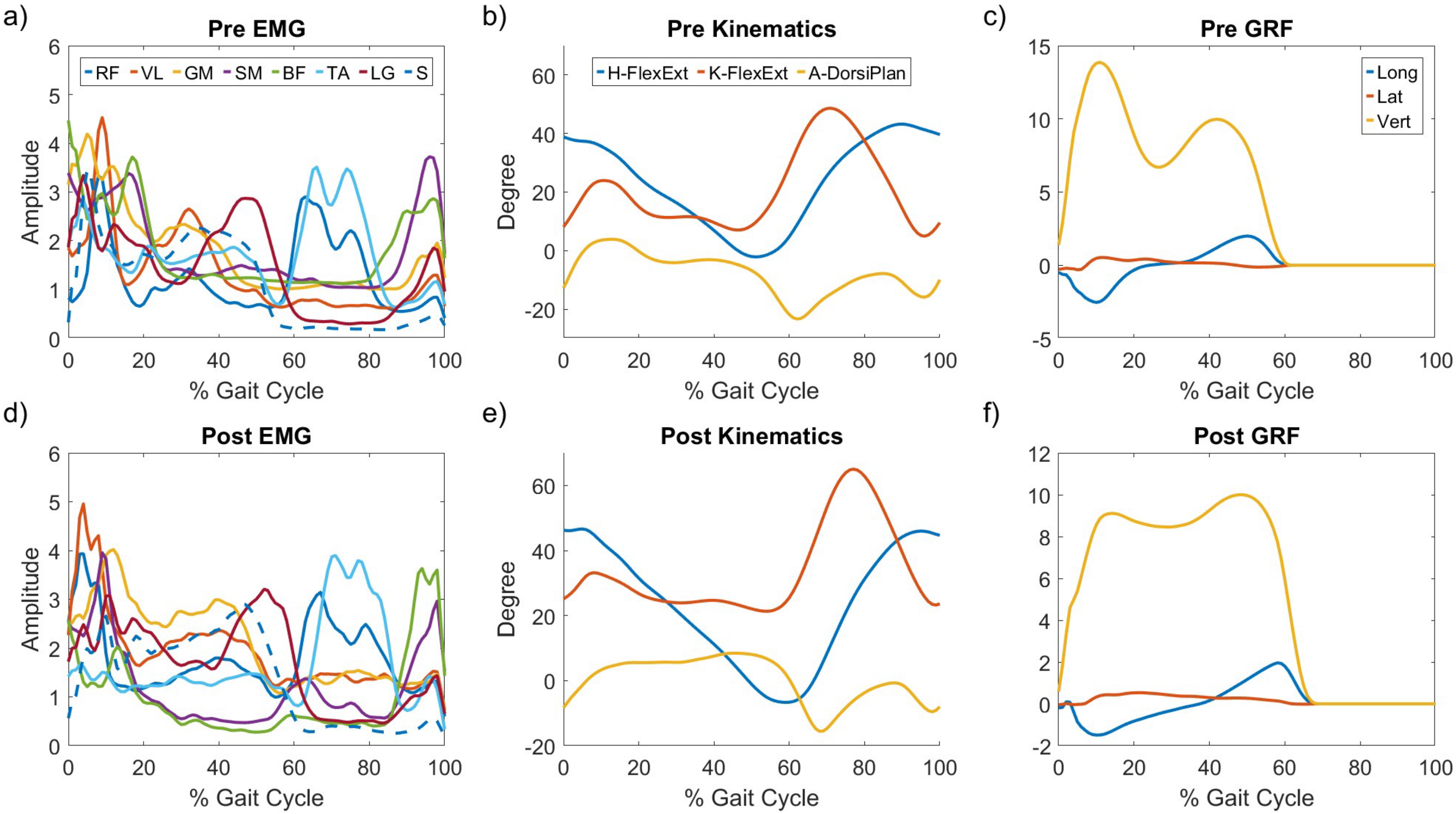

Figure 16: Observational changes between pre-surgery and post-surgery assessments for P9-Right. a) Mean pre-surgery EMG. b) Pre-surgery sagittal plane kinematics where H-FlexExt and K-FlexExt are the flexion and extension angles of the hip and knee respectively, while A-DorsiPlan is the ankle dorsiflexion and plantar flexion angle c) Plot of pre-surgery ground reaction forces including Longitudinal (Long), Lateral (Lat), and Vertical (Vert). d) Mean post-surgery EMG. e) Post-surgery sagittal plane kinematics. f) Plot of post-surgery ground reaction forces.

Supplementary Data to the article "Influence of surgical intervention on pre- and post-surgery patient specific muscle synergies in children with cerebral palsy", by Tiana Breust, Jiayin Lin, Vincent C. K. Cheung, Firooz Salami, Sebastian I. Wolf, Gursel Alici and Manish Sreenivasa

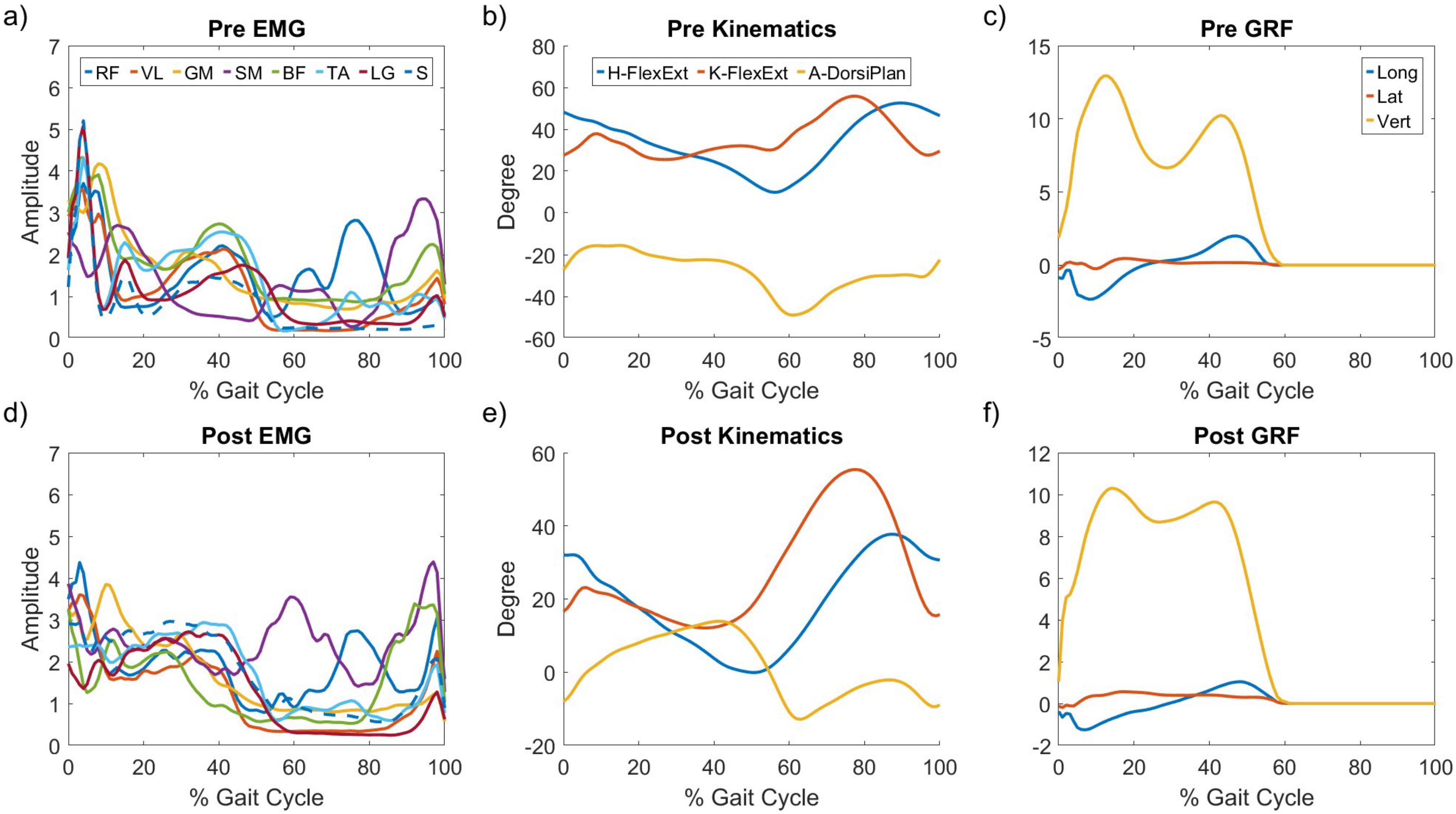

Figure 17: Observational changes between pre-surgery and post-surgery assessments for P9-Left. a) Mean pre-surgery EMG. b) Pre-surgery sagittal plane kinematics where H-FlexExt and K-FlexExt are the flexion and extension angles of the hip and knee respectively, while A-DorsiPlan is the ankle dorsiflexion and plantar flexion angle c) Plot of pre-surgery ground reaction forces including Longitudinal (Long), Lateral (Lat), and Vertical (Vert). d) Mean post-surgery EMG. e) Post-surgery sagittal plane kinematics. f) Plot of post-surgery ground reaction forces.

Supplementary Data to the article "Influence of surgical intervention on pre- and post-surgery patient specific muscle synergies in children with cerebral palsy", by Tiana Breust, Jiayin Lin, Vincent C. K. Cheung, Firooz Salami, Sebastian I. Wolf, Gursel Alici and Manish Sreenivasa

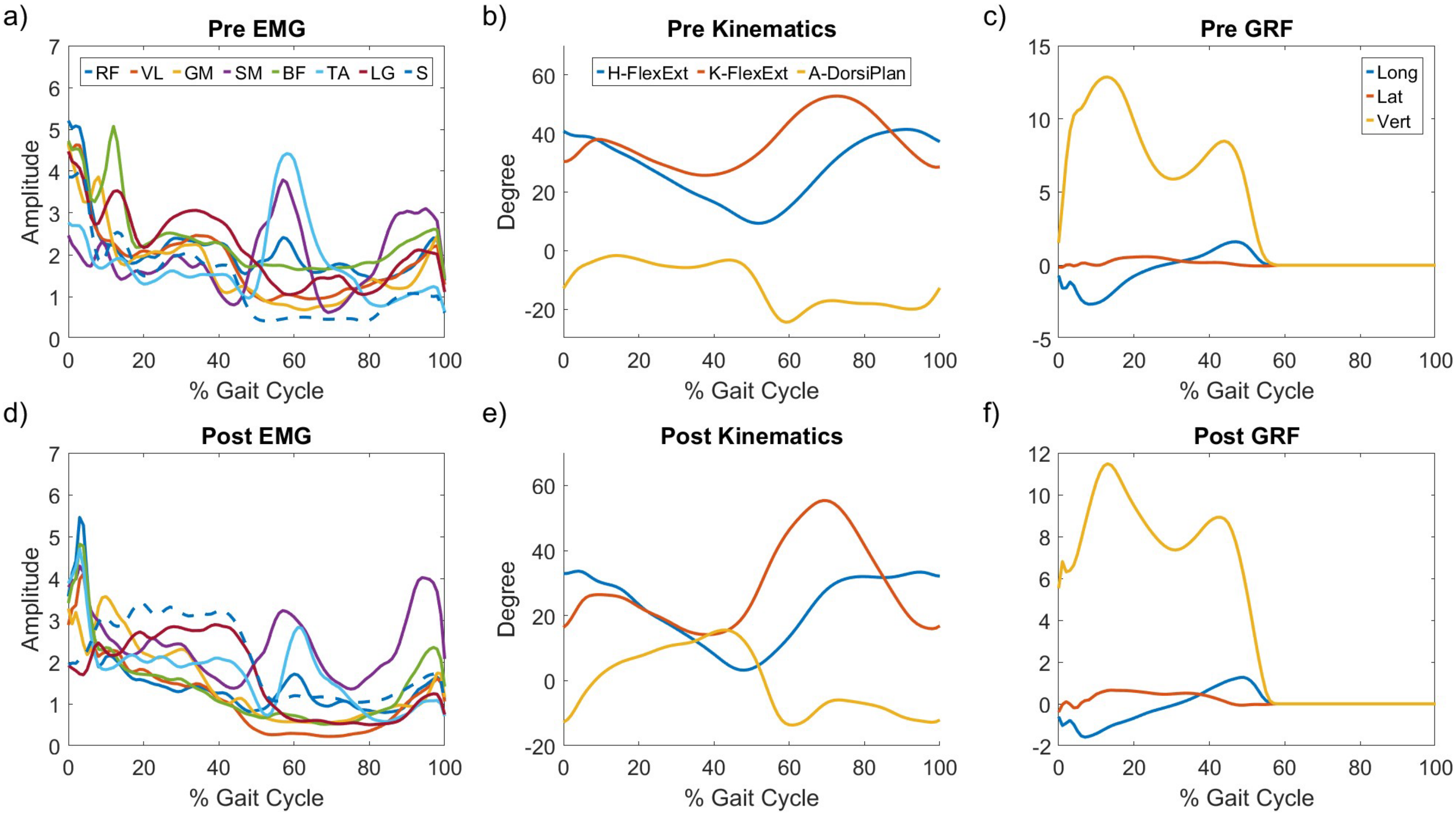

Figure 18: Observational changes between pre-surgery and post-surgery assessments for P10-Right. a) Mean pre-surgery EMG. b) Pre-surgery sagittal plane kinematics where H-FlexExt and K-FlexExt are the flexion and extension angles of the hip and knee respectively, while A-DorsiPlan is the ankle dorsiflexion and plantar flexion angle c) Plot of pre-surgery ground reaction forces including Longitudinal (Long), Lateral (Lat), and Vertical (Vert). d) Mean post-surgery EMG. e) Post-surgery sagittal plane kinematics. f) Plot of post-surgery ground reaction forces.

Supplementary Data to the article "Influence of surgical intervention on pre- and post-surgery patient specific muscle synergies in children with cerebral palsy", by Tiana Breust, Jiayin Lin, Vincent C. K. Cheung, Firooz Salami, Sebastian I. Wolf, Gursel Alici and Manish Sreenivasa

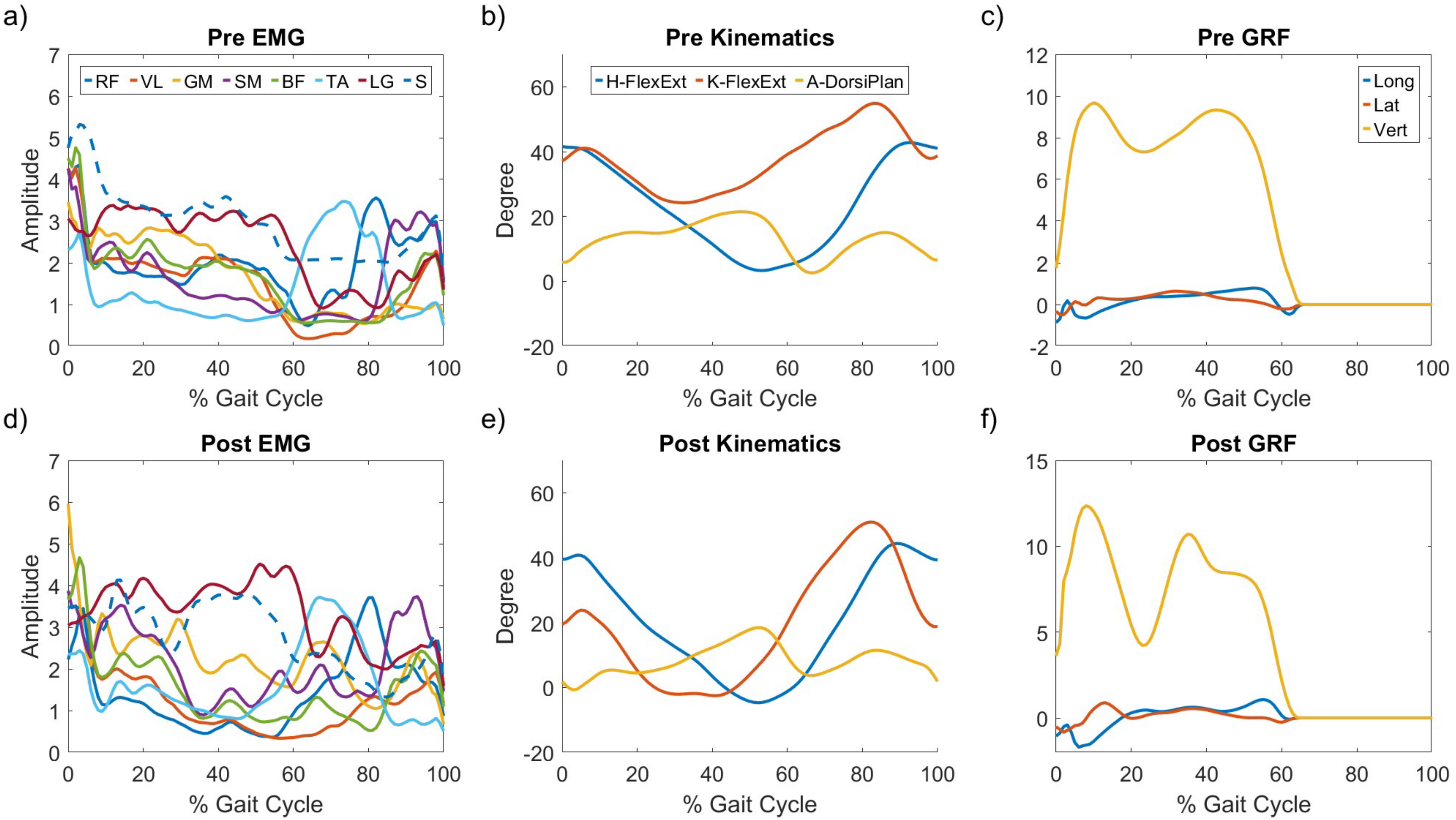

Figure 19: Observational changes between pre-surgery and post-surgery assessments for P11-Right. a) Mean pre-surgery EMG. b) Pre-surgery sagittal plane kinematics where H-FlexExt and K-FlexExt are the flexion and extension angles of the hip and knee respectively, while A-DorsiPlan is the ankle dorsiflexion and plantar flexion angle c) Plot of pre-surgery ground reaction forces including Longitudinal (Long), Lateral (Lat), and Vertical (Vert). d) Mean post-surgery EMG. e) Post-surgery sagittal plane kinematics. f) Plot of post-surgery ground reaction forces.

Supplementary Data to the article "Influence of surgical intervention on pre- and post-surgery patient specific muscle synergies in children with cerebral palsy", by Tiana Breust, Jiayin Lin, Vincent C. K. Cheung, Firooz Salami, Sebastian I. Wolf, Gursel Alici and Manish Sreenivasa

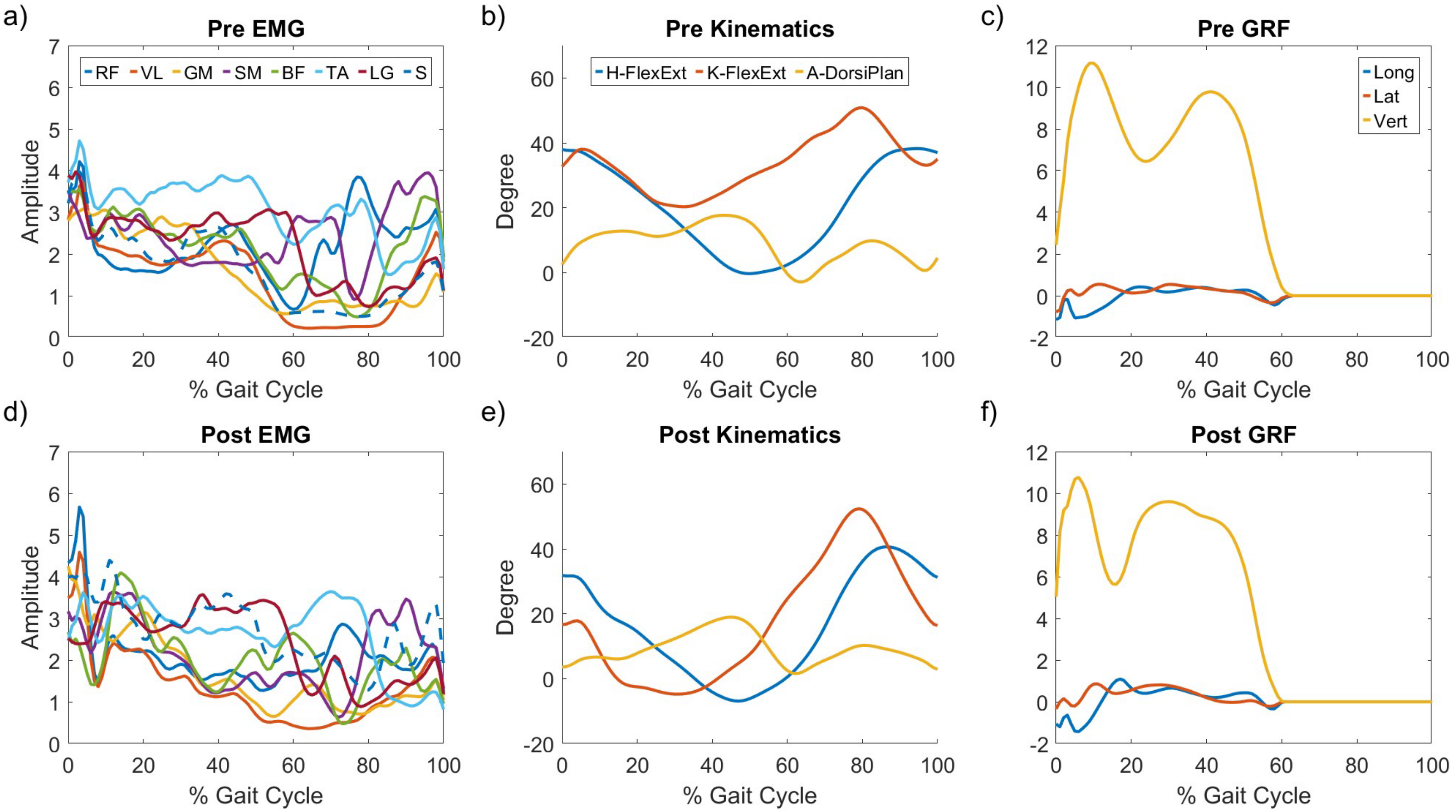

Figure 20: Observational changes between pre-surgery and post-surgery assessments for P11-Left. a) Mean pre-surgery EMG. b) Pre-surgery sagittal plane kinematics where H-FlexExt and K-FlexExt are the flexion and extension angles of the hip and knee respectively, while A-DorsiPlan is the ankle dorsiflexion and plantar flexion angle c) Plot of pre-surgery ground reaction forces including Longitudinal (Long), Lateral (Lat), and Vertical (Vert). d) Mean post-surgery EMG. e) Post-surgery sagittal plane kinematics. f) Plot of post-surgery ground reaction forces.

Supplementary Data to the article "Influence of surgical intervention on pre- and post-surgery patient specific muscle synergies in children with cerebral palsy", by Tiana Breust, Jiayin Lin, Vincent C. K. Cheung, Firooz Salami, Sebastian I. Wolf, Gursel Alici and Manish Sreenivasa

Online Resource 3e: Synergy Activation Profiles

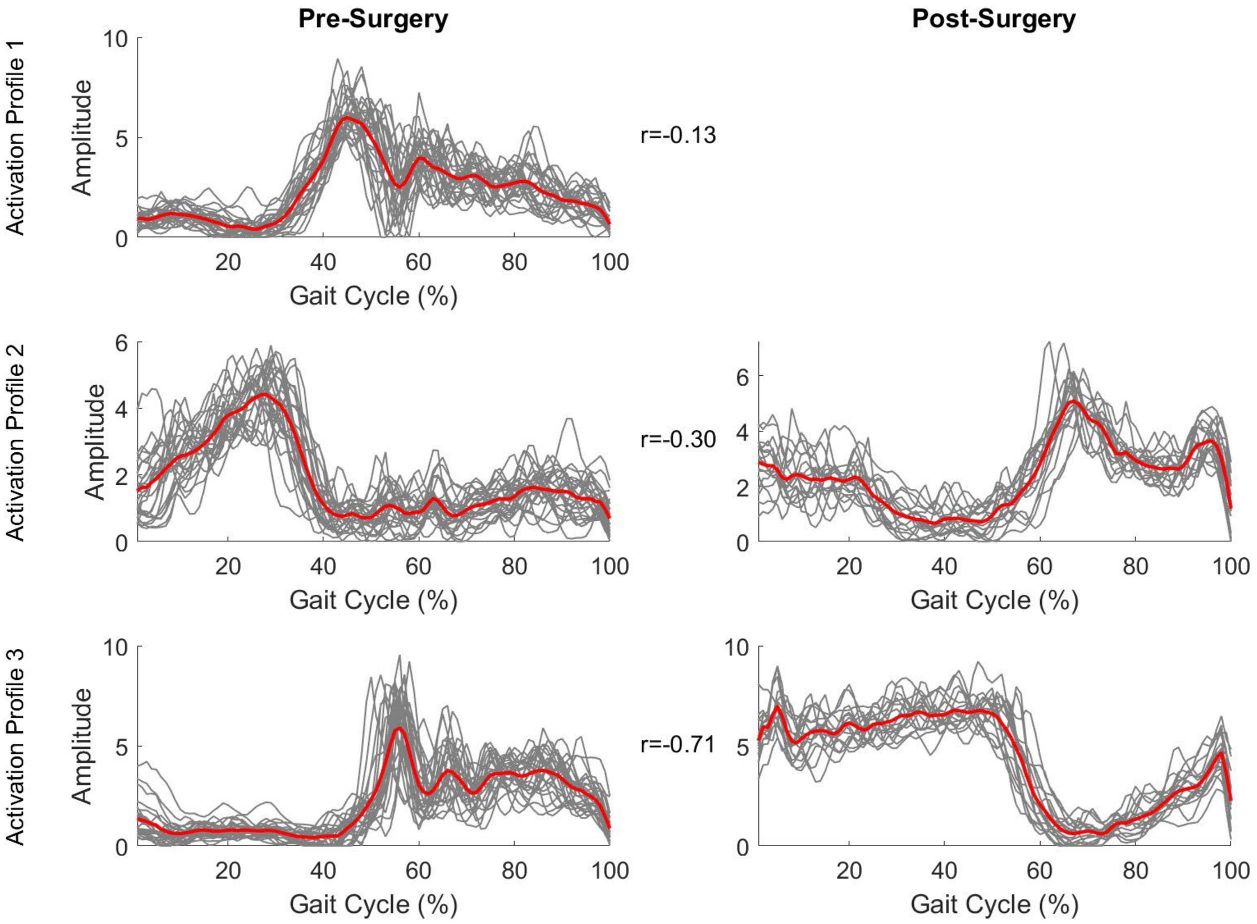

Figure 1: Patient specific correlation of synergy activation profiles as observed in P1 – Right. Pearson's correlation coefficient (r) is displayed for each synergy activation pair. For the unmatched synergy, correlation was determined for the activation profile that corresponds with the highest matched synergy from the weight similarity analysis.

Supplementary Data to the article "Influence of surgical intervention on pre- and post-surgery patient specific muscle synergies in children with cerebral palsy", by Tiana Breust, Jiayin Lin, Vincent C. K. Cheung, Firooz Salami, Sebastian I. Wolf, Gursel Alici and Manish Sreenivasa

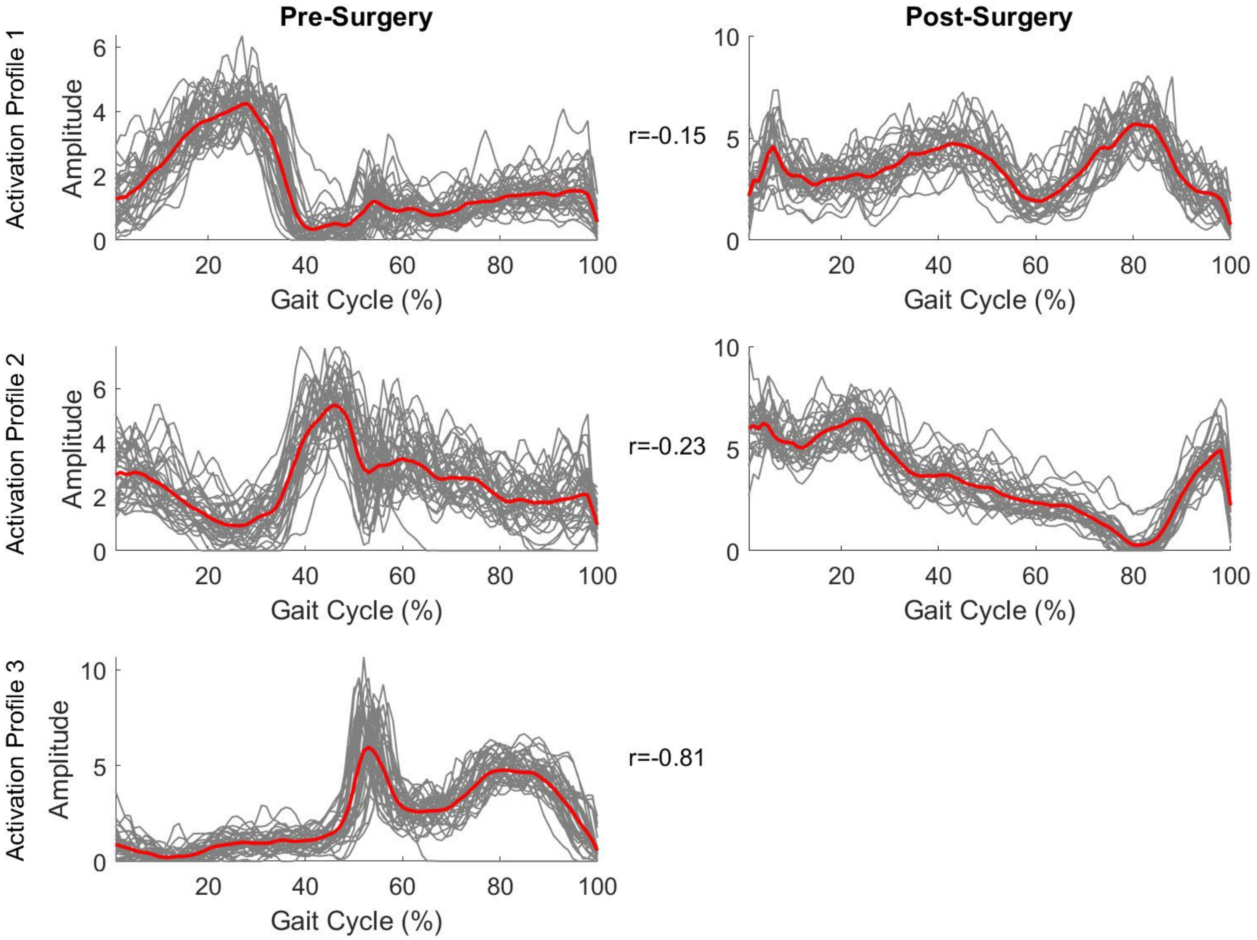

Figure 2: Patient specific correlation of synergy activation profiles as observed in P1 – Left. Pearson's correlation coefficient (r) is displayed for each synergy activation pair. For the unmatched synergy, correlation was determined for the activation profile that corresponds with the highest matched synergy from the weight similarity analysis.

Supplementary Data to the article "Influence of surgical intervention on pre- and post-surgery patient specific muscle synergies in children with cerebral palsy", by Tiana Breust, Jiayin Lin, Vincent C. K. Cheung, Firooz Salami, Sebastian I. Wolf, Gursel Alici and Manish Sreenivasa

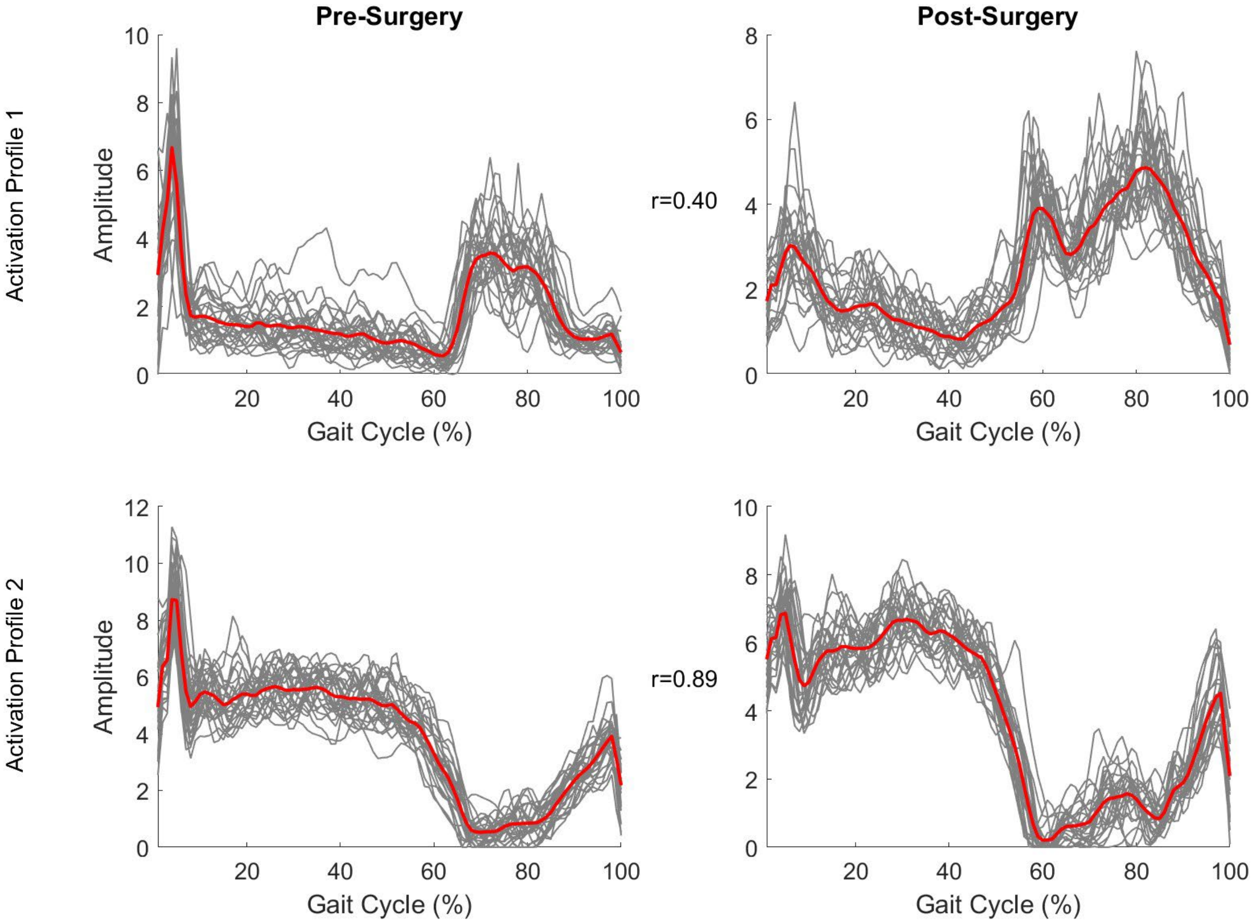

Figure 3: Patient specific correlation of synergy activation profiles as observed in P2 – Right. Pearson's correlation coefficient (r) is displayed for each synergy activation pair.

Supplementary Data to the article "Influence of surgical intervention on pre- and post-surgery patient specific muscle synergies in children with cerebral palsy", by Tiana Breust, Jiayin Lin, Vincent C. K. Cheung, Firooz Salami, Sebastian I. Wolf, Gursel Alici and Manish Sreenivasa

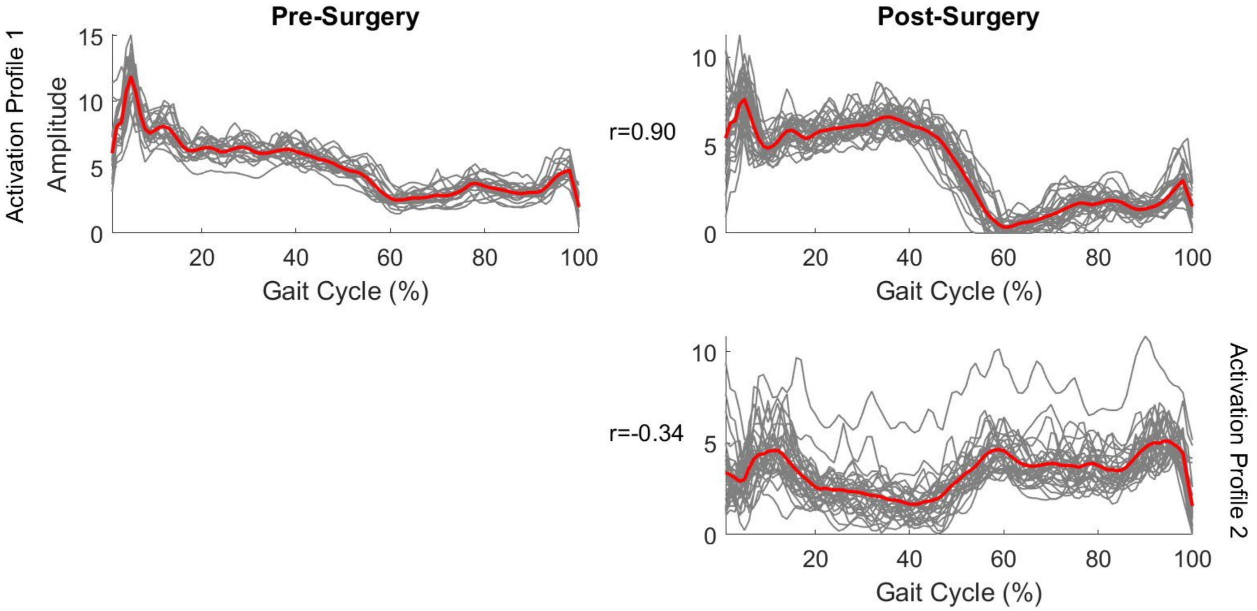

Figure 4: Patient specific correlation of synergy activation profiles as observed in P2 – Left. Pearson's correlation coefficient (r) is displayed for each synergy activation pair. For the unmatched synergy, correlation was determined for the activation profile that corresponds with the highest matched synergy from the weight similarity analysis.

Supplementary Data to the article "Influence of surgical intervention on pre- and post-surgery patient specific muscle synergies in children with cerebral palsy", by Tiana Breust, Jiayin Lin, Vincent C. K. Cheung, Firooz Salami, Sebastian I. Wolf, Gursel Alici and Manish Sreenivasa

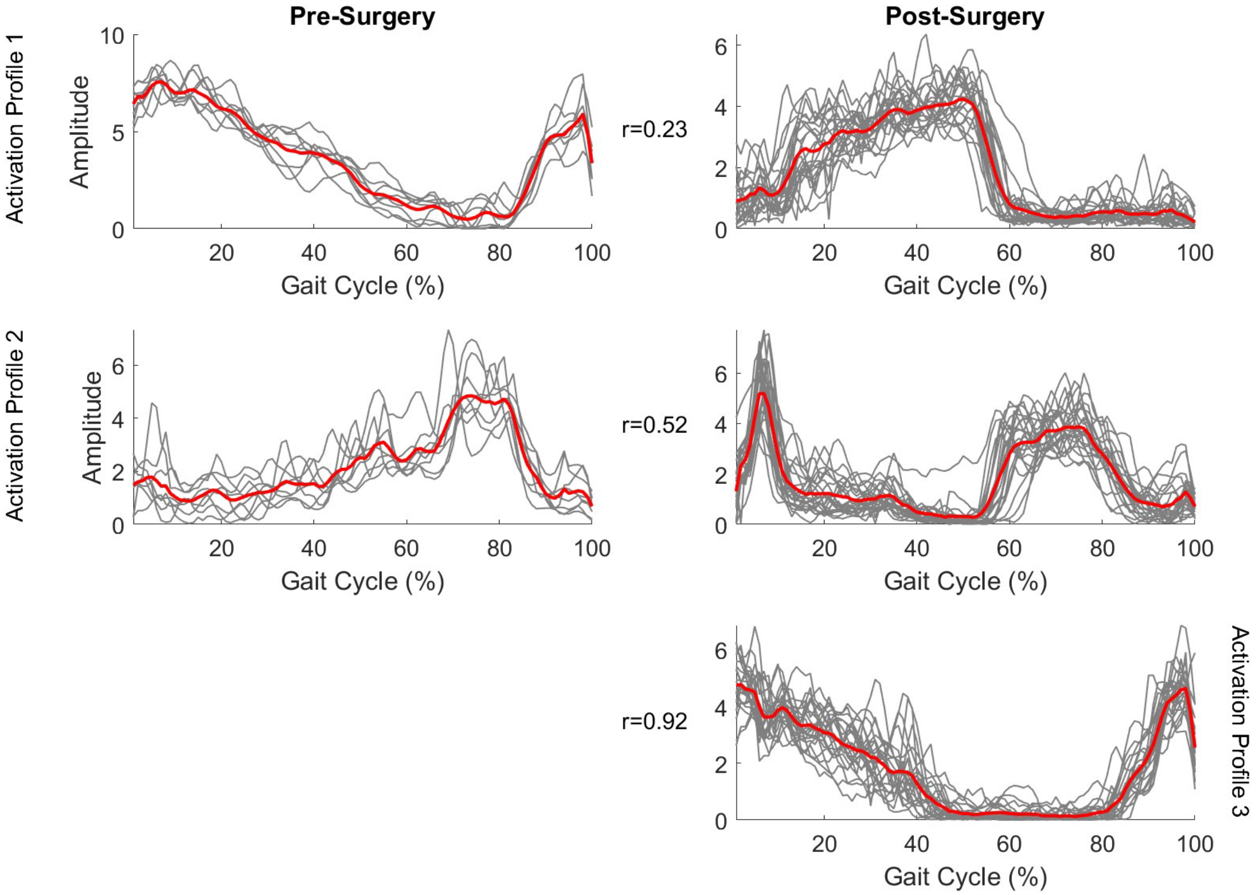

Figure 5: Patient specific correlation of synergy activation profiles as observed in P3 – Right. Pearson's correlation coefficient (r) is displayed for each synergy activation pair. For the unmatched synergy, correlation was determined for the activation profile that corresponds with the highest matched synergy from the weight similarity analysis.

Supplementary Data to the article "Influence of surgical intervention on pre- and post-surgery patient specific muscle synergies in children with cerebral palsy", by Tiana Breust, Jiayin Lin, Vincent C. K. Cheung, Firooz Salami, Sebastian I. Wolf, Gursel Alici and Manish Sreenivasa

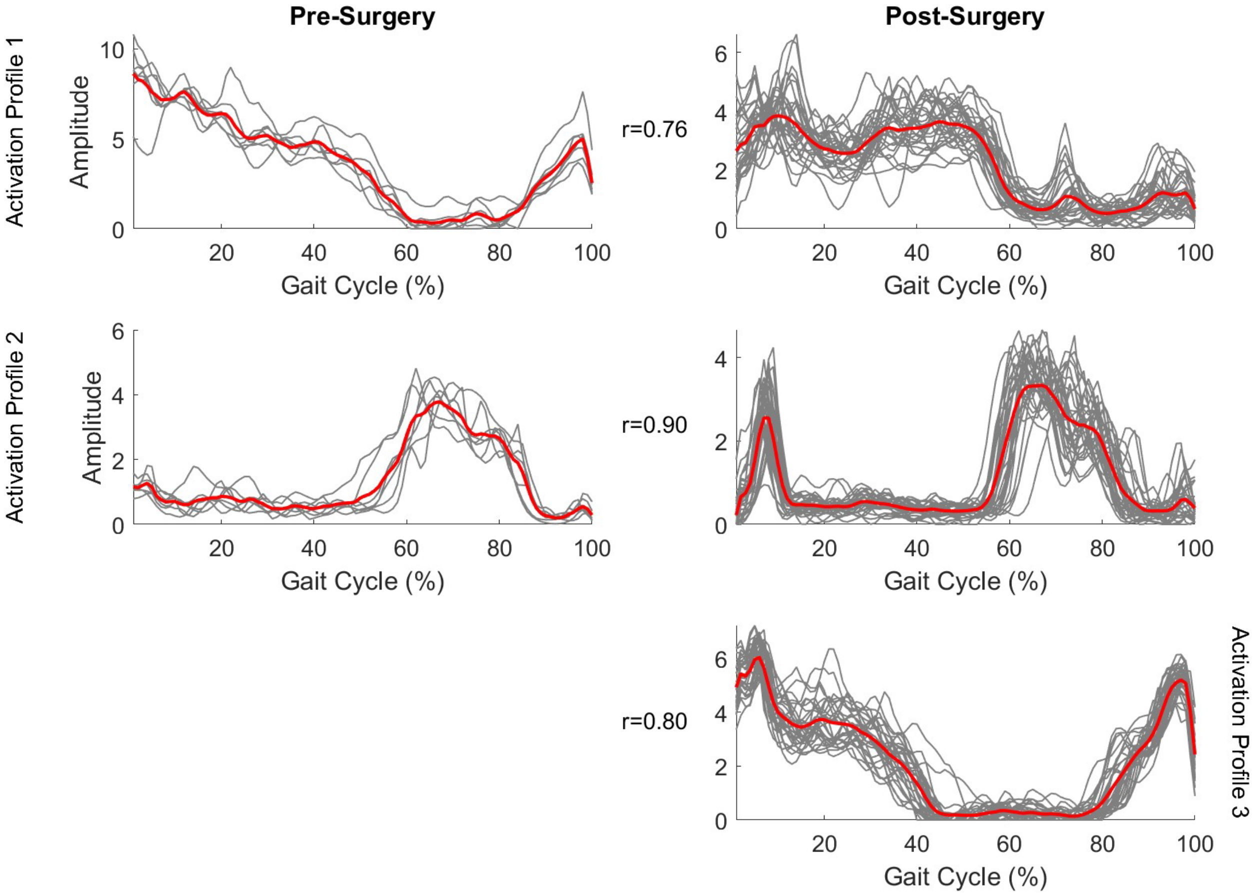

Figure 6: Patient specific correlation of synergy activation profiles as observed in P3 – Left. Pearson's correlation coefficient (r) is displayed for each synergy activation pair. For the unmatched synergy, correlation was determined for the activation profile that corresponds with the highest matched synergy from the weight similarity analysis.

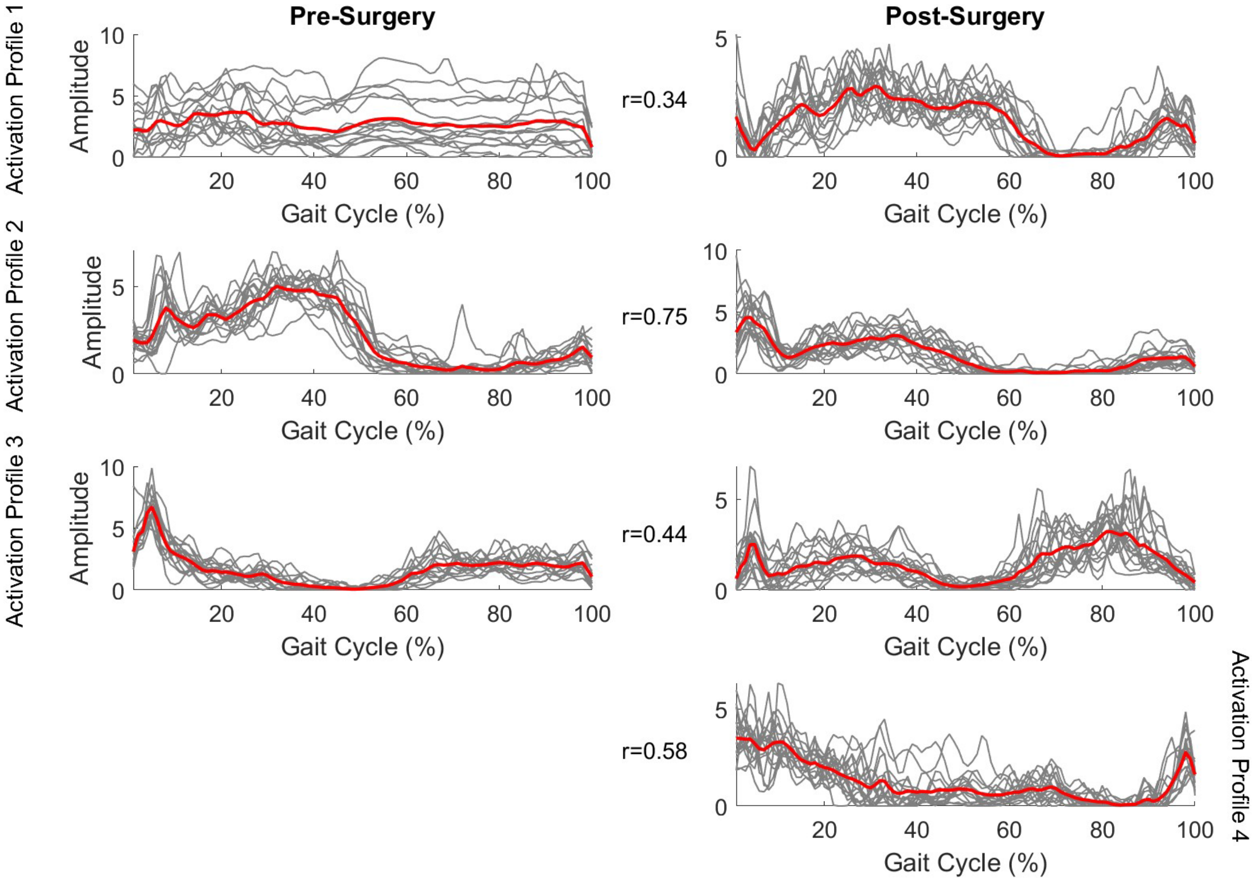

Figure 7: Patient specific correlation of synergy activation profiles as observed in P4 – Right. Pearson's correlation coefficient (r) is displayed for each synergy activation pair. For the unmatched synergy, correlation was determined for the activation profile that corresponds with the highest matched synergy from the weight similarity analysis.

Supplementary Data to the article "Influence of surgical intervention on pre- and post-surgery patient specific muscle synergies in children with cerebral palsy", by Tiana Breust, Jiayin Lin, Vincent C. K. Cheung, Firooz Salami, Sebastian I. Wolf, Gursel Alici and Manish Sreenivasa

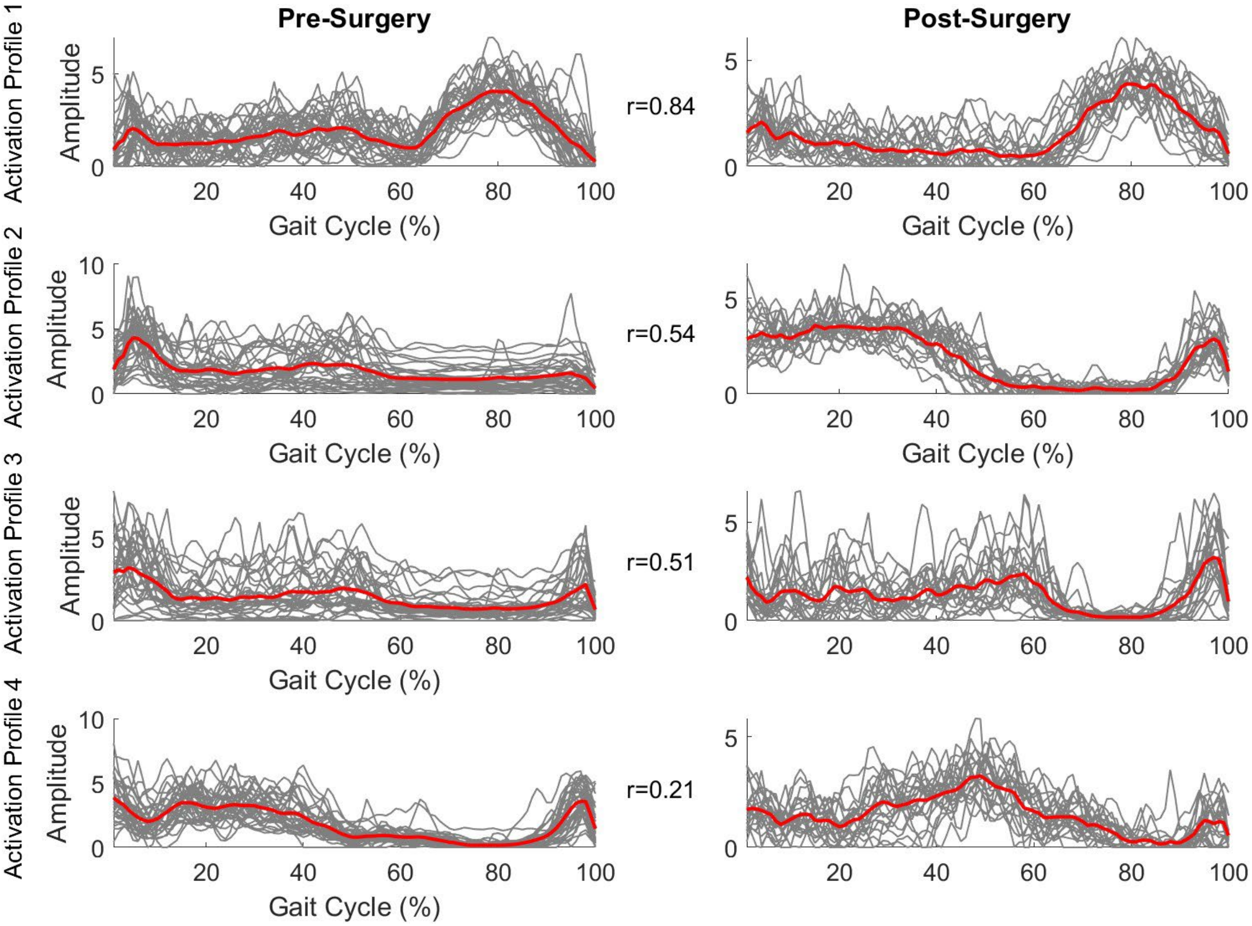

Figure 8: Patient specific correlation of synergy activation profiles as observed in P4 – Left. Pearson's correlation coefficient (r) is displayed for each synergy activation pair.

Supplementary Data to the article "Influence of surgical intervention on pre- and post-surgery patient specific muscle synergies in children with cerebral palsy", by Tiana Breust, Jiayin Lin, Vincent C. K. Cheung, Firooz Salami, Sebastian I. Wolf, Gursel Alici and Manish Sreenivasa

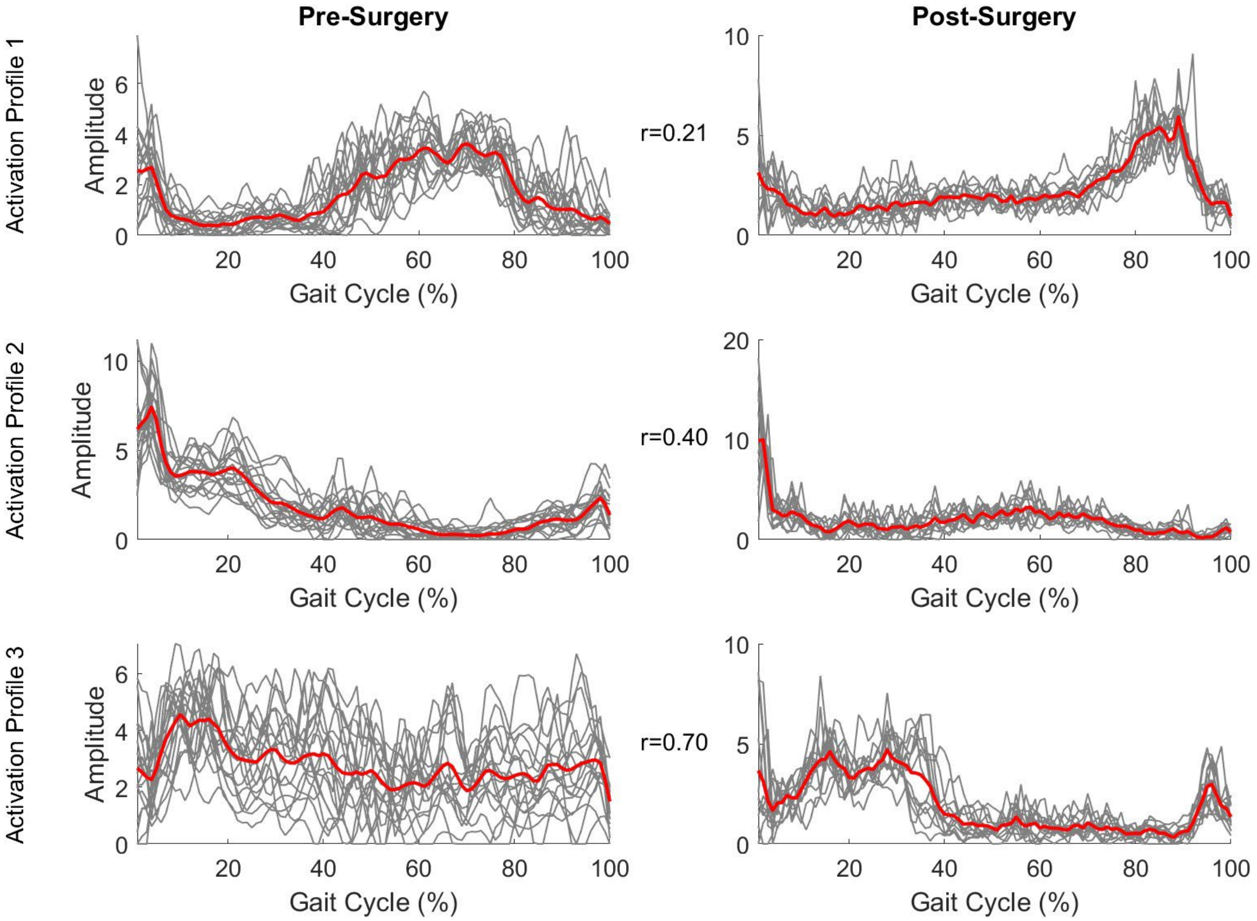

Figure 9: Patient specific correlation of synergy activation profiles as observed in P5 – Right. Pearson's correlation coefficient (r) is displayed for each synergy activation pair.

Supplementary Data to the article "Influence of surgical intervention on pre- and post-surgery patient specific muscle synergies in children with cerebral palsy", by Tiana Breust, Jiayin Lin, Vincent C. K. Cheung, Firooz Salami, Sebastian I. Wolf, Gursel Alici and Manish Sreenivasa

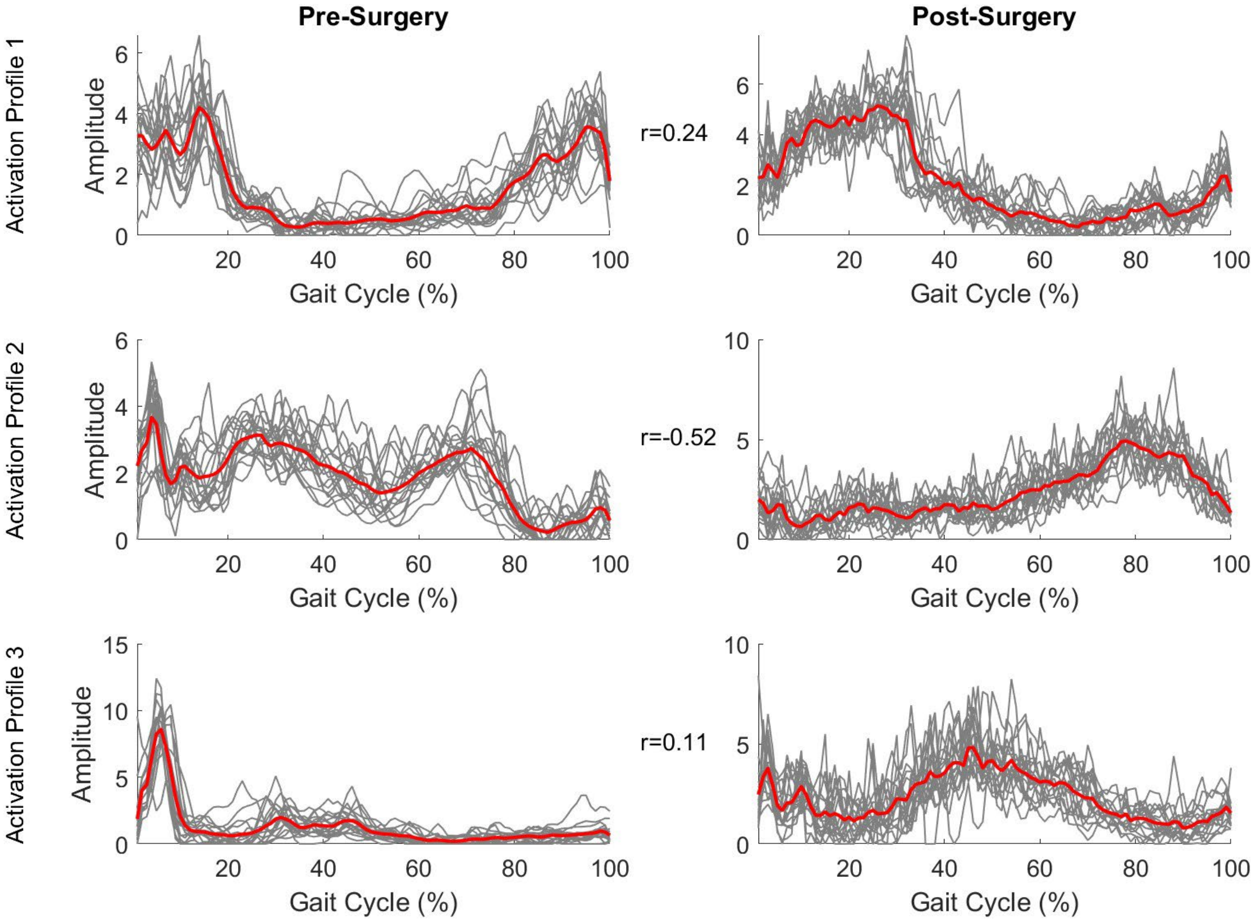

Figure 10: Patient specific correlation of synergy activation profiles as observed in P5 – Left. Pearson's correlation coefficient (r) is displayed for each synergy activation pair.

Supplementary Data to the article "Influence of surgical intervention on pre- and post-surgery patient specific muscle synergies in children with cerebral palsy", by Tiana Breust, Jiayin Lin, Vincent C. K. Cheung, Firooz Salami, Sebastian I. Wolf, Gursel Alici and Manish Sreenivasa

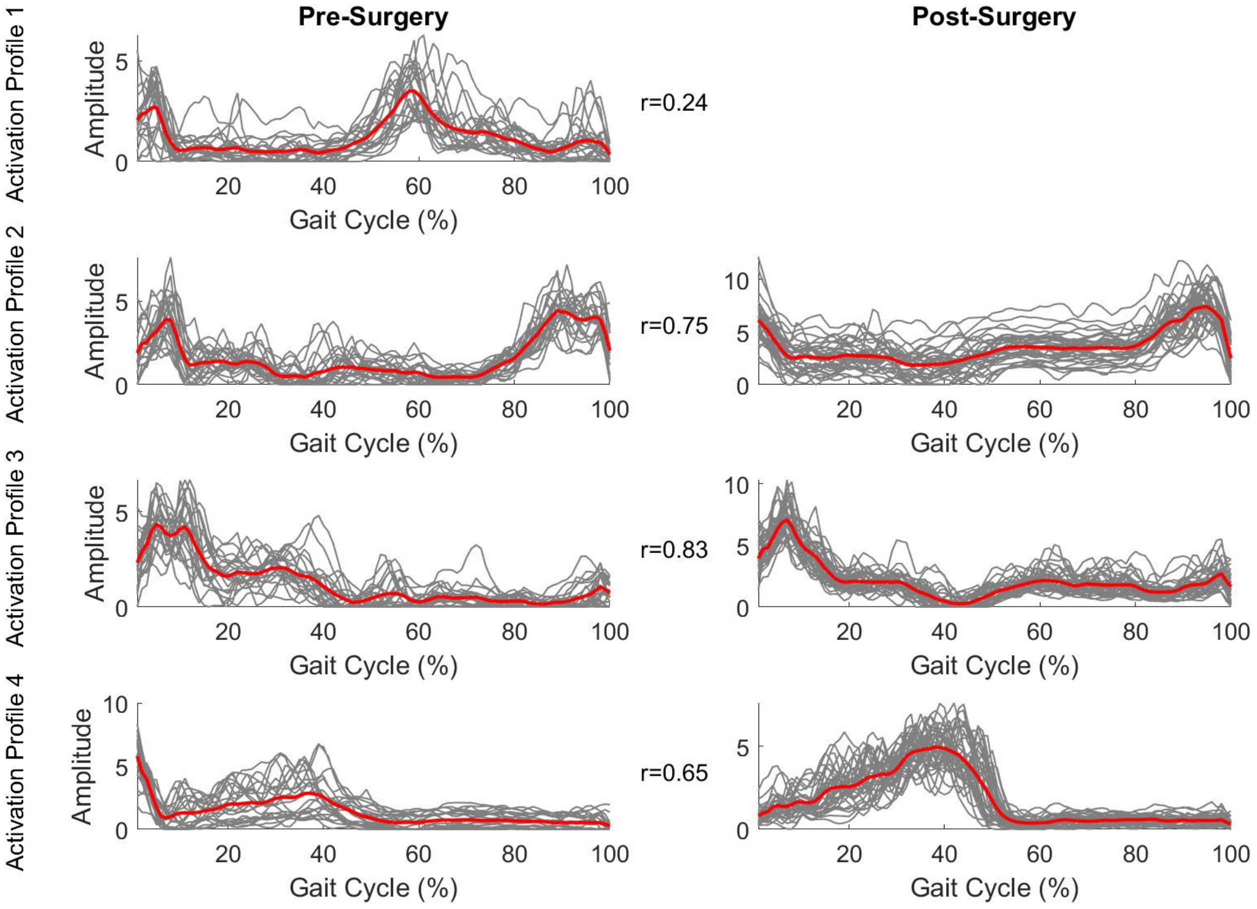

Figure 11: Patient specific correlation of synergy activation profiles as observed in P6 – Right. Pearson's correlation coefficient (r) is displayed for each synergy activation pair. For the unmatched synergy, correlation was determined for the activation profile that corresponds with the highest matched synergy from the weight similarity analysis.

Supplementary Data to the article "Influence of surgical intervention on pre- and post-surgery patient specific muscle synergies in children with cerebral palsy", by Tiana Breust, Jiayin Lin, Vincent C. K. Cheung, Firooz Salami, Sebastian I. Wolf, Gursel Alici and Manish Sreenivasa

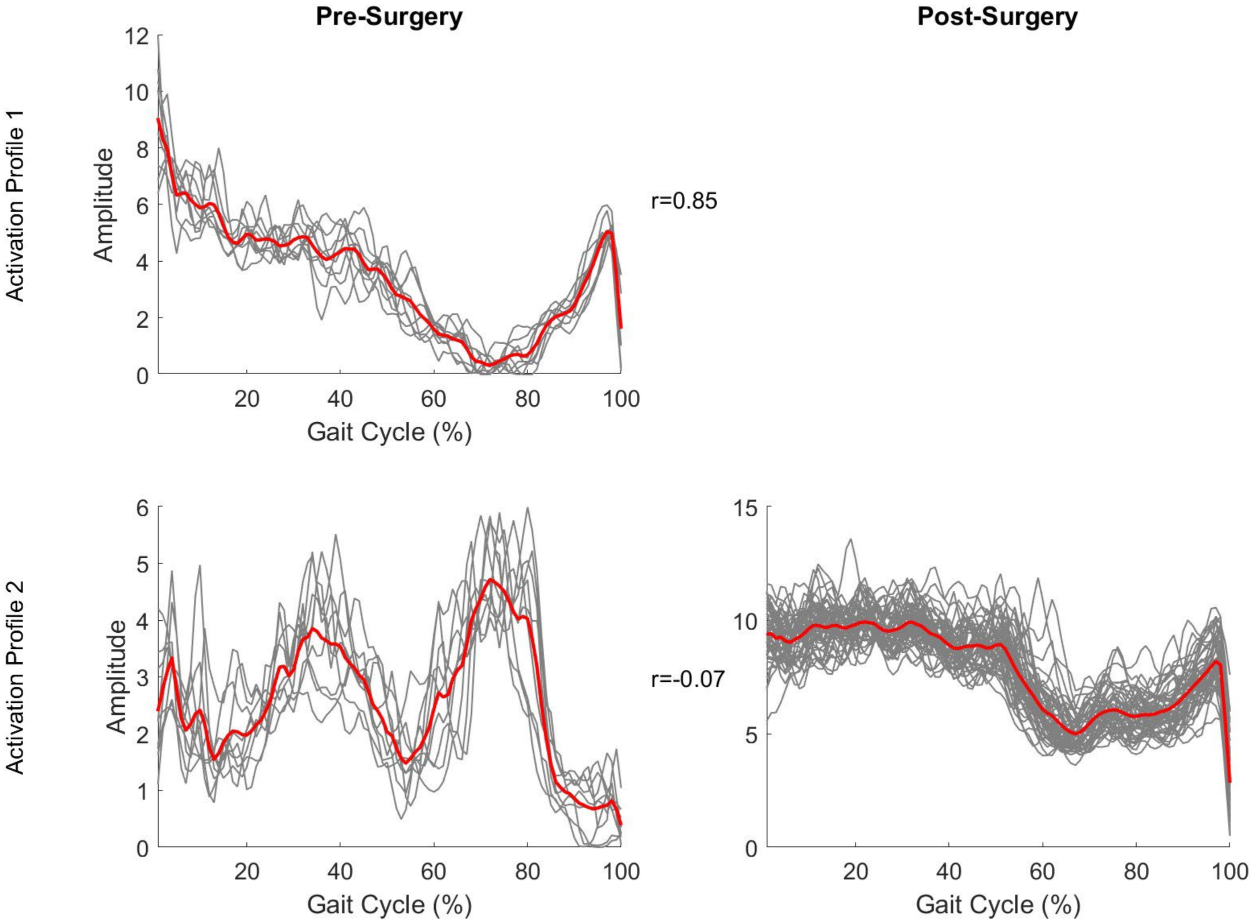

Figure 12: Patient specific correlation of synergy activation profiles as observed in P7 – Right. Pearson's correlation coefficient (r) is displayed for each synergy activation pair. For the unmatched synergy, correlation was determined for the activation profile that corresponds with the highest matched synergy from the weight similarity analysis.

Supplementary Data to the article "Influence of surgical intervention on pre- and post-surgery patient specific muscle synergies in children with cerebral palsy", by Tiana Breust, Jiayin Lin, Vincent C. K. Cheung, Firooz Salami, Sebastian I. Wolf, Gursel Alici and Manish Sreenivasa

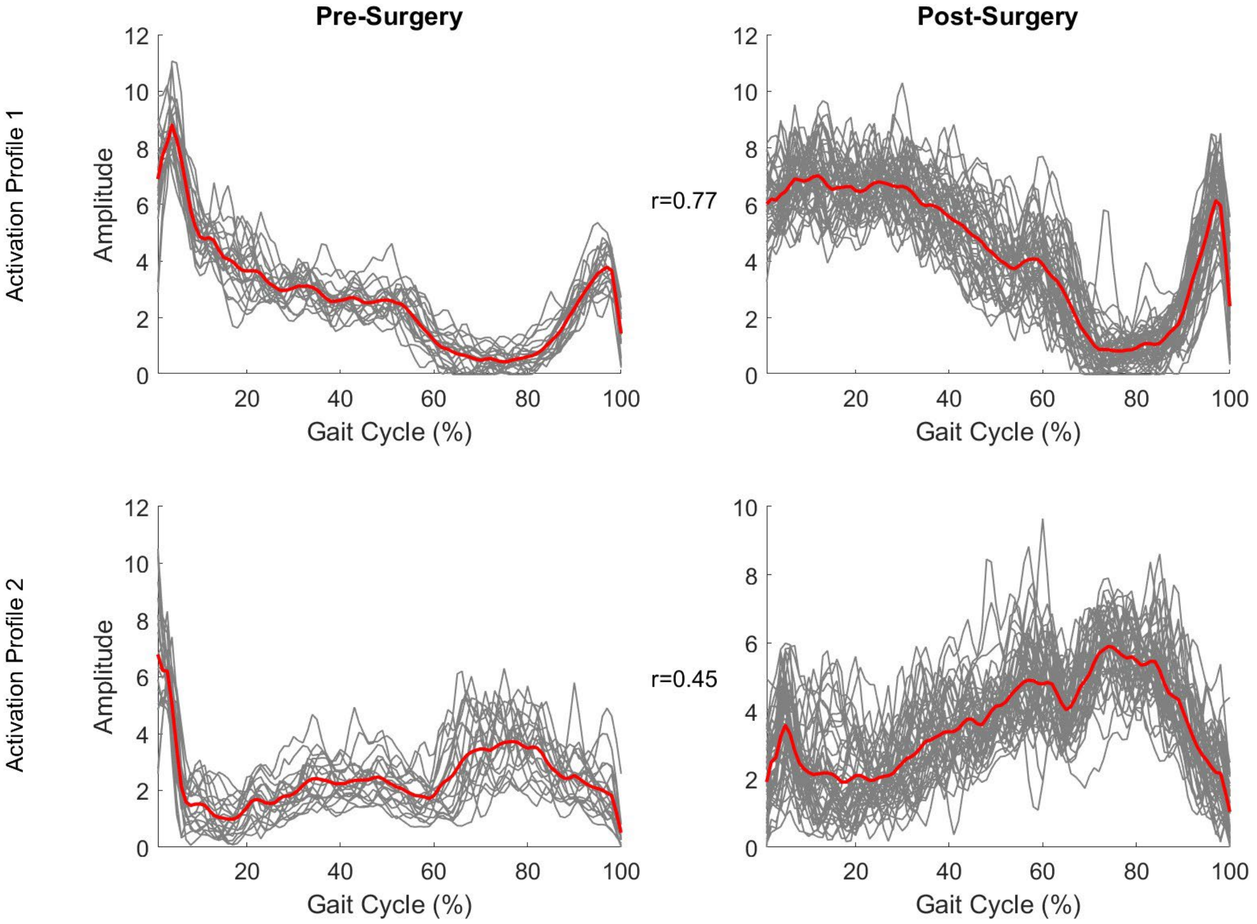

Figure 13: Patient specific correlation of synergy activation profiles as observed in P7 – Left. Pearson's correlation coefficient (r) is displayed for each synergy activation pair.

Supplementary Data to the article "Influence of surgical intervention on pre- and post-surgery patient specific muscle synergies in children with cerebral palsy", by Tiana Breust, Jiayin Lin, Vincent C. K. Cheung, Firooz Salami, Sebastian I. Wolf, Gursel Alici and Manish Sreenivasa

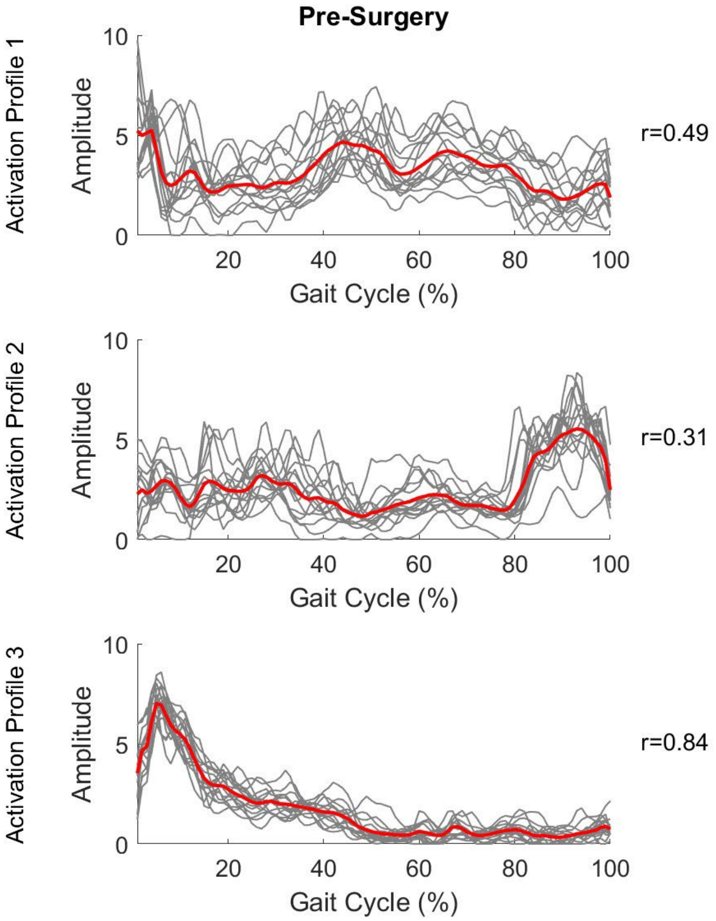

Figure 14: Patient specific correlation of synergy activation profiles as observed in P8 – Right. Pearson's correlation coefficient (r) is displayed for each synergy activation pair. For the unmatched synergy, correlation was determined for the activation profile that corresponds with the highest matched synergy from the weight similarity analysis.

Supplementary Data to the article "Influence of surgical intervention on pre- and post-surgery patient specific muscle synergies in children with cerebral palsy", by Tiana Breust, Jiayin Lin, Vincent C. K. Cheung, Firooz Salami, Sebastian I. Wolf, Gursel Alici and Manish Sreenivasa

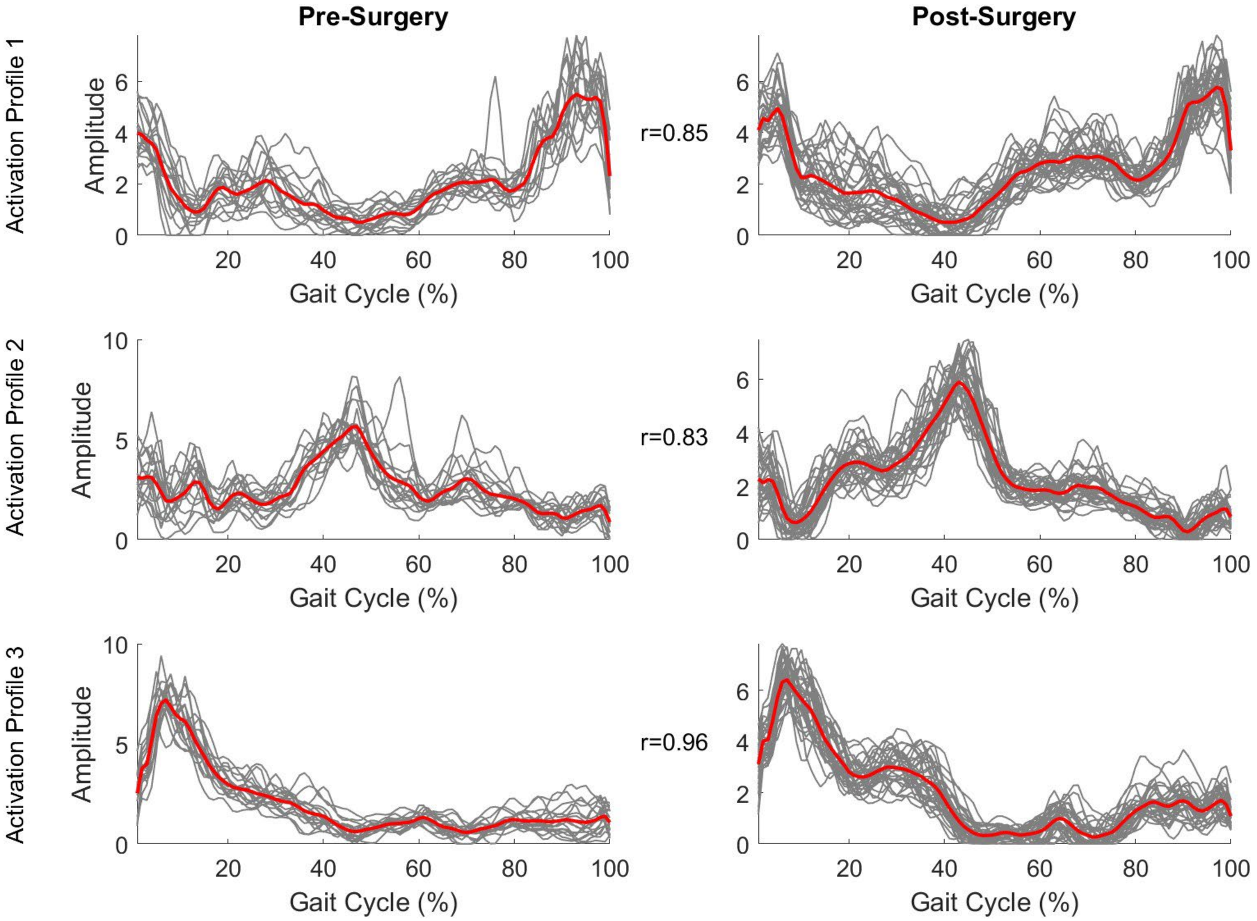

Figure 15: Patient specific correlation of synergy activation profiles as observed in P8 – Left. Pearson's correlation coefficient (r) is displayed for each synergy activation pair.

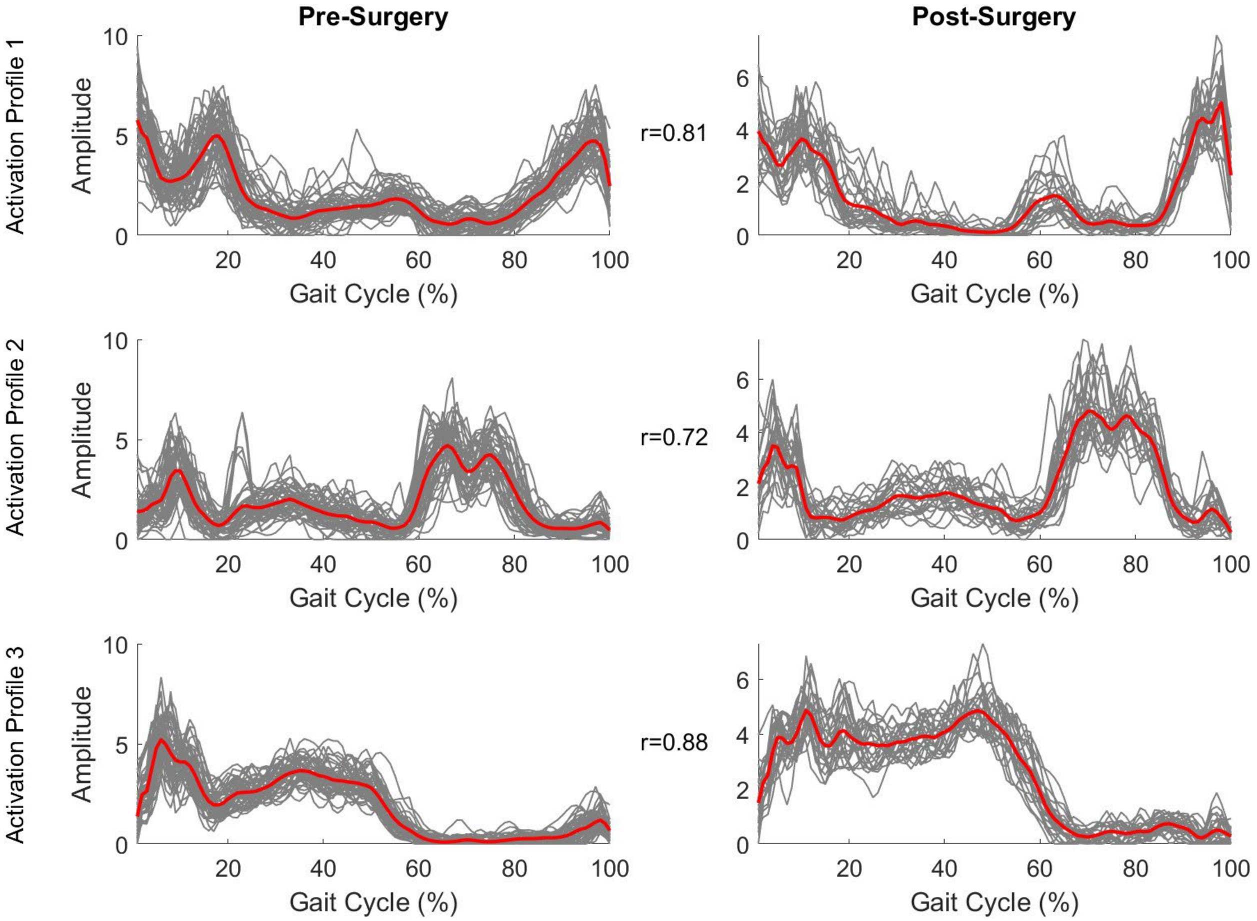

Figure 16: Patient specific correlation of synergy activation profiles as observed in P9 – Right. Pearson's correlation coefficient (r) is displayed for each synergy activation pair.

Supplementary Data to the article "Influence of surgical intervention on pre- and post-surgery patient specific muscle synergies in children with cerebral palsy", by Tiana Breust, Jiayin Lin, Vincent C. K. Cheung, Firooz Salami, Sebastian I. Wolf, Gursel Alici and Manish Sreenivasa

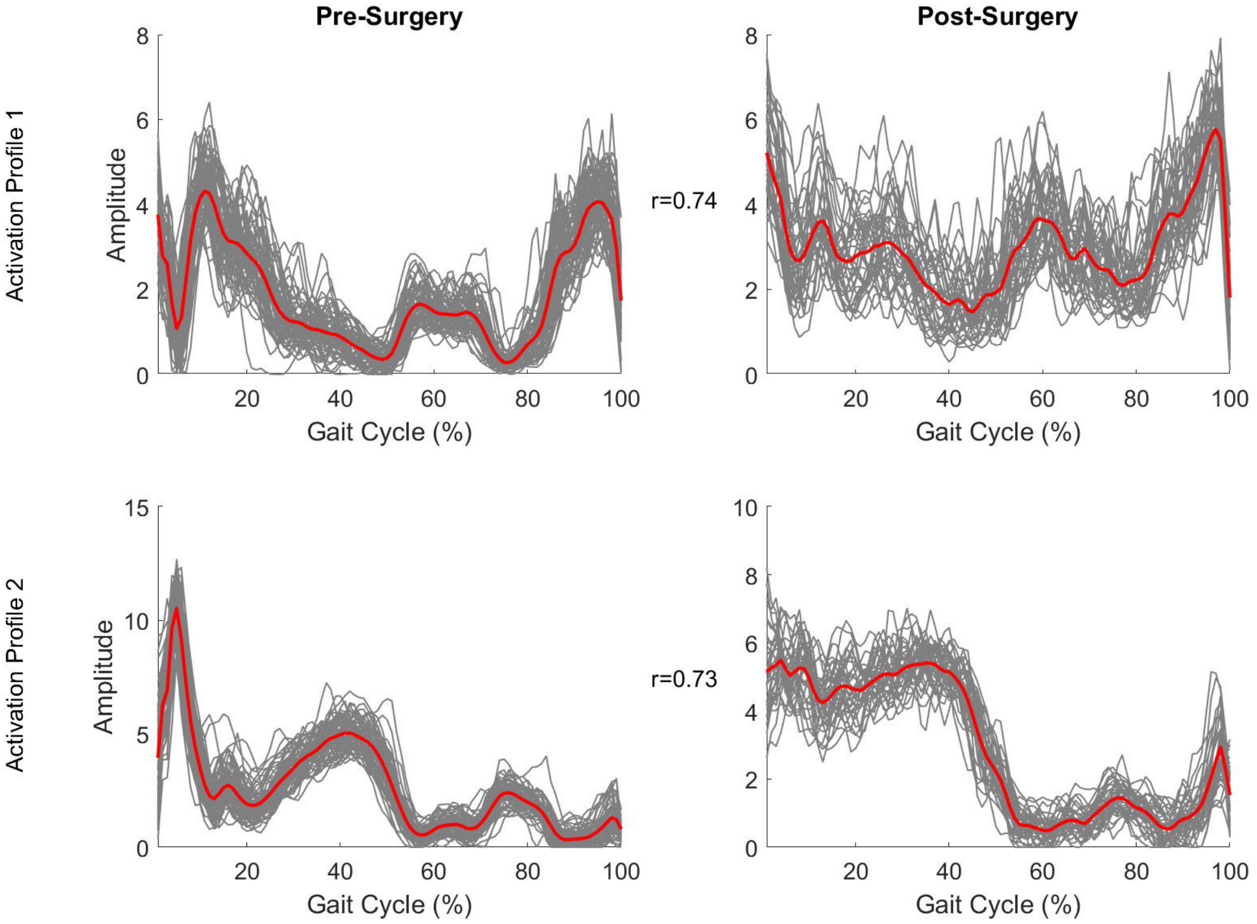

Figure 17: Patient specific correlation of synergy activation profiles as observed in P9 – Left. Pearson's correlation coefficient (r) is displayed for each synergy activation pair.

Supplementary Data to the article "Influence of surgical intervention on pre- and post-surgery patient specific muscle synergies in children with cerebral palsy", by Tiana Breust, Jiayin Lin, Vincent C. K. Cheung, Firooz Salami, Sebastian I. Wolf, Gursel Alici and Manish Sreenivasa

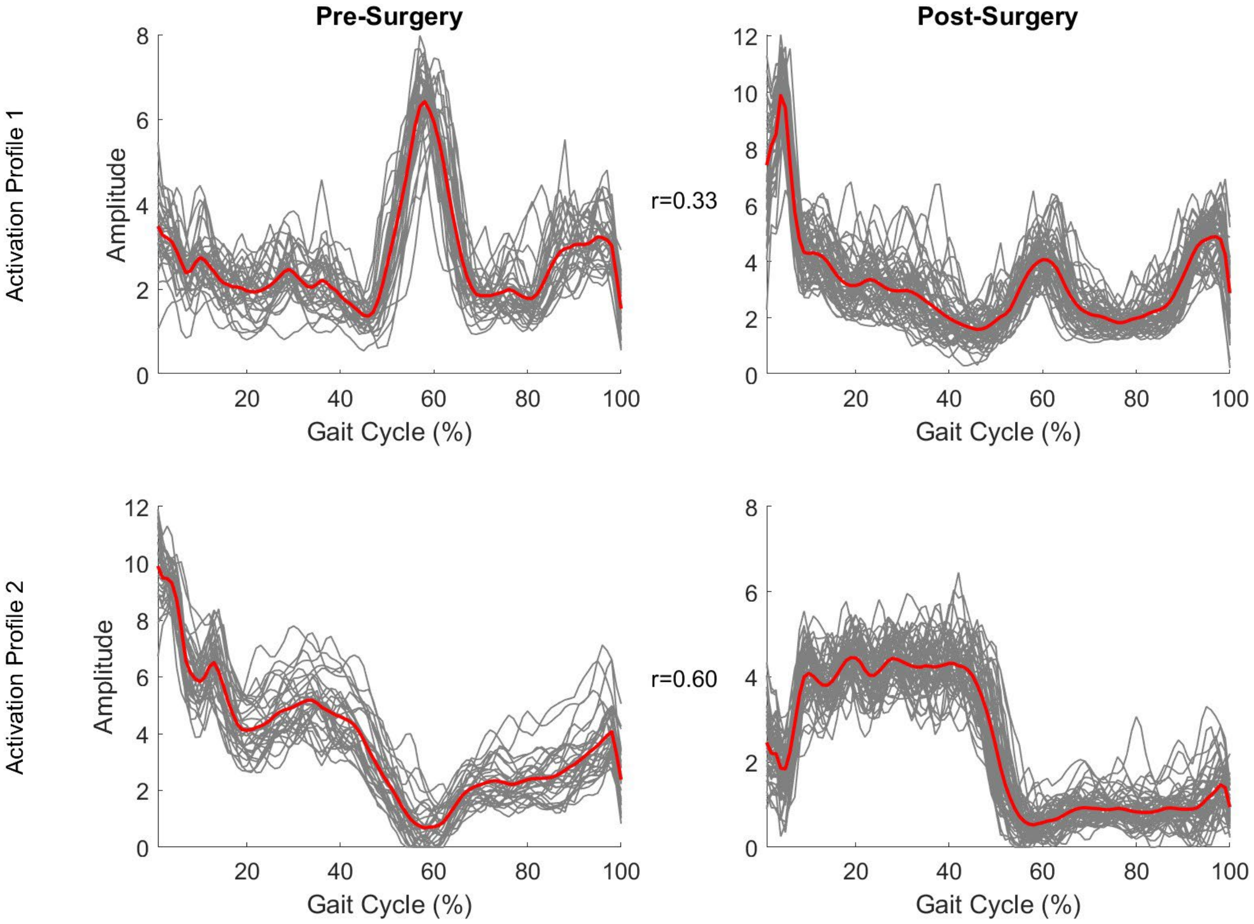

Figure 18: Patient specific correlation of synergy activation profiles as observed in P10 – Right. Pearson's correlation coefficient (r) is displayed for each synergy activation pair.

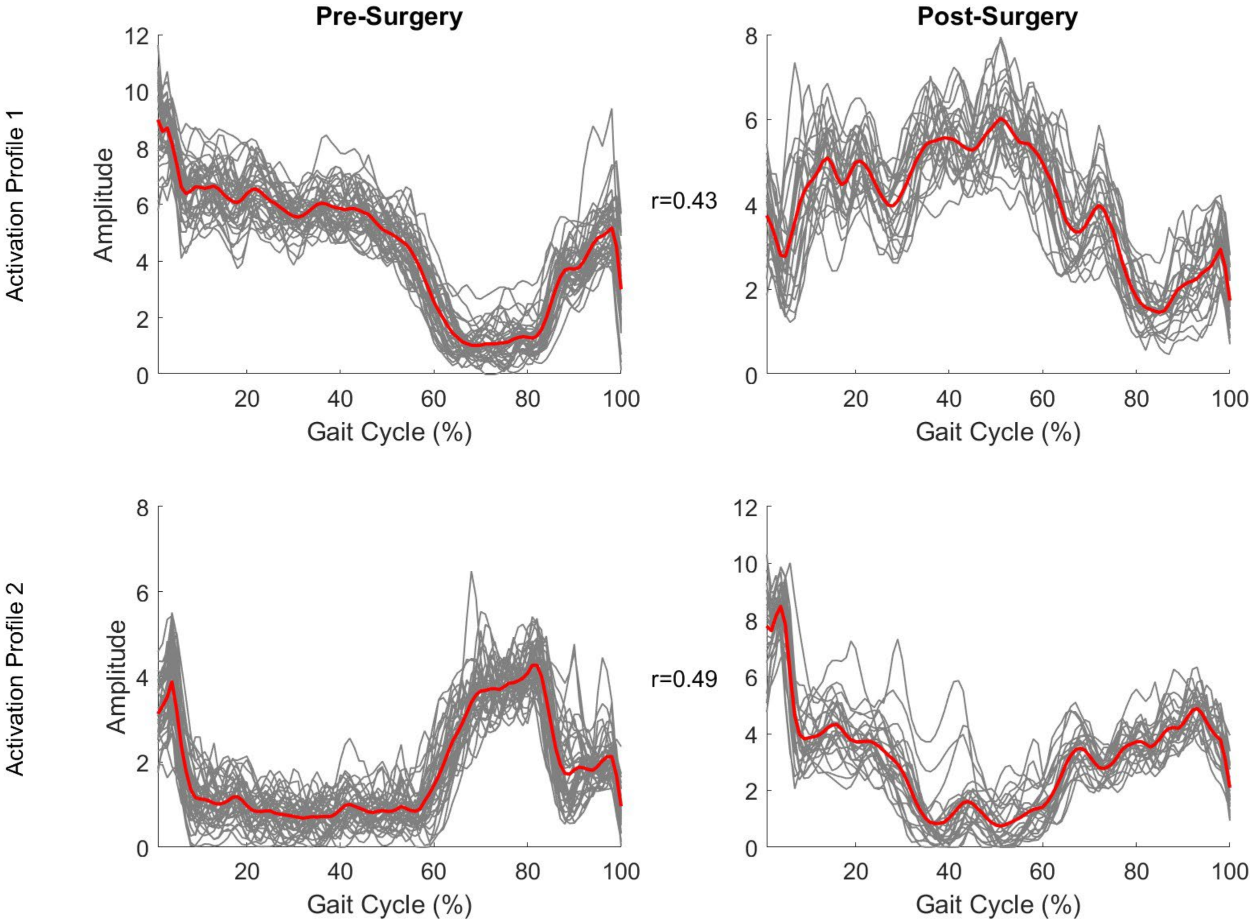

Figure 19: Patient specific correlation of synergy activation profiles as observed in P11 – Right. Pearson's correlation coefficient (r) is displayed for each synergy activation pair.

Supplementary Data to the article "Influence of surgical intervention on pre- and post-surgery patient specific muscle synergies in children with cerebral palsy", by Tiana Breust, Jiayin Lin, Vincent C. K. Cheung, Firooz Salami, Sebastian I. Wolf, Gursel Alici and Manish Sreenivasa

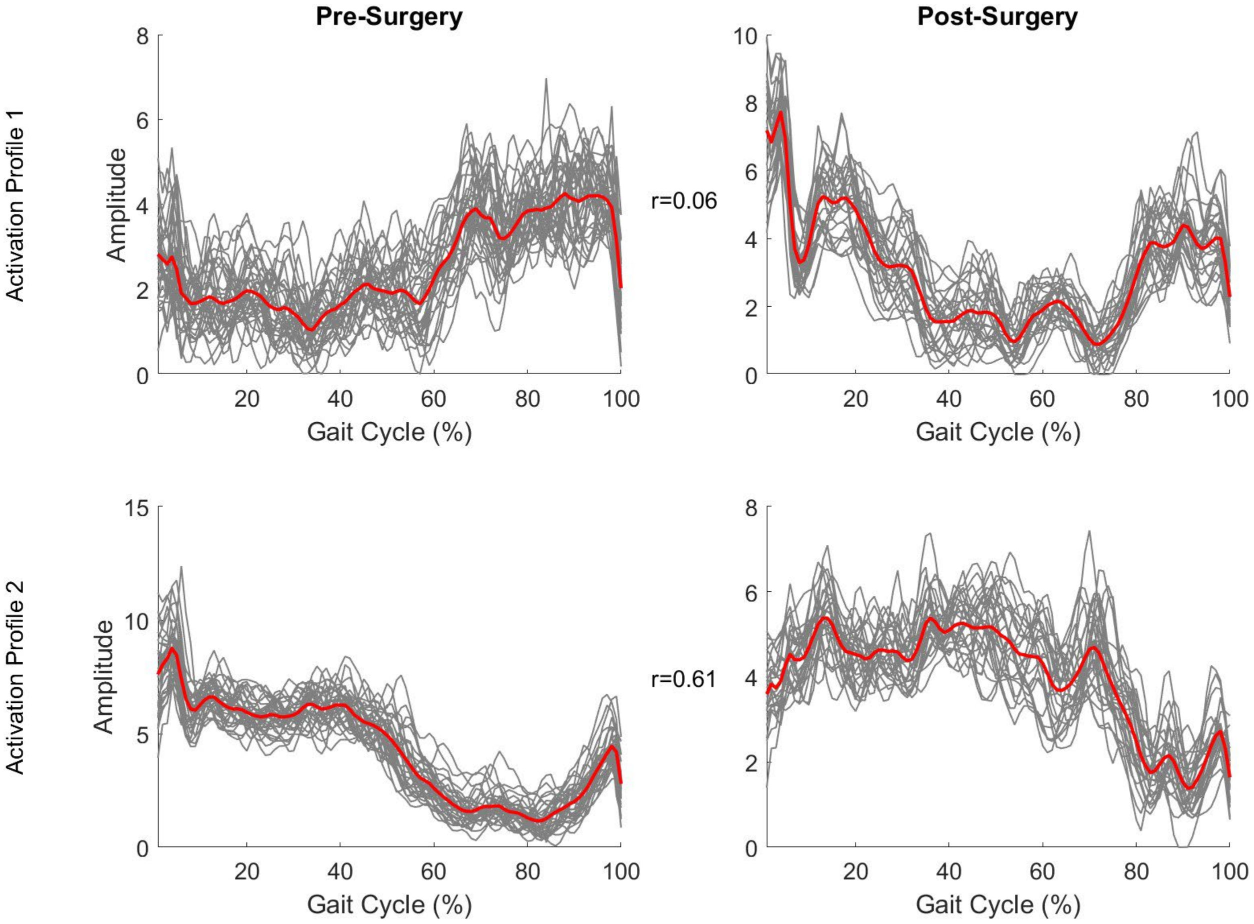

Figure 20: Patient specific correlation of synergy activation profiles as observed in P11 – Left. Pearson's correlation coefficient (r) is displayed for each synergy activation pair.
